# The catechol moiety of obafluorin is essential for antibacterial activity†

**DOI:** 10.1039/d3cb00127j

**Published:** 2023-08-21

**Authors:** Sibyl F. D. Batey, Melissa J. Davie, Edward S. Hems, Jonathon D. Liston, Thomas A. Scott, Silke Alt, Christopher S. Francklyn, Barrie Wilkinson

**Affiliations:** a Department of Molecular Microbiology, John Innes Centre Norwich Research Park Norwich NR4 7UH UK barrie.wilkinson@jic.ac.uk; b Department of Biochemistry, College of Medicine, University of Vermont, Burlington Vermont 05405 USA christopher.francklyn@med.uvm.edu

## Abstract

Obafluorin is a *Pseudomonas fluorescens* antibacterial natural product that inhibits threonyl-tRNA synthetase (ThrRS). It acts as a broad-spectrum antibiotic against a range of clinically relevant pathogens and comprises a strained β-lactone ring decorated with catechol and 4-nitro-benzyl moieties. The catechol moiety is widespread in nature and its role in the coordination of ferric iron has been well-characterised in siderophores and Trojan horse antibiotics. Here we use a combination of mutasynthesis, bioassays, enzyme assays and metal binding studies to delineate the role of the catechol moiety in the bioactivity of obafluorin. We use *P. fluorescens* biosynthetic mutants to generate obafluorin analogues with modified catechol moieties. We demonstrate that an intact catechol is required for both antibacterial activity and inhibition of the ThrRS molecular target. Although recent work showed that the obafluorin catechol coordinates Zn^2+^ in the ThrRS active site, we find that obafluorin is a weak Zn^2+^ binder *in vitro*, contrasting with a strong, specific 1 : 1 interaction with Fe^3+^. We use bioassays with siderophore transporter mutants to probe the role of the obafluorin catechol in Fe^3+^-mediated uptake. Surprisingly, obafluorin does not behave as a Trojan horse antibiotic but instead exhibits increased antibacterial activity in the presence of Fe^3+^. We further demonstrate that Fe^3+^ binding prevents the hydrolytic breakdown of the β-lactone ring, revealing a hitherto unreported function for the catechol moiety in natural product bioactivity.

## Abstract 2

Complex formation with ferric iron protects the β-lactone moiety of obafluorin from hydrolysis and potentiates antibiotic activity.

## Graphical abstract

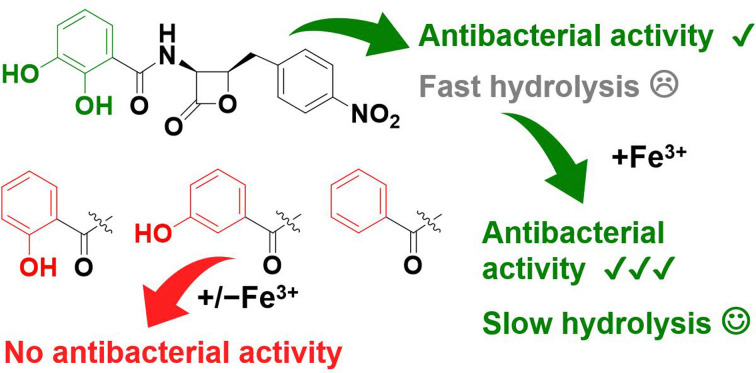

## Introduction

The over-use and misuse of antibiotics over the last 50 years has led to an alarming rise in antimicrobial resistance (AMR), which is arguably the greatest medical challenge humans will face this century. This worrying development has been highlighted by several high-profile reports, emphasising the need to increase the supply of antimicrobials effective against drug-resistant microorganisms.^1–4^ Almost a century after the discovery of penicillin, the major source of clinically used antibiotics remains specialised metabolites (natural products) from microbes, most commonly soil bacteria.^5^ As our arsenal against human pathogens becomes ever more depleted, natural products with promising bioactivity that were previously discarded due to instability, toxicity or a narrow spectrum of activity have been revisited.^6–8^

One such example is obafluorin (1), a structurally unique β-lactone antibiotic produced by *Pseudomonas fluorescens* ATCC 39502 (hereafter *P. fluorescens* WT).^9,10^ Although 1 undergoes facile ring-opening in the presence of nucleophiles, and under mildly basic conditions, it exhibits broad-spectrum antibacterial activity. It inhibits both Gram-positive and Gram-negative clinically relevant pathogens, including methicillin-resistant *Staphylococcus aureus* (MRSA), *E. coli* and *Pseudomonas aeruginosa*,^9,11,12^ and when dosed systemically can protect mice infected with *Streptococcus pyogenes* with no observable toxicity.^10^

We previously showed that the target of 1 in sensitive organisms is threonyl-tRNA synthetase (ThrRS).^12^ ThrRS is one of a suite of essential housekeeping aminoacyl tRNA synthetase (aaRS) enzymes, each one responsible for loading a specific amino acid on to its cognate tRNA.^13^ In ThrRS, an essential Zn^2+^ ion in the active site enables the discrimination between threonine and serine for effective translation.^14^ Despite relatively high conservation of aaRSs across the bacterial kingdom, divergencies have been exploited by natural product antibiotics that selectively target those of competing organisms.^15,16^ The LeuRS inhibitor Agrocin 84 is used to treat the plant pathogen crown gall disease^17^ and the IleRS inhibitor mupirocin is used clinically as a topical treatment for skin infections.^18^ In addition to 1, ThrRS is also targeted by borrelidin, a structurally distinct polyketide natural product, which acts by simultaneously occupying the threonine, ATP and tRNA catalytic subsites.^19,20^1-Producing strains carry *obaO*, an additional copy of ThrRS encoded by the 1 biosynthetic gene cluster (BGC), which can confer transferable resistance to 1-sensitive bacterial strains^12^ This immunity strategy has been observed for other aaRS-targeting natural products.^21–23^

The biosynthesis of 1 in *P. fluorescens* has been delineated in full and proceeds by the condensation of 2,3-dihydroxybenzoic acid (2,3-DHBA) and (2*S*,3*R*)-2-amino-3-hydroxy-4-(4-nitrophenyl)butanoate (AHNB) catalysed by the non-ribosomal peptide synthetase (NRPS) ObaI (alternatively named ObiF1), with product release occurring *via* the formation of the β-lactone ring.^24–26^ We showed that the genes *obaJLN* are responsible for the biosynthesis of 2,3-DHBA, and that deletion of *obaL* leads to the abolition of 1 production. This phenotype can be rescued *via* the exogenous addition of 2,3-DHBA to *P. fluorescens* Δ*obaL* cultures.^24^ However, when the immunity determinant *obaO* is also absent, the addition of 2,3-DHBA instead abolishes growth.^12^

The 2,3-DHBA catechol moiety is a common siderophore motif^27^ that is observed in a number of natural products, including enterobactin,^28^ bacillibactin,^29^ vibriobactin,^30^ and myxochelin.^31^ Therefore, we hypothesised that metal binding may be involved with the mechanism of action of 1. This could be *via* interactions with the ThrRS target directly and/or facilitating uptake *via* a Trojan horse antibiotic (THA) strategy, whereby the catechol enables the compound to hijack the active siderophore uptake system to cross the otherwise impenetrable Gram-negative membrane.^32^ The aaRS inhibitors albomycin, microcin C-like compounds, and agrocin 84 have been shown to act as THAs.^33^ Once inside the cell, the THA is then able to act on its target, in the case of 1*via* the highly reactive β-lactone group that is essential for antibacterial activity, as shown previously^10^ and confirmed in this work.

Here, we used a mutasynthesis strategy to produce analogues of 1 with modified catechol moieties. We demonstrate that these analogues are produced in the absence of the immunity determinant ObaO, with no significant detriment to growth, in stark contrast to 1 itself. We go on to purify these congeners, alongside ring-open 1 analogues which are accessed *via* chemical hydrolysis. Using a selection of bioindicator strains we demonstrate that the modified analogues lack bioactivity in cellular assays. We show that this is mirrored *in vitro* using aminoacylation assays with both the *E. coli* ThrRS (EcThrRS) and the *P. fluorescens* housekeeping ThrRS (PfThrRS) in the producer. Taken together, these data demonstrate that the catechol moiety is essential for 1 bioactivity, most likely *via* a direct interaction with the ThrRS target.

During the preparation of this manuscript, the interaction of the catechol with the target was verified by the publication of the crystal structure of 1 covalently bound to the *E. coli* ThrRS, in which the phenol groups of the catechol coordinate the essential Zn^2+^ ion present in the active site.^34^ We further demonstrate that despite this interaction in the molecular target, 1 shows weak binding of Zn^2+^ in solution, but instead shows a strong, specific 1 : 1 interaction with Fe^3+^. Surprisingly, this interaction did not appear to facilitate active cellular uptake, as experiments with *E. coli* mutants deficient in Fe^3+^ uptake transporters demonstrated 1 did not function as a THA. Instead, iron binding appears to prevent the hydrolytic breakdown of 1, which is associated with increased levels of the active lactone *versus* the ring-open form during production by *P. fluorescens* and with increased potency against sensitive organisms.

## Results and discussion

### Mutasynthesis of 1 analogues with modified catechol moieties

**Fig. 1:**
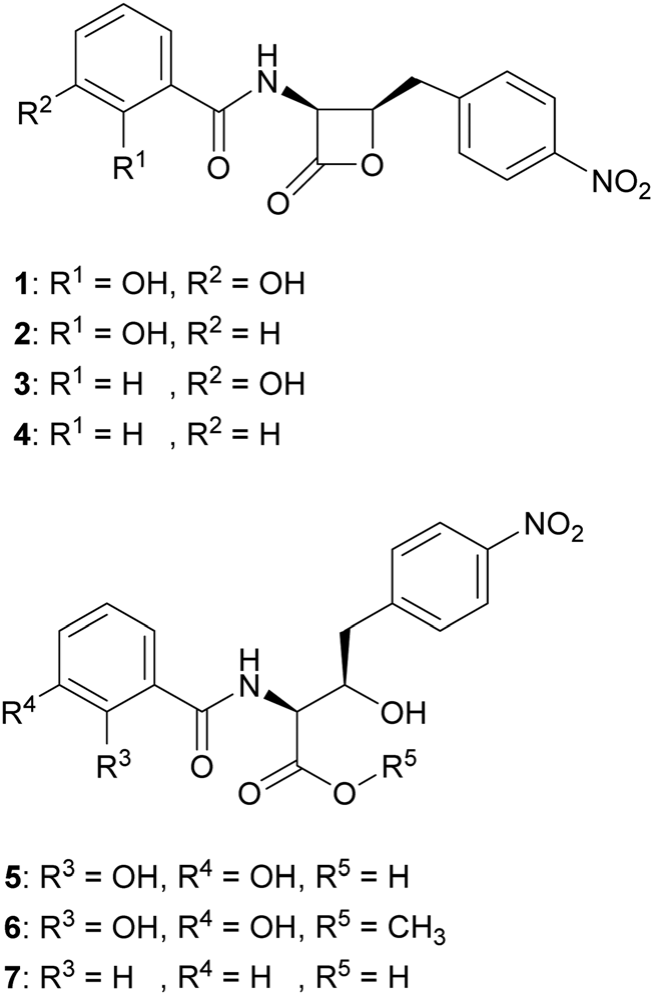
Structures of obafluorin and analogues. Obafluorin (1), 2-HBA-obafluorin (2), 3-HBA-obafluorin (3), BA-obafluorin (4), β-lactone hydrolysed obafluorin (5), β-lactone methanolysed obafluorin (6), β-lactone hydrolysed BA-obafluorin (7).

**Fig. 2:**
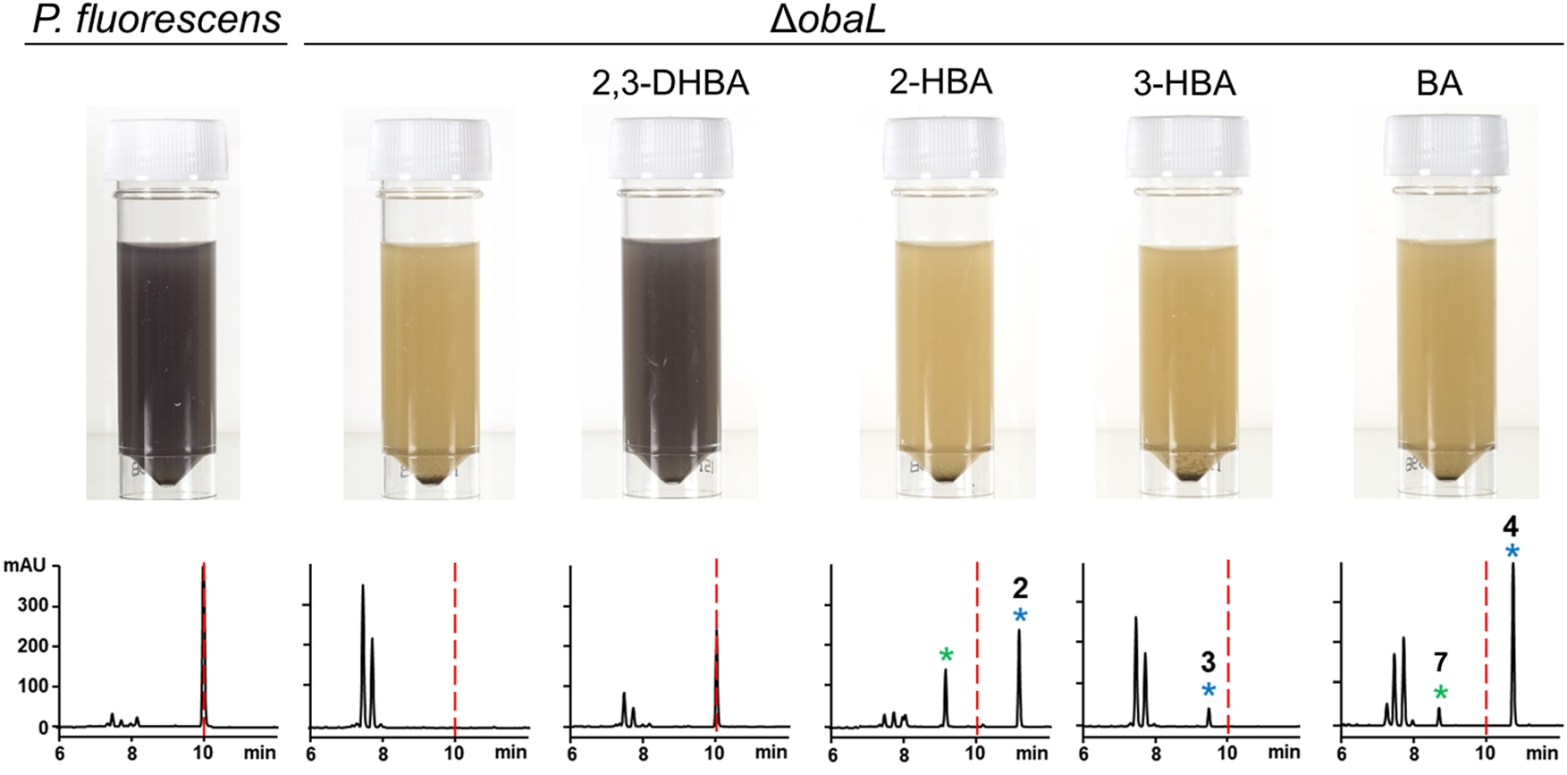
Production of novel 1 congeners with modified catechol moieties. *P. fluorescens* WT and Δ*obaL* cultures were grown ± 2,3-dihydroxybenzoic acid (2,3-DHBA, 0.2 mM), 2-hydroxybenzoic acid (2-HBA, 0.4 mM), 3-hydroxybenzoic acid (3-HBA, 0.4 mM) and benzoic acid (BA, 0.4 mM). Representative HPLC chromatograms at 270 nm of extracts taken after 14 h of growth are shown, with photographs of aliquots from the cultures. As shown previously,^24^ 2,3-DHBA restores 1 production to *P. fluorescens* Δ*obaL*, along with the characteristic purple colour. The retention time of 1 is indicated with a red dashed line. Peaks corresponding to novel 1 congeners are marked with a blue asterisk, with the numbers corresponding to Fig. 1. The peaks eluting between 7 and 8 minutes include the shunt metabolites 4-nitrophenylethanol and 4-nitrophenylacetate. For 2 and 4 the hydrolysed equivalents were also observed and are marked with a green asterisk. At least three biological replicates were carried out for each condition.

We hypothesised that variants of 2,3-DHBA might be processed through the 1 biosynthetic pathway to form analogues with modified catechol groups. To test this, we used the previously reported strain *P. fluorescens* Δ*obaL* that is deficient in 1 production, a phenotype that is rescued by the exogenous addition of 2,3-DHBA.^24^ We fed this strain with 2-hydroxybenzoic acid (2-HBA), 3-hydroxybenzoic acid (3-HBA) and benzoic acid (BA). Although none of the resultant cultures displayed the characteristic purple colour associated with 1 production, HPLC of extracts revealed several new peaks with retention times shifted relative to 1 (Fig. 2). Further analysis by LC-HRMS demonstrated that the new peaks displayed *m*/*z* values which corresponded to the expected masses of 2, 3 and 4 respectively (Fig. 1), along with the β-lactone hydrolysed equivalents eluting at earlier retention times for 2 and 4. Therefore, the 1 biosynthetic pathway can tolerate alternative 2,3-DHBA substrates to form analogues of 1 with modified catechol groups. These data agree with previous experiments that investigated the substrate promiscuity of ObaI (ObiF1) *in vitro*, in which the thioester intermediates of the phenylacetaldehyde analogues of 2–4 were detected in relatively high yield by LCMS.^26^

### Analogues of 1 are produced by *P. fluorescens* mutants in the absence of the immunity determinant ObaO

**Fig. 3:**
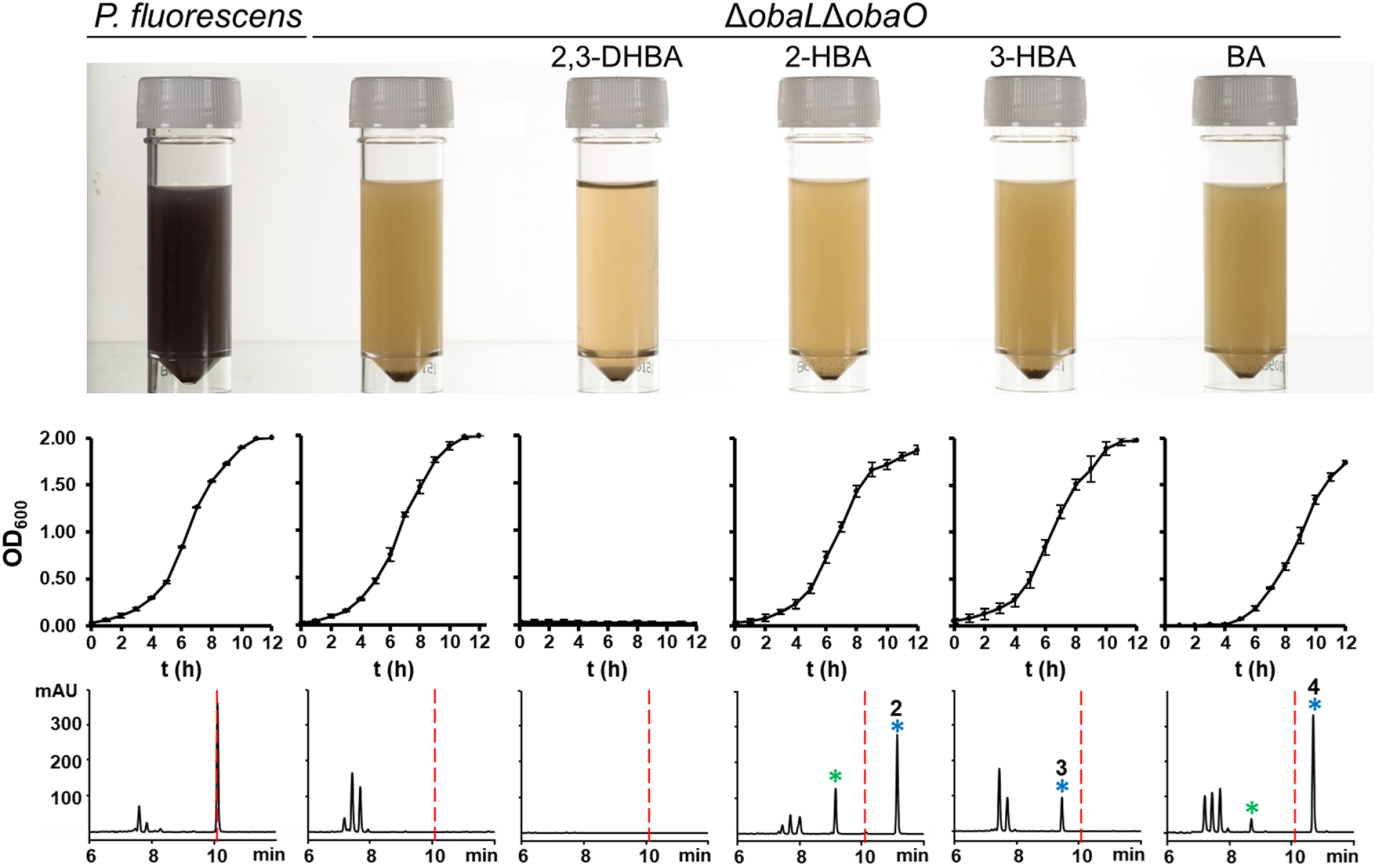
Analogues of 1 with modified catechol moieties are produced in the absence of the immunity determinant ObaO. *P. fluorescens* WT and Δ*obaL*Δ*obaO* cultures were grown ± 2,3-dihydroxybenzoic acid (2,3-DHBA, 0.2 mM), 2-hydroxybenzoic acid (2-HBA, 0.4 mM), 3-hydroxybenzoic acid (3-HBA, 0.4 mM) and benzoic acid (BA, 0.4 mM). As reported previously,^12^ the addition of 2,3-DHBA to *P. fluorescens* Δ*obaL*Δ*obaO* abolishes growth. In contrast, cultures fed with the modified benzoic acids grew normally and produced the expected analogues of 1. Top panel: aliquots of each strain after 14 h growth, with the purple coloration being indicative of 1 production. Middle panel: log phase growth curves, showing complete absence of growth for *P. fluorescens* Δ*obaL*Δ*obaO* + 2,3-DHBA only. Each data point is the average of three biological repeats, and bars show the standard error. Bottom panel: representative HPLC chromatograms at 270 nm of extracts for each condition at 14 h. 1 is indicated with a red dashed line and the peaks eluting between 7 and 8 minutes include the shunt metabolites 4-nitrophenylethanol and 4-nitrophenylacetate. Peaks corresponding to novel 1 congeners are marked with a blue asterisk, with the numbers corresponding to Fig. 1. For 2 and 4 the hydrolysed equivalents were also observed and are marked with a green asterisk.

Having demonstrated that 2, 3 and 4 could be produced *in vivo* we turned our attention to the effect of these congeners on the producing strain. *P. fluorescens* WT harbours an additional, resistant homologue of the ThrRS target, ObaO, to compensate for the action of 1 on the housekeeping enzyme PfThrRS. We previously showed that chemically complementing the Δ*obaL* mutation with 2,3-DHBA, in a Δ*obaO* background, completely abolished growth.^12^ In contrast, when *P. fluorescens* Δ*obaL*Δ*obaO* was fed with 2-HBA, 3-HBA or BA, we observed no significant impairment of growth and HPLC analysis demonstrated the formation of the peaks corresponding to 2, 3 and 4 respectively (Fig. 3). Therefore, modification of the catechol moiety enables growth and compound production in the absence of the immunity determinant ObaO.

### Purification and chemical characterisation of analogues 2–7

To better understand how the catechol moiety contributes to the bioactivity of 1 we proceeded to isolate 2, 3 and 4. *P. fluorescens* Δ*obaL* cultures were scaled up to 24 L and fed with 2-HBA, 3-HBA or BA. Crude extracts containing 2 and 3 were purified by reversed phase flash column chromatography, followed by preparative HPLC, to yield pure 2 (327 mg) and 3 (182 mg). The low solubility of 4 meant it could be readily precipitated from its crude extract by suspension in water/acetonitrile (1 : 1) to yield pure 4 (397 mg) without the need for further chromatography. The structures were confirmed by full 1D and 2D NMR characterisation (Fig. S20–S54, ESI†).

The strained β-lactone ring of 1 is readily hydrolysed at mildly basic pH or ring opens in the presence of nucleophiles. The intact β-lactone moiety was previously shown to be essential for bioactivity against *Bacillus licheniformis* SC9262.^10^ As we sought to investigate the role of the β-lactone in more detail, 1 was ring-opened by either hydrolysis with aqueous NaOH or methanolysis with MeOH according to previously published methods^10^ to yield the ring-open compounds 5 and 6 respectively. As an additional control for metal binding assays, we also chemically hydrolysed compound 4 using aqueous LiOH to yield the ring-open BA analogue, 7.

### Modification of the 1 catechol abolishes Fe^3+^ binding *in vitro*

Given the well-established function of catechol moieties in binding ferric iron, we assessed the ability of 1 and the novel analogues to bind iron. First, a colorimetric Chrome Azurol S (CAS) assay^35^ was employed, in which a colour change from blue to orange is indicative of Fe^3+^ binding. As expected, 1 showed strong ferric iron binding (Fig. S1, ESI†), in contrast to the analogues with the modified catechol. The colour change for 1 and the ring-open analogues 5 and 6 was much more pronounced than with 2,3-DHBA alone, consistent with the stabilisation of the *ortho*-phenolate by hydrogen bonding of the amide proton^36^ (Fig. S2, ESI†).

To directly assess iron binding, 1 was incubated with Fe^3+^ and/or a range of other biologically relevant metal ions and analysed using high resolution electrospray ionisation mass spectrometry (ESI-HRMS) (Table 1). Upon addition of Fe^3+^ a mass shift from 359 Da ([M + H]^+^) to 412 Da was observed, which corresponds to the [M − 2H + Fe^3+^]^+^ species. This indicates that ferric iron binds to 1*via* the deprotonated catechol, a motif often observed for catecholate siderophores.^36^ No mass shift was observed for Fe^2+^, Zn^2+^, Mg^2+^, or Ga^3+^. Addition of Mn^2+^ resulted in a peak consistent with a [M − H + Mn^2+^]^+^ species,^37^ but in contrast to incubation with Fe^3+^, the [M + H]^+^ peak was not fully depleted. Furthermore, in the presence of a mixture of all metal ions tested, only the [M − 2H + Fe^3+^]^+^ ion was observed, suggesting that the binding affinity of 1 for Fe^3+^ is greater than that of Mn^2+^ (Fig. S3, ESI†). On the other hand, no mass shift was observed when Fe^3+^ was added to compounds 2–4 and no depletion of the [M + H]^+^ species was observed (Fig. S4, ESI†). These combined results are consistent with 1 forming a strong, selective interaction with Fe^3+^ that is dependent on an intact catechol group.

**Table 1:** 1 forms a selective interaction with Fe^3+^ detectable by HRMS. Binding of 1 to a selection of biologically relevant metals was monitored by ESI-HRMS on a Synapt G2-Simass spectrometer. 1 bound strongly and selectively to Fe^3+^, with some weaker binding to Mn^2+^

Adduct	MS peak (*m*/*z*)	Species	Error (ppm)
1	359.0872	[M + H]^+^	−0.6
1 + Fe^2+^	359.0879	[M + H]^+^	1.4
1 + Fe^3+^	411.9980	[M − 2H + Fe^3+^]^+^	−1.9
1 + Mn^2+^	412.0089	[M − H + Mn^2+^]^+^	−2.2
1 + Zn^2+^	359.0867	[M + H]^+^	−1.9
1 + Mg^2+^	359.0869	[M + H]^+^	−1.4
1 + Ga^3+^	359.0881	[M + H]^+^	1.5
1 + mix	411.9992	[M − 2H + Fe^3+^]^+^	−1.0

To further probe the interaction of 1 with Fe^3+^ ions, and to confirm the 1 : 1 stoichiometry observed in the MS experiments, we utilised Job's method of continuous variation.^38^ An absorbance maximum was observed at a mole fraction of 0.5 indicative of 1 : 1 complex with Fe^3+^, consistent with the salicylic acid (2-HBA) positive control (Fig. S5, ESI†).

Given the EcThrRS-1 crystal structure showing the catechol coordinating the essential Zn^2+^ ion in the ThrRS active site published concurrently with our work,^34^ we were surprised to have observed the complete absence of a mass shift or depletion of the [M + H]^+^ peak upon addition of Zn^2+^. Reasoning that this could result in a neutral species that would not fly in MS, we also tested Zn^2+^ binding with a colorimetric 4-(2-pyridylazo)-resorcinol (PAR) assay. Zn^2+^ binding is observed as a colour change from orange to yellow, resulting from a decrease in Zn(PAR)_2_ at 495 nm and increase in free PAR at 410 nm.^39^ Only at the highest molar ratio of 25 : 1 compound : Zn^2+^ was a modest decrease in the Zn(PAR)_2_ peak and slight increase in the free PAR peak observed for 1, but not 2–4, suggestive of some weak Zn^2+^ binding *via* the catechol. In contrast, the *N*,*N*,*N*′,*N*′-tetrakis(2-pyridinylmethyl)-1,2-ethanediamine (TPEN) Zn^2+^ binding positive control completely displaced PAR at molar ratios of >1 (Fig. S6 and S7, ESI†).

### The catechol moiety of 1 is essential for antibacterial activity and inhibition of ThrRS

With compounds 2–7 in hand, we went on to test their activity against a range of bioindicator strains. We previously established a spot-on-lawn method for 1 bioassays, given that 1 reacts with both filter disks and media components in microbroth dilution assays.^12^ As reported previously,^12^1 was most active against Gram-positive strains, with minimum inhibitory concentrations (MICs) of 2 μg mL^−1^ and 4 μg mL^−1^ against MRSA and *B. subtilis*, respectively (Table 2 and Fig. S8–S12, ESI†). Whilst the activity was reduced against the *E. coli* ATCC 25922 bioindicator strain to 256 μg mL^−1^, the MIC with the *E. coli* strain NR698 which has a deficient outer membrane (effectively mimicking a Gram-positive membrane) was 4 μg mL^−1^; this suggests that uptake is the main barrier to activity against Gram-negative organisms. *Saccharomyces cerevisiae* was included as a representative eukaryotic strain, against which 1 showed activity only at the greatest concentration tested of 1000 μg mL^−1^.

**Table 2:** The antibacterial activity of 1 is dependent on the catechol moiety. Minimum inhibitory concentrations (MICs) of 1 and analogues against a range of bioindicator strains determined by spot-on-lawn bioassays

Compound	MIC (μg mL^−1^)				
	MRSA	*B. subtilis*	*E. coli* 25922	*E. coli* NR698	*S. cerevisiae*
1	2	4	256	4	1000
2	>1000	1000	>1000	1000	>1000
3	1000	1000	>1000	>1000	>1000
4	1000	>1000	>1000	>1000	>1000

In contrast, the modified catechol analogues 2, 3 and 4, showed little or no activity against all the strains tested. This clearly demonstrates that the intact catechol is essential for bioactivity. The ring-open analogues 5, 6 and 7 were also inactive as anticipated (Fig. S13, ESI†).

**Fig. 4:**
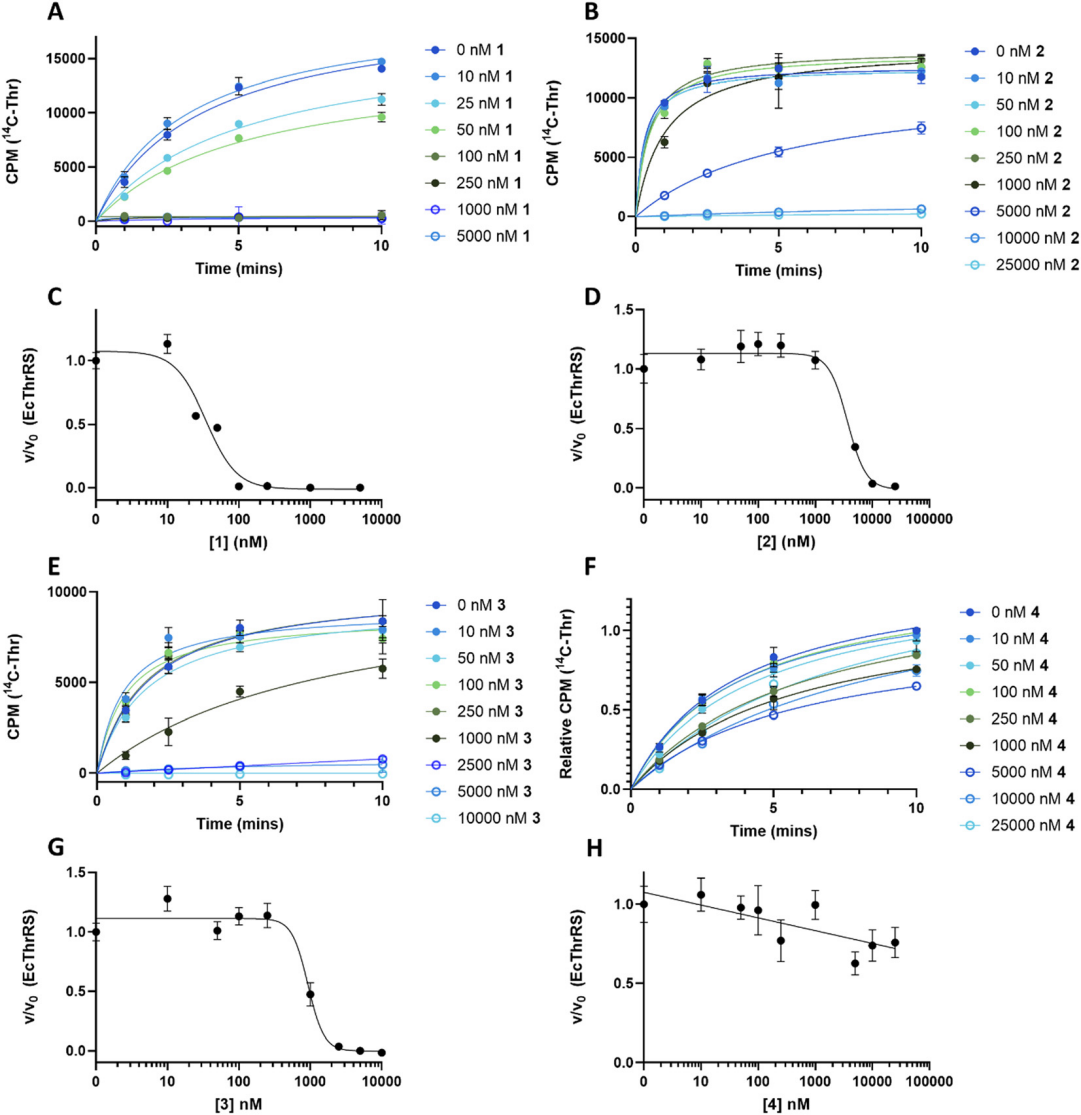
The catechol group of 1 is essential for inhibition of EcThrRS. Progress curves for EcThrRS in the presence of varying (0–25 μM) concentrations of 1 (A), 2 (B), 3 (E) and 4 (F). Reactions (*n* = 3) included enzyme at 10 nM, which was preincubated with compound for 10 min prior to the addition of saturating concentrations of tRNA, threonine, and ATP. Inhibition of EcThrRS by 2 and 3 was greatly reduced relative to 1, and 4 showed no significant inhibition. The linear portions of the progress curves were fit to a linear equation to derive initial rates. Error bars represent the standard error for each time point. Units given for y axis in (F) are relative CPM to provide consistency and accounting for differences in different scintillation counters used to measure CPM for different concentrations of 4. Dose response curves for EcThrRS with compound 1 (C), 2 (D), 3 (G) and 4 (H) were calculated from the data in (A), (B), (E) and (F) respectively, by plotting the fractional velocity (v/v_0_) at each measured inhibitor concentration against log [compound]. IC_50_ values were: 35 ± 4 nM for 1, 3700 ± 700 nM for 2 and 930 ± 80 nM for 3 and no inhibition for 4. Details of the fitting routines are presented in the Methods. CPM = counts per min.

**Fig. 5:**
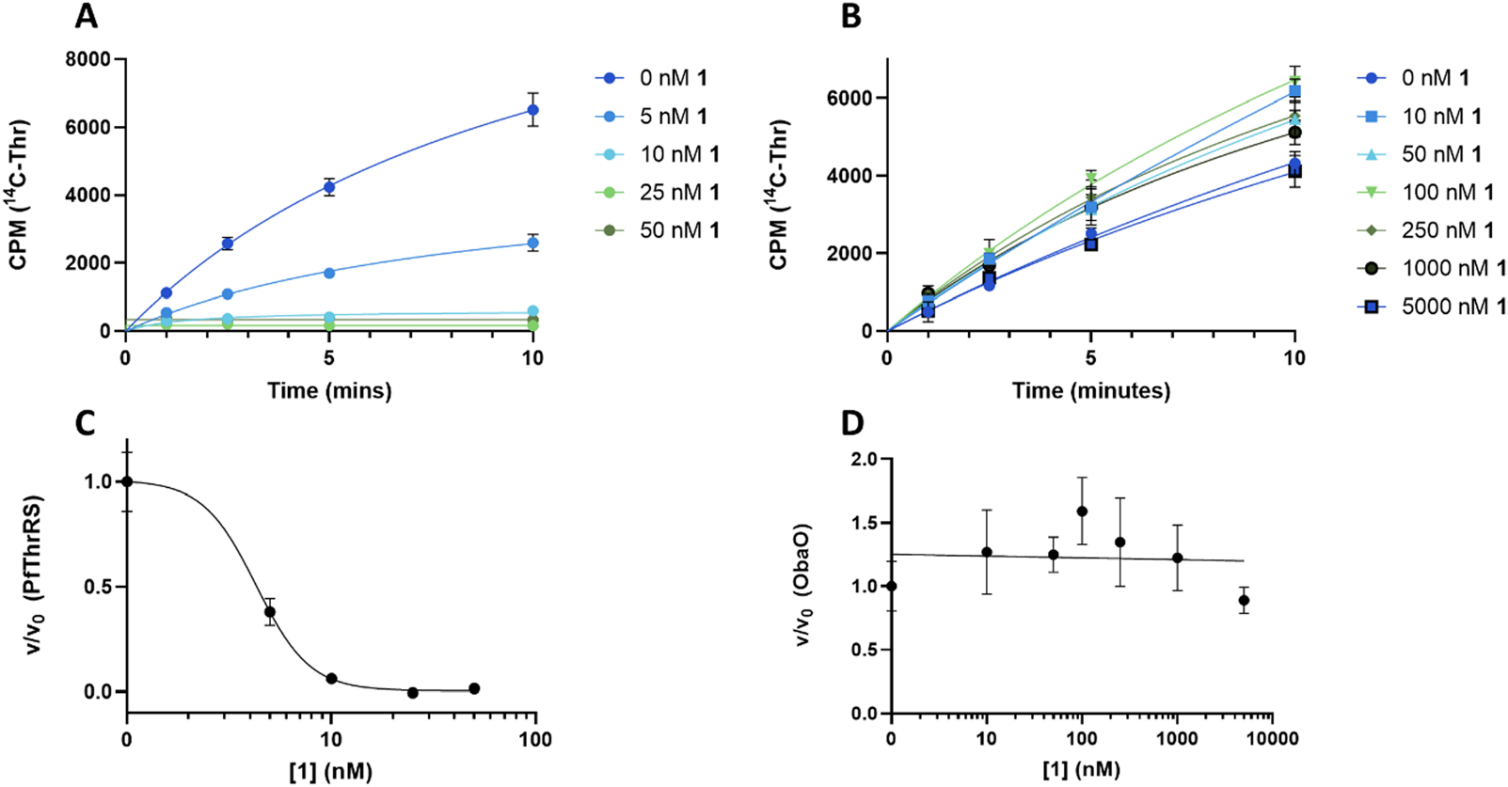
ObaO is the sole 1-resistant ThrRS in *P. fluorescens*. Progress curves for PfThrRS (A) and ObaO (B) in the presence of varying (0–5 μM) concentrations of 1. Reactions (*n* = 3) included enzyme at 10 nM, which was preincubated with compound for 10 min prior to the addition of saturating concentrations of tRNA, threonine, and ATP. Concentration ranges of 1 were adjusted according to the inhibition profiles. 1 was a potent inhibitor of PfThrRS, with full inhibition at 10 nM, whereas there was no significant inhibition of ObaO up to 5000 nM, demonstrating the latter serves as the sole 1-resistant ThrRS in the strain. The linear portions of the progress curves were fit to a linear equation to derive initial rates. Error bars represent the standard error for each time point. Dose response curves for PfThrRS (C) and ObaO (D) were calculated from the data in (A) and (B) respectively by plotting the fractional velocity (v/v_0_) at each of the tested inhibitor concentrations against log [1]. The IC_50_ value for 1 and PfThrRS is 4.3 ± 0.4 nM, while ObaO was not inhibited by 1. Details of the fitting routines are presented in the Methods. CPM = counts per min.

To understand the effect of analogues 2–4 on the target enzyme ThrRS, *in vitro* aminoacylation assays were performed. We monitored the formation of Thr-tRNA^Thr^ by EcThrRS in the presence of 0–5 μM 1 or analogue. For 1, the rate of aminoacylation of tRNA decreased rapidly over this concentration range, with complete inhibition occurring around 100 nM. Fitting of the data to a dose response equation returned an IC_50_ of 35 ± 4 nM (Fig. 4A and C). Noting that this value is lower than we reported previously,^12^ we reassessed the preparation of 1 used in the original aminoacylation assays. We determined that this contained impurities not detected by HPLC-UV, whereas the 1 used here was >95% pure by HPLC-UV, HRMS, ELSD and NMR. This establishes 1 as a more potent ThrRS inhibitor than previously thought. With this in mind, the formation of Thr-tRNA^Thr^ by ObaO was also monitored in the presence of 0–5 μM 1, resulting in no observable inhibition by 1 at any concentration. The previously observed partial inhibition of ObaO was likely due to the undetected impurities in the previous 1 preparation (Fig. 5B and D).

In contrast to 1, analogues 2–4 had little effect on aminoacylation of EcThrRS. Complete inhibition was only observed at 10 000 nM for 2 and 5000 nM for 3, whilst for 4 there was no significant inhibition of EcThrRS even at the highest concentration tested. Corresponding IC_50_ values were 3700 ± 700 nM for 2 and 930 ± 80 nM for 3, demonstrating the analogues are approximately 100- and 30-fold less potent than 1, respectively (Fig. 4). We also assessed the effect of 1 on the *P. fluorescens* housekeeping ThrRS, PfThrRS (Fig. 5A and C). As suggested by our *P. fluorescens* Δ*obaL*Δ*obaO* feeding experiments, above (Fig. 3), and previously,^12^1 is also a potent inhibitor of PfThrRS, with an IC_50_ of 4.3 ± 0.4 nM, confirming that ObaO functions as the sole 1-resistant ThrRS in the strain. In contrast, analogues 2–4 had little effect on aminoacylation by PfThrRS, mirroring the *in vivo* results (Fig. S14, ESI†).

These combined data (Table 3) clearly demonstrate that the catechol moiety of 1 is essential for the antibacterial activity *via* direct interaction with the ThrRS target.

**Table 3:** Inhibition of 1-sensitive ThrRSs requires an intact catechol group. Half maximal inhibitory concentrations (IC_50_s) for each compound with each of the ThrRSs tested in this study

Compound	Protein	IC_50_ (nM)
1	EcThrRS	35 ± 4
	PfThrRS	4.3 ± 0.4
	ObaO	No inhibition

2	EcThrRS	3700 ± 700
	PfThrRS	1600 ± 300

3	EcThrRS	930 ± 80
	PfThrRS	2500 ± 800

4	EcThrRS	No inhibition
	PfThrRS	No inhibition

### Increasing concentrations of Fe^3+^ leads to a decrease in the MIC of 1

Our combined bioassay and biochemical data confirmed that the 1 catechol is essential for its inhibitory activity, consistent with the recent observation that this moiety coordinates Zn^2+^ in the ThrRS active site.^34^ Given the additional observation that, in solution, 1 is a far stronger Fe^3+^-binder than Zn^2+^-binder, we wondered what role iron binding might play in the mode of action. Specifically, we hypothesised that iron-binding might facilitate 1 uptake in Gram-negative organisms, with the catechol playing a dual role in Fe-mediated uptake as well as the direct Zn^2+^ interaction with the target. Conjugation of an active compound to a siderophore moiety is a strategy employed by THAs to hijack the iron update machinery of a competing bacterium and facilitate transport of active component the across the Gram-negative membrane.^32^

To explore this hypothesis, we first examined the antibacterial activity of 1 against the Gram-negative strains *E. coli* 25922 and *P. aeruginosa* PA01 as described above, but with increasing concentrations of Fe^3+^ ions present in the growth agar. If 1 acts as a THA then we should observe similar or higher MICs at elevated concentrations of iron as the organisms switch off their active uptake mechanisms. In contrast, and to our surprise, we observed a Fe^3+^ concentration dependent decrease in MIC against both strains (Table 4), with an ≥256-fold decrease in MIC for *E. coli* ATCC 25922 and a ≥128-fold decrease for *P. aeruginosa* PA01 (Fig. S15, ESI†). We also examined the effect of Fe^3+^ on the 1 MIC with MRSA as a representative Gram-positive organism and observed an 8-fold decrease in MIC (Fig. S16, ESI†).

**Table 4:** Minimum inhibitory concentrations (MICs) of 1 against *E. coli* 25922, *P. aeruginosa* PA01 and MRSA. Determined from spot-on-lawn bioassays performed with and without Fe^3+^ added to the media

Organism	MIC of 1/μg mL^−1^	
		+Fe^3+^
*E. coli* 25922	256	≤1
*P. aeruginosa* PA01	128	≤1
MRSA	2	0.25

This antibacterial assay was then repeated with *E. coli* ATCC 25 922 but under iron depleted conditions through addition of the Fe^3+^ chelator 2,2′-bipyridyl (bipy; 150 μM) to the growth agar. For THAs a decrease in MIC is anticipated upon iron depletion as the target strain is expected to actively transport the THA-Fe^3+^ complex into the cell.^40,41^ However, no change in the MIC was observed when compared to normal assay conditions, consistent with the results of the assay under iron replete conditions above and the conclusion that 1 does not act as a THA (Table 5).

**Table 5:** 1 does not act as a THA. Minimum inhibitory concentrations (MICs) of *E. coli* 25 922, BW25113 and siderophore TonB dependent transporter (TBDT) knock-out mutants, Δ3 and Δ6; Δ3 = Δ*fhu*AΔ*fecA*Δ*cirA* and Δ6 = Δ*fhuA*Δ*fecA*Δ*cirA*Δ*fepA*Δ*fhuE*Δ*fiu*. Determined from spot-on-lawn bioassays under standard, iron depleted (+150 μM bipy) and iron replete (+2 mM Fe^3+^) conditions. Note the strain Δ6 is unable to grow under iron depleted conditions

Organism	1 MIC/μg mL^−1^		
		+bipy (150 μM)	+Fe^3+^ (2 mM)
*E. coli* ATCC 25922	256	256	≤1
*E. coli* BW25113	256	256	≤1
*E. coli* BW25113 Δ3	256	256	≤1
*E. coli* BW25113 Δ6	256	No growth	≤1

To gain further support for our conclusions, we repeated this experiment but grew *E. coli* ATCC 25 922 alongside *E. coli* BW25113 mutants in which different combinations of the six siderophore TonB-dependent transporters (TBDTs) FhuA, FecA, CirA, FepA, FhuE and Fiu have been sequentially deleted.^42^ It has been shown that the TBDTs CirA and Fiu are responsible for the uptake of catecholate containing compounds,^43^ including THAs.^40^ Therefore, if 1 acts as a THA we would expect mutants *E. coli* BW25113 Δ*fhuA*Δ*fecA*Δ*cirA* (Δ*3*) and/or Δ*fhuA*Δ*fecA*Δ*cirA*Δ*fepA*Δ*fhuE*Δ*fiu* (Δ*6*) to have increased 1 MICs when grown under iron depleted conditions. When grown in the presence of bipy (150 μM) Δ6 did not grow at all, consistent with a complete lack of iron uptake, whereas mutant Δ3 grew normally and the 1 MIC was unchanged in the presence of bipy. Furthermore, as for *E. coli* 25922, the MIC of 1 against *E. coli* BW25113 WT, Δ3 and Δ6 decreased ≥256-fold upon addition of Fe^3+^ (Table 5 and Fig. S17, ESI†). These combined data are consistent with the conclusion that 1 does not function as a THA.

To control for any general effects of increased Fe^3+^ concentrations on antibiosis we tested the effect of increased levels of Fe^3+^ (2 mM) on the antibacterial activity against *E. coli* 25922 and *S. aureus* of several antibiotics which operate through a range of mechanisms. Whilst we observed some impact on MIC, overall, the addition of Fe^3+^ tended to increase rather than decrease MIC values, and effects were much less marked than for 1 (Table S1, ESI†). This suggests that the observations above are specific to 1.

### Iron-binding inhibits the hydrolytic breakdown of 1

As 1 does not act as a THA, we were intrigued as to the role that interaction with Fe^3+^ may have in its increased antibacterial activity. As noted, the MIC of 1 against *E. coli* 25922 and *P. aeruginosa* PA01 in the presence of ferric iron is similar to that observed for Gram-positive organisms or *E. coli* NR698 which has a deficient outer membrane. This suggested that binding to Fe^3+^ may enable passive uptake. However, the surprising ≥128–256 fold increase in activity and the decrease in MIC for Gram-positive organisms suggested there may be an additional component to this observation. With this in mind, we were drawn to empirical observations made during previous work to increase titres of 1 during fermentation, where we noticed that increased levels of iron in production media reliably correlated with an increased proportion of 1 compared to the hydrolysed β-lactone congener 5; this suggested that the presence of Fe^3+^ was affecting the rate of 1 hydrolysis, potentially conferring a protective effect.

**Fig. 6:**
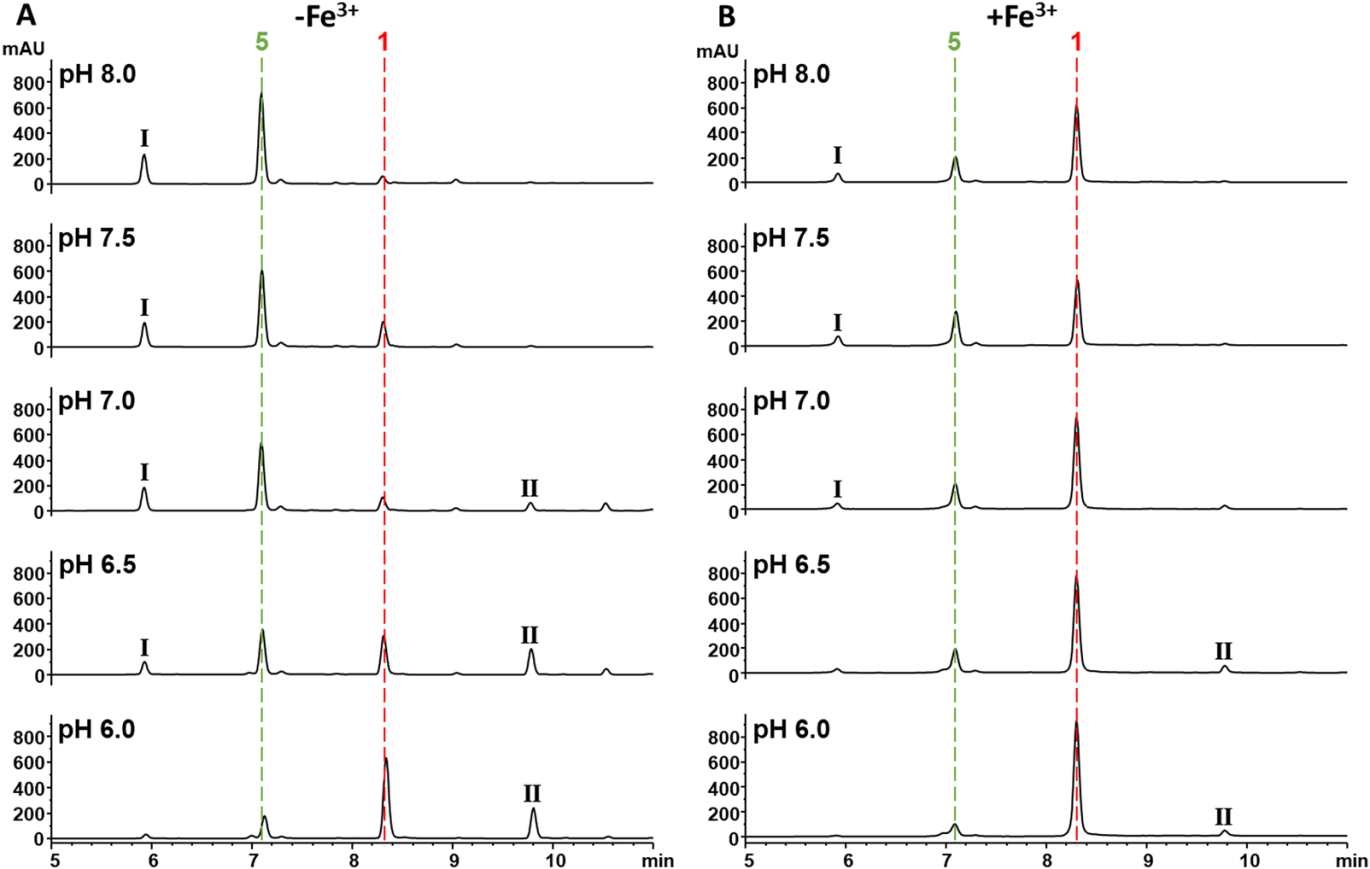
Fe^3+^ binding *via* the catechol protects the β-lactone of 1 from hydrolysis. (A) UV chromatograms at 270 nm of solutions of 1 (1 mM) incubated in HEPES buffer (100 mM) pH 6.0–8.0. The red and green dashed lines represent 1 and 5 respectively. The peaks labelled I and II are tentatively identified as a 1-HEPES congener and an obafluorin dimer respectively by HRMS (Fig. S19, ESI†). (B) UV chromatograms at 270 nm of solutions of 1 (1 mM) incubated in HEPES buffer (100 mM) pH 6.0–8.0 with 1 mM Fe^3+^. The red and green dashed lines represent 1 and 5 respectively. The peaks labelled I and II are tentatively identified as a 1-HEPES adduct and an obafluorin dimer respectively by HRMS (Fig. S19, ESI†).

To investigate this possibility, the hydrolysis of 1 was monitored by UV (270 nm) and MS after incubation for 30 min at half unit intervals between pH 6.0 and 8.0. We observed pH-dependent 1 hydrolysis, with little change observed at pH 6.0, but with the majority of 1 in the hydrolysed form at pH 8.0 (Fig. 6A). To test the protective effects of Fe^3+^ against hydrolysis, we then repeated this experiment but with added Fe^3+^ (1 mM) and found that this protected 1 against hydrolysis at all pH values (Fig. 6B). To determine what role the catechol group plays in this, hydrolysis of analogues 2–4 at pH 6.0 and 8.0 was subsequently investigated. In common with 1, compounds 2 and 3 were mainly hydrolysed at pH 8.0 *vs.* pH 6.0, whereas there was no hydrolysis of compound 4 at pH 8.0 (Fig. S18, ESI†). This is consistent with hydrolysis of the 1 β-lactone being facilitated by intramolecular catalysis *via* the catechol hydroxyls, as suggested previously.^11^ In contrast to 1, addition of Fe^3+^ had no effect on the hydrolysis of 2 and 3. Together these data demonstrate Fe^3+^ inhibits the hydrolysis of the 1 β-lactone by binding the catechol group and likely preventing intramolecular-catalysed β-lactone hydrolysis.

## Conclusions

In this study we investigated 1, an antibiotic discovered almost 40 years ago.^9,10^ We recently showed that 1 inhibits bacterial growth through the inhibition of ThrRS in sensitive organisms, a target that is distinct from any clinically utilised antibiotic.^5^ The results here shed further light on the mechanism by which 1 inhibits ThrRS by identifying the catechol moiety as an essential molecular feature required for antibiosis and the inhibition of ThrRS. This data is consistent with work published during the preparation of this manuscript which described a crystal structure of 1 bound to ThrRS from *E. coli*, and which showed that the phenol groups of the catechol form key interactions with the essential Zn^2+^ ion present in the active site.^34^ Our results further show that 1 is a weak binder of Zn^2+^ in solution, suggesting that the Zn^2+^ interaction is specific to the ThrRS active site and that the 1 mode of action does not involve the modulation of zinc homeostasis as do dithiolopyrrolone antibiotics.^44,45^

Additionally, our results show that 1 readily forms a 1 : 1 complex with Fe^3+^ ions and that this association protects 1 from hydrolysis in solution. This is important as an intact β-lactone moiety is required for the antibacterial activity. Moreover, the presence of ferric iron increases the antibacterial activity of 1 against *E. coli* and *P. aeruginosa*, with the MIC decreasing by over two orders of magnitude in spot-on-lawn bioassays. This decrease is dramatic, and these MICs are in line with those observed without the addition of ferric iron for Gram-positive organisms and for *E. coli* NR698 which has a compromised outer membrane. Taken together these observations suggest that, in addition to protecting 1 from hydrolysis, and thereby maintaining effective concentrations of the active species, the association with Fe^3+^ ions may increase transport across the Gram-negative cell outer membrane. Our work ruled out the likelihood that 1 operates as a THA, suggesting that increased transport is due to a passive mechanism. While the presence of iron is important for the activity of some antibiotics such as streptonigrin,^46^ or the ability of siderophores to scavenge iron leading to antimicrobial activity,^27^ the multiple roles of the 1 catechol moiety in direct target interaction, compound stability and potentially uptake is without precedent. However, how these effects play out under iron limited physiological conditions remains to be determined.

**Fig. 7:**
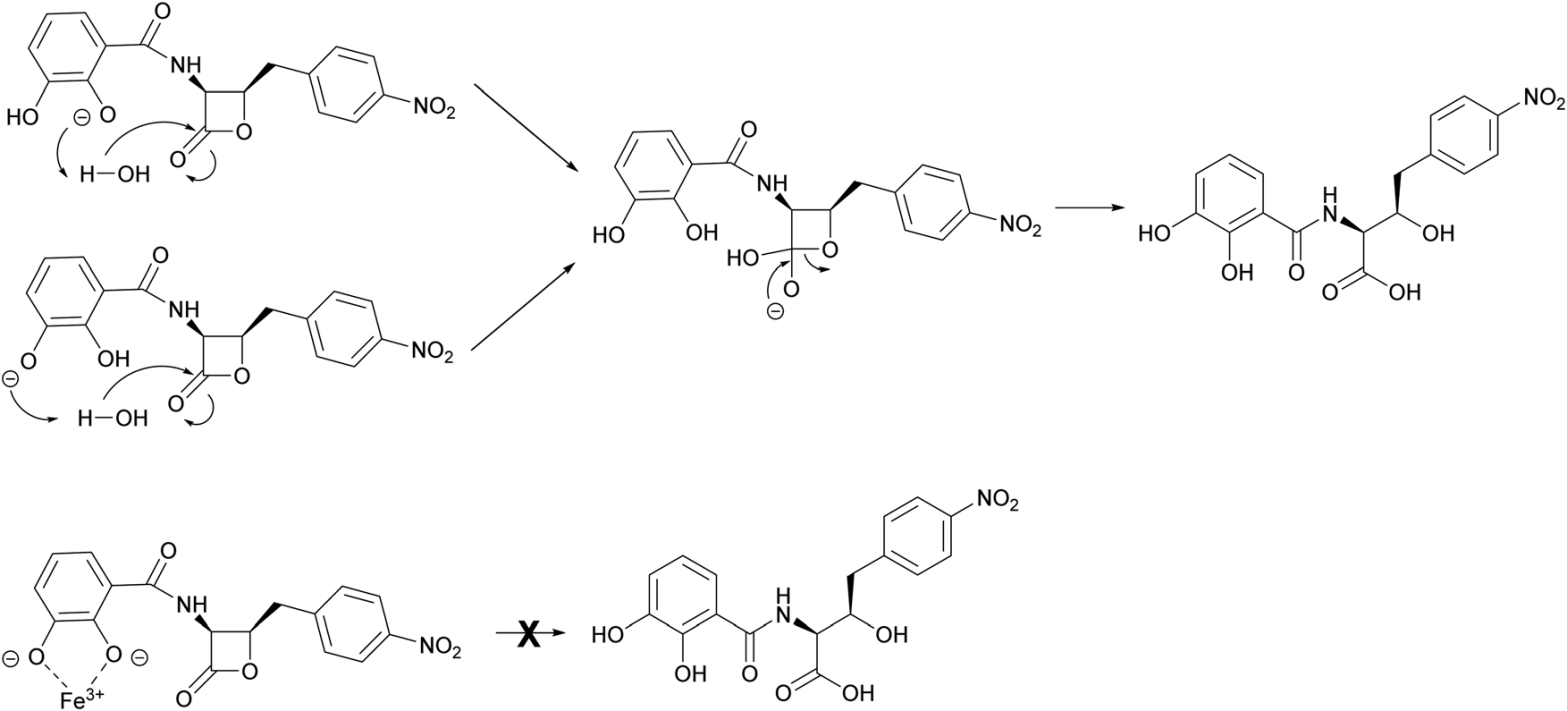
Proposed mechanism of Fe^3+^-mediated protection of 1 from hydrolysis. In the absence of Fe^3+^, the phenol groups of the catechol moiety are able to enhance the attack of water on the β-lactone carbonyl. In the presence of Fe^3+^, the deprotonated catecholate coordinates the Fe^3+^ ion, effectively preventing hydrolytic breakdown of the lactone.

The mechanism by which interaction with ferric iron protects 1 from hydrolysis is not fully understood. However, it is likely to involve the phenolic groups of the catechol moiety which become deprotonated upon binding to Fe^3+^. This is consistent with loss of the protective effect for analogues 2 and 3, whereas the benzoic acid analogue 4 is stable to hydrolysis at pH 8 (Fig. S18, ESI†). This phenomenon was noted during the chemical synthesis of 1 and 4 by Pu *et al.*,^11^ who suggested that hydrolytic breakdown of 1 could be catalysed by basic impurities present in samples, aided by a catecholate anion that enhances the attack by water to form a tetrahedral intermediate (Fig. 7). The observations reported here are important for the future design of 1 analogues with improved antibacterial activity and biophysical properties. Moreover, our results indicate that the ability of 1 to bind ferrous iron impacts its chemical and biological properties, a factor that can be exploited in future preclinical investigations.

## Material and methods

### General

Solvents used for extractions and HPLC analysis were bought from Fisher Scientific. Reagents and chemicals were purchased from Alfa-Aesar and Sigma-Aldrich (Merck). ^14^C labeled l-threonine [^14^C(U)] was purchased from Moravek Inc. ‘Microtitre plate’ refers to Greiner bio-one, flat bottom 96 well suspension culture plates.

### Bacterial strains

All strains were maintained on solid LB medium with appropriate selection. Growth temperatures were 28 °C for *P. fluorescens*, 30 °C for *S. cerevisiae* and 37 °C for *E. coli*, *B. subtilis* and *S. aureus*.

**Table:** 

Strain	Description	Ref.
*P. fluorescens* ATCC 39502	1-Producing strain, WT	ATCC, USA
*P. fluorescens* Δ*obaL*	ATCC 39502 with an in-frame truncation in the *obaL* gene	Scott *et al.*, 2017^24^
*P. fluorescens* Δ*obaL*Δ*obaO*	ATCC 39502 with an in-frame truncation in the *obaL* and *obaO* genes	Scott *et al.*, 2019^12^
Methicillin-resistant *Staphylococcus aureus* (MRSA)	Bioassay strain; Clinical isolate provided by Dr Justin O’Grady (UEA Medical School)	Qin *et al.*, 2017^47^
*B. subtilis* EC 1524	Bioassay strain; *trpC2*, Subtilin BGC deleted	O’Rourke *et al.*, 2017^48^
*E. coli* ATCC 25922	Bioassay strain; WT	ATCC, USA
*E. coli* NR698	Bioassay strain; MC4100 (F^−^ araD139 Δ(argF-lac)U169, rpsL150, relA1, flbB5301, deoC1, ptsF25, rbsR), imp4213	Ruiz *et al.*, 2005^49^
*S. cerevisiae* NCYC 2939	Bioassay strain; MUCL 39234, S288C, ATCC 26108; MATalpha sta1 sta2 sta3 STA10	NCYC
*E. coli* BW25113	Bioassay strain; WT	Grinter *et al.*, 2019^42^
*E. coli* BW25113 Δ3	Bioassay strain deficient in TBDTs: Δ*fhuA*Δ*fecA*Δ*cirA*	Grinter *et al.*, 2019^42^
*E. coli* BW25113 Δ6	Bioassay strain deficient in TBDTs: Δ*fhuA*Δ*fecA*Δ*cirA*Δ*fepA*Δ*fhuE*Δ*fiu*	Grinter *et al.*, 2019^42^
*E. coli* DH5α	Cloning strain; F *endA1 ginV44 thi-1 recA1 relA1 gyrA96 deoR nupG ϕ80dlacΔ(lacZ)M15* Δ*(lacIZYA-argF)*U169 *hsdR17*(r_K_^−^m_K_^+^) *λ*-	Lab Stock
*E. coli* NiCo21 (DE3)	Protein expression strain; BL21(DE3) derivative *can::CBD fhuA2 [lon] ompT gal* (*λ* DE3) *[dcm] arnA::CBD slyD::CBD glmS6Ala ΔhsdS λ* DE3 = *λ sBamHlo* Δ*EcoRI-B int::(lacl::PlacUV5::T7 gene1) is21 Δnin5*	New England Biolabs (NEB)
*E. coli* NiCo21 (DE3) pET28a(+)-*EcThrRS*	NiCo21 (DE3) carrying the pET28a(+)-*EcThrRS* plasmid for the production of His_6_-EcThrRS protein	Scott *et al.*, 2019^12^
*E. coli* NiCo21 (DE3) pET28a(+)-*obaO*	NiCo21 (DE3) carrying the pET28a(+)-*obaO* plasmid for the production of His_6_-ObaO protein	Scott *et al.*, 2019^12^
*E. coli* NiCo21 (DE3) pET28a(+)-*PfThrRS*	NiCo21 (DE3) carrying the pET28a(+)-*PfThrRS* plasmid for the production of His_6_-PfThrRS protein	This work
*E. coli* MRE600:pWFW1015	tRNA^Thr^ Overexpression strain; *E. coli* MRE600 containing the pWFW1015 plasmid for overexpression of the tRNA^Thr^ encoded by thrW	Waas and Schimmel, 2007^50^

### Analysis of *P. fluorescens* growth and 1/analogue production

Wild-type and mutant *P. fluorescens* ATCC 39502 strains were maintained on LB agar plates supplemented with nitrofurantoin (100 μg mL^−1^). Strains were grown in 1 production medium (OPM), comprising: yeast extract 0.5%, d-glucose 0.5%, MgSO_4_·7H_2_O 0.01%, and FeSO_4_ 0.01%, dissolved in Milli-Q (Merck Millipore) filtered water. A toothpick was used to inoculate 100 mL of OPM starter culture in a 250 mL Erlenmeyer flask from a single colony, with subsequent growth for 24 h at 25 °C, 300 rpm. 1 mL of this culture was used to inoculate 100 mL OPM production cultures in a 500 mL Erlenmeyer flask. Where required, production cultures were supplemented with: 2,3-DHBA (0.2 mM final concentration), 2-HBA (0.4 mM final concentration), 3-HBA (0.4 mM final concentration) or BA (0.4 mM final concentration) in DMSO. For growth curves, production cultures were grown at 25 °C, 300 rpm and OD_600_ measurements relative to an OPM blank were recorded every hour for 12 h, in biological triplicate. For metabolite analysis, after 14 h of growth, 1 mL of culture broth was extracted with an equal volume of ethyl acetate by mixing at 1400 rpm for 15 min. Samples were then centrifuged (15 682 × *g* for 15 min), and the organic phase was collected and evaporated. The resulting organic extract was dissolved in acetonitrile (ACN) (250 μL) and centrifuged (15 682 × *g* for 20 min) to remove any remaining cell debris, before HPLC analyses. For photographs, a 20 mL aliquot of the culture at 14 h was transferred to a screw-cap vial.

### Analytical HPLC and LCMS

Extracts from *P. fluorescens* cultures were analysed on an Agilent 1100 system using a Gemini 3 μm NX-C_18_ 110 Å, 150 × 4.6 mm column (Phenomenex) with a gradient elution: ACN/0.1% (v/v) TFA (H_2_O) gradient from 10/90 to 100/0 0–15 min, 100/0 for 15–16 min, gradient to 10/90 16–16.50 min and 10/90 for 16.50–23 min. The flow rate was 1 mL min^−1^ and the injection volume of each sample 10 μL. DAD signals were analysed at 270 nm, with a bandwidth of 4 nm.

Hydrolysis of 1–4 was analysed on an Agilent 1290 system fitted with an Agilent LC/MSD MS spectrometer. A Kinetex XB-C_18_, 100 × 4.6 mm, 5 μM column (Phenomenex) was used with a gradient elution: ACN/0.1% (v/v) FA (H_2_O) gradient from 5/95 for 0–0.5 min, 5/95 to 98/2 0.5–12 min, 98/2 for 12–13 min, gradient to 5/95 13–13.5 min and 5/95 for 13.5–15 min. The flow rate was 0.6 mL min^−1^ and the injection volume of each sample 5 μL. DAD signals were analysed at 270 nm, with a bandwidth of 4 nm. MS were recorded in positive mode using an atmospheric pressure ionisation electrospray (API-ES) ion source with a capillary voltage of 3000 V. The drying gas temperature was 350 °C with a flow rate of 12 L min^−1^. The scan parameters were: mass range = 200–1500; fragmentor = 70; gain EMV = 1.0; threshold = 150; step-size = 0.10. Data were analysed with Agilent ChemStation software.

LC-HRMS of side products of hydrolysis I–VI (Fig. S19, ESI†) were recorded using an Agilent 1290 system fitted with an Agilent Q-ToF mass spectrometer. A Kinetex XB-C_18_, 100 × 4.6 mm, 5 μM column (Phenomenex) was used with a gradient elution: ACN/0.1% (v/v) FA (H_2_O) gradient from 5/95 for 0–0.5 min, 5/95 to 98/2 0.5–12 min, 98/2 for 12–13 min, gradient to 5/95 13–13.5 min and 5/95 for 13.5–15 min. The flow rate was 0.6 mL min^−1^ and the injection volume of each sample 5 μL. DAD signals were acquired at 270, with a bandwidth of 4 nm. HRMS were recorded in positive mode using a dual Agilent Jet Stream (AJS) ESI ion source with a capillary voltage of 3500 V and a nozzle voltage of 1000 V. The drying gas temperature was 320 °C with a flow of 8 L min^−1^, and the sheath gas temperature was 350 °C with a sheath gas flow of 11 L min^−1^. Reference mass correction was enabled with monitoring of reference masses with *m*/*z* 121.050873 and 922.009798. Data were analysed with Agilent MassHunter software.

### Compound purification and characterisation

#### Instruments

Preparative HPLC was performed on a Dionex UltiMate 3000 HPLC system using a Phenomenex Gemini 5 μm C_18_ 110 Å 150 × 21.2 mm column with gradient elution: ACN/H_2_O 5/95 for 0–2 min, gradient to 45/55 2–4 min, then gradient to 80/20 4–12 min, 80/20 for 12–14 min, gradient to 5/95 14–15 min. The flow rate was 20 mL min^−1^ and the injection volume 500 μL. UV absorbance was monitored at a wavelength of 276 nm. Data were analysed with Chromeleon software.

NMR spectra were recorded on a Bruker AVANCE III 400 MHz spectrometer at 298 K. Chemical shifts are reported in parts per million (ppm) relative to the solvent residual peak of acetone (^1^H: 2.05 ppm, quintet; ^13^C: 29.92 ppm, septet).

For high-resolution electrospray mass spectrometry (HR-ESI-MS) samples were diluted into 50% ACN/0.1% formic acid and infused into a Synapt G2-Si mass spectrometer (Waters, Manchester, UK) at 10 μL min^−1^ using a Harvard Apparatus syringe pump. The mass spectrometer was controlled by Masslynx 4.1 software (Waters). It was operated in positive ion mode and calibrated using sodium iodide. The sample was analysed for 1 min with a 1 s MS scan time over the range of 50–1200 *m*/*z* with 3000 V capillary voltage, 40 V cone voltage, 115 °C cone temperature. Leu-enkephalin peptide (1 ng μL^−1^, Waters) was infused at 10 μL min^−1^ as a lock mass (*m*/*z* = 556.2766) and measured every 10 s. Spectra were generated in Masslynx 4.1 and peaks were centred using automatic peak detection with lock mass correction.

The specific optical rotation of compounds was measured with a Model 341 Polarimeter (PerkinElmer, Inc.).

#### Purification of 1–4

OPM was inoculated with a single colony of *P. fluorescens* ATCC 39502 (WT for 1 and Δ*obaL* for analogue production) and incubated at 25 °C with shaking at 300 rpm. After 24 h, 8 L of OPM (16 × 500 mL in 2 L flasks) were each inoculated with 5 mL from the starter culture; for analogue production 2 mL of a 100 mM stock solution of either 2-HBA, 3-HBA or BA in DMSO was added to give a final concentration of 0.4 mM. After incubation for a further 14 h at 25 °C with orbital shaking at 250 rpm, ethyl acetate (500 mL) was added to each flask and the cultures were shaken vigorously and then left to stand for ∼2 h. The organic phase was then separated, and the solvent removed under reduced pressure.

Extracts containing 1, 2 or 3 were dissolved in ACN/water (1 : 1; total volume 4 mL) and fractionated over a Biotage C_18_ 30 g cartridge using a Biotage Isolera™ system with UV absorbance monitoring at 276 nm. Mobile phase A: water; mobile phase B: ACN; flow rate: 25 mL min^−1^. Elution started from 3% B for 1.5 column volumes (CV), then gradient to 30% B over 1 CV, then gradient to 80% B over 7.5 CV, then gradient to 100% over 1 CV, and holding at 100% B for 1.5 CV. The fractions containing the desired compound were combined and the solvent was removed under reduced pressure. Biotage fractionation of the extract containing either 1 or 2 gave sufficiently pure compound that no further purification was required. Biotage fractionation of the extract containing 3 gave material which required further purification by the method described for preparative HPLC. The fraction containing 3 was collected and dried under reduced pressure.

The extract containing 4 was dissolved in ACN/water (1 : 1, total volume 6 mL). Drawing this solution up through a long needle (Sterican 0.80 × 120 mm 21G) caused immediate precipitation in the syringe. The precipitant was carefully filtered, dried under a stream of nitrogen, and washed with ice cold acetone (3 mL) to give 4. Additional 4 was recovered by addition of water (1 mL) to the acetone, causing it to precipitate.

The process was repeated nine times at 8 L scale to give 72 L of producing culture for 1, and three times at 8 L scale giving 24 L of producing culture for 2, 3, and 4.

1: 1468 mg (20.4 mg mL^−1^) as a light purple powder; [*α*]_D_ +69° (*c* = 0.32, ACN) (Literature [*α*]_D_ + 70°, (*c* = 0.1, ACN)); HRMS (ESI) *m*/*z*: calculated for C_17_H_15_N_2_O_7_ ([M + H]^+^) = 359.0874, observed = 359.0872 ([M + H]^+^), *Δ* = −0.6 ppm. For tabulated NMR see Table S2 (ESI†).

2: 327 mg, (13.6 mg mL^−1^) as an off-white powder; [*α*]_D_ +115° (*c =* 1.0, EtOAc); HRMS (ESI) *m*/*z*: calculated for C_17_H_15_N_2_O_6_ ([M + H]^+^) 343.0925, observed 343.0930 [M + H]^+^, *Δ* = 1.5 ppm. For tabulated NMR see Table S3 (ESI†).

3: 182 mg, (7.6 mg L^−1^), as an off-white powder; [*α*]_D_ +66° (*c =* 0.5, EtOAc); HRMS (ESI) *m*/*z*: calculated for C_17_H_15_N_2_O_6_^+^ ([M + H]^+^) = 343.0925, observed [M + H]^+^ = 343.0933, *Δ* = 2.3 ppm. For tabulated NMR see Table S4 (ESI†).

4: 369.9 mg, (16.5 mg mL^−1^) as a bright white powder; [*α*]_D_ +45° (*c =* 0.16, EtOAc); HRMS (ESI) *m*/*z*: calculated for C_17_H_15_N_2_O_5_ ([M + H]^+^) = 327.0975, observed [M + H]^+^ = 327.0963, *Δ* = −3.7 ppm. For tabulated NMR see Table S5 (ESI†).

#### Preparation of compounds 5–7

##### Hydrolysis of 1 to yield 5

1 (6.0 mg, 16.7 μmol) was dissolved in aqueous NaOH (0.1 M; 500 μL) and stirred for 3 h at room temperature. Hydrochloric acid (0.1 M, 500 μL) was then added, and the reaction mixture dried under reduced pressure. The resulting dry mixture was dissolved in water/acetonitrile (3 : 1, 1 mL) and fractionated using the preparative HPLC method. The major fraction was dried under reduced pressure to give 5 (3.2 mg, 51%) as a brown oil; [*α*]_D_ +35° (*c* = 0.2, acetone); HRMS (ESI) *m*/*z*: calculated for C_17_H_17_N_2_O_8_^+^ ([M + H]^+^) = 377.0979, observed [M + H]^+^ = 377.0976, *Δ* = −0.8 ppm. For tabulated NMR see Table S6 (ESI†).

##### Methanolysis of 1 to yield 6

Triethylamine (20 μL, 0.14 mmol) was added to a solution of 1 (6.0 mg, 16.7 μmol) in dry methanol (2 mL) under argon. The reaction mixture was stirred for 3 h at room temperature, after which time it was dried under vacuum. The residue was dissolved in water/acetonitrile (3 : 1, 1 mL) and fractionated using the preparative HPLC method. The single major fraction was dried under reduced pressure to give 6 (6.1 mg, 93%) as a brown oil; [*α*]_D_ +51° (*c* = 0.4, acetone); HRMS (ESI) *m*/*z*: calculated for C_18_H_19_N_2_O_8_^+^ ([M + H]^+^) = 391.1136, observed [M + H]^+^ = 391.1131, *Δ* = −1.3 ppm. For tabulated NMR see Table S7 (ESI†).

##### Hydrolysis of 4 to yield 7

LiOH·H_2_O (9.6 mg, 230 μmol) was added to a solution of 4 (15 mg, 46 μmol) in dioxane/water (15 mL, 1 : 1). The reaction mixture was stirred for 3 h at room temperature, after which time hydrochloric acid (1 M) was added dropwise until the reaction mixture went from dark red to light yellow. The volatile components were removed under reduced pressure and the resulting dry mixture was dissolved in water/acetonitrile (3 : 1, 1 mL) and fractionated using the preparative HPLC method. The major fraction was dried under reduced pressure to give 7 (4.6 mg, 29%) as a white powder; [*α*]_D_ +41° (*c* = 0.2, acetone); HRMS (ESI) *m*/*z*: calculated for C_17_H_17_N_2_O_6_^+^ ([M + H]^+^) = 345.1081, observed [M + H]^+^ = 345.1071, *Δ* = −2.9 ppm. For tabulated NMR see Table S8 (ESI†).

### Metal binding experiments

#### Chrome azurol S (CAS) Fe^3+^ binding assay

##### Preparation of CAS assay solution

To prepare the CAS assay solution, CAS (7.5 mL of 2 mM in water) was mixed with FeCl_3_·6H_2_O (1.5 mL of 1 mM dissolved in 10 mM HCl). The resulting solution was gradually added to an aqueous solution of hexadecyltrimethyl-ammonium bromide (HDTMA; 25 mL, 2.4 mM) with stirring. To this, MES buffer (50 mL, 1 M, pH 5.6) was added and the final volume was adjusted to 100 mL with H_2_O.

**Table:** 

Reagent	Volume (mL)	Final concentration
1 M MES (pH 5.6)	50	500 mM
2.4 mM HDTMA	25	600 μM
2 mM CAS	7.5	150 μM
1 mM FeCl_3_·6H_2_O in 10 mM HCl	1.5	15 μM
ddH_2_O	To 100	

##### CAS Fe^3+^ binding assay

A dilution series (50 mM to 50 μM) of 1–4 were prepared in DMSO. In a microtitre plate, 90 μL of CAS solution was added to 10 μL of compound each dilution, to give final analyte concentrations of 5000 to 5 μM. The negative control was 10% v/v DMSO in CAS solution. The microtitre plates were imaged with a Perfection V600 scanner (Epson) and UV spectra were recorded from 220 to 800 nm on a microplate reader (Clariostar).

#### 4-(2-Pyridylazo)-resorcinol (PAR) Zn^2+^ binding assay

##### Preparation of PAR assay solution

To prepare the Zn(PAR)_2_ solution, aqueous ZnSO_4_ (100 μL, 1 mM) was added to PAR solution in PBS (8.9 mL, 30 μM, pH 7.0).

**Table:** 

Reagent	Volume (mL)	Final concentration (μM)
30 μM PAR in PBS (pH 7.0)	8.9	29.7
1 mM ZnSO_4_	0.1	11.1

##### PAR Zn^2+^ binding assay

A dilution series (2500 to 50 μM) of 1–7, 2,3-DHBA, 2-HBA, 3-HBA, *N*-acetyl-threonine (*N*-Ac-Thr) and *N*,*N*,*N*′,*N*′-tetrakis-(2-pyridylmethyl)ethylenediamine (TPEN) were prepared in DMSO. In a microtitre plate, 10 μL of each compound dilution was added to 90 μL of Zn(PAR)_2_ solution, to give final analyte concentrations of 250–5 μM. The negative control was 10% v/v DMSO in Zn(PAR)_2_ solution, and the positive controls were 10% v/v DMSO in PAR solution, and the dilution series of TPEN in Zn(PAR)_2_ solution. The microtitre plates were imaged with a Perfection V600 scanner (Epson) and UV spectra were recorded from 220–800 nm on a microplate reader (Clariostar).

#### Analysis of metal binding by mass spectrometry

To assess the metal binding 1 was mixed with aqueous solutions of FeSO_4_·7H_2_O, FeCl_3_·6H_2_O, ZnSO_4_·7H_2_O, MgCl_2_·6H_2_O, MnCl_2_·4H_2_O or Ga(NO_3_)_3⋅_9_H_2_O_, or an equimolar mixture of all salts to give final concentrations of 100 μg mL^−1^1 (279 μM) and 1 mM for each metal ion. Compounds 2–4 (100 μg mL^−1^) were mixed with FeCl_3_·6H_2_O (1 mM). Samples were analysed on a Synapt G2-Si mass spectrometer as described above.

#### Job plots to determine the 1-Fe^3+^ complex stoichiometry

To determine the stoichiometry of the 1-Fe^3+^ complex, the change in absorbance was measured by UV-visible spectroscopy across a series of molar ratios. Samples with a mole fraction range of 1:Fe^3+^ from 0.1 to 0.9 were prepared as follows: aliquots of 1 (from 10 to 90 μL of a 1 mM stock solution in DMSO) and Fe(NO_3_)_3_·9H_2_O (from 90 to 10 μL of a 1 mM stock solution in water) were mixed in a 96-well microtitre plate. The solutions were left at room temperature for 15 min to equilibrate, then the UV-visible spectrum was measured using a microplate reader (Clariostar). The *λ*_max_ of the 1-Fe^3+^ complex was determined as 690 nm, and the absorbance for each sample was plotted against the mole fraction to give a Job plot. Salicylic acid (2-HBA; 1 mM in DMSO) was used as a positive control, as it forms a 1 : 1 complex with Fe^3+^ with a *λ*_max_ at 535 nm. [Reid 2008]^51^ This analysis produced a maximum at 0.5 molar ratio indicating a 1 : 1 1 : Fe^3+^ complex.

### Protein expression and purification

#### Construction of expression plasmids

Plasmids for protein expression were generated as previously described^12^ using Gibson assembly (Tables 6 and 7).

**Table 6:** Oligonucleotides used in this work

Oligonucleotide	Sequence 5′-3′	Description
pET28a(+)-PfThrRS-Fwd	TGGTGCCGCGCGGCAGCC̲A̲T̲**A̲T̲G̲CCAACTATTACTCTACCC**	Designed to clone entire PCS of PfThrRS as a *NdeI-XhoI* fragment into pET28a(+) using Gibson assembly
pET28a(+)-PfThrRS-Rev	CAGTGGTGGTGGTGGTGGTGC̲T̲C̲G̲A̲G̲**TTACTCCGAATCTGGGCG**	

**Table 7:** Plasmids used in this work

Plasmid	Description	Ref.
pET28a(+)	Expression vector; Kan^R^, the transcription of the cloned gene is driven by the T7 RNA polymerase and controlled by the LacI repressor, *ColE1* replicon	Novagen
pET28a(+)-*EcThrRS*	For the expression of the His_6_-EcThrRS protein	Scott *et al.* 2019^12^
pET28a(+)-*obaO*	For the expression of the His_6_-ObaO protein	Scott *et al.* 2019^12^
pET28a(+)-*PfThrRS*	For the expression of the His_6_-PfThrRS protein	This work

#### Protein purification

*E. coli* NiCo21(DE3) (NEB) carrying pET28a(+)-*EcThrRS*, pET28a(+)-*PfThrRS* or pET28a(+)-*obaO* was used for protein expression and purification. Immobilised metal affinity chromatography (IMAC) and size exclusion chromatography (SEC) was used as previously described.^24^ Proteins were purified in a buffer containing 20 mM Tris–HCl at pH 8.0 with 500 mM NaCl and 10 mM MgCl_2_. The IMAC elution buffer also contained 250 mM imidazole.

### *In vivo* tRNA^Thr^ transcription and purification

To obtain purified tRNA^Thr^ for aminoacylation assays, tRNA^Thr^ was overexpressed in *E. coli* and purified by phenol extraction, as described in Avcilar-Kucukgoze *et al.*^52^ with some modifications as follows. A LB starter culture (10 mL) was inoculated from a single colony of *E. coli* MRE600:pWFW1015 and incubated for 16 h at 37 °C with 250 rpm shaking. This was used to inoculate LB (1 L containing 100 μg mL^−1^ carbenicillin in a 2 L Erlenmeyer flask) and incubated at 30 °C until the OD_600_ reached 0.4–0.6, and then induced with IPTG (1 mM final concentration) and grown for a further 16 h at 30 °C. The cells were collected by centrifugation at 5000 × *g* for 25 min at 4 °C, and total RNA extracted by resuspension of the pellet in solution 1 (8 mL) followed by the addition of acid phenol (Sigma-Aldrich; 17.2 mL, pH 4.5); (solution 1 = sodium acetate, 50 mM; magnesium acetate, 10 mM; pH 5.0). The resulting emulsion was shaken on an orbital incubator at 215 rpm for 30 min at 37 °C, then centrifuged at 5000 × *g* for 15 min at 4 °C and the resulting aqueous layer was collected and transferred to a clean tube. The phenol/interface layer was extracted a second time by addition of Solution 1 (14 mL) followed by orbital shaking at 215 rpm for 15 min at 37 °C. This was followed by centrifugation at 5000 × *g* for 15 min at 4 °C and the aqueous phases were combined. Subsequent precipitation of total nucleic acids was achieved by addition of NaCl solution (5 M) to give a final concentration of 0.2 M, followed by 1 volume of isopropanol. The mixture was allowed to sit at room temperature briefly before centrifugation at 14 500 × *g* for 15 min at room temperature. The resulting nucleic acid pellet was washed with ice cold 70% ethanol, then air dried for 5–10 min.

The contaminating rRNA was then removed by dissolution of tRNAs by vortex mixing in ice cold NaCl solution (10 mL, 1 M), followed by centrifugation at 9500 × *g* for 20 min at 4 °C. The supernatant was collected, and the remaining pellet was again vortexed in cold NaCl solution (5 mL, 1 M) and centrifuged again. The supernatants were combined, and the soluble nucleic acids were precipitated by addition of 2 volumes of cold ethanol and standing for 30 min at −20 °C, followed by centrifugation at 14 500 × *g* for 5 min at 4 °C. The resulting pellet was washed with 70% ethanol and air dried for 5–10 min. Contaminating DNA was removed by heating the pellet in NaOAc solution (6 mL, 0.3 M, pH 5.0) at 60 °C until it dissolved, followed by addition of isopropanol (3.4 mL) and standing for 10 min at room temperature. This was then centrifuged at 14 500 × *g* for 5 min at room temperature and the supernatant collected. The precipitation of any remaining tRNAs was achieved by addition of isopropanol (2.3 mL) and standing for 30 min at −20 °C. The suspension was then centrifuged at 14 500 × *g* for 15 min at 4 °C, the pellet washed with 70% ethanol and air dried for 5–10 min and the resulting dry pellet dissolved in DEPC-treated water (500 μL). The tRNA was deacylated by addition of Tris buffer (35 μL, 1.5 M, pH 9.0) and incubated for 45 min at 37 °C. This was followed by addition of NaOAc solution (53.5 μL, 3 M, pH 5.0) and ethanol (1.6 mL), followed by standing for 30 min at −80 °C. The resulting suspension was then centrifuged at 16 100 × *g* for 25 min at 4 °C, and the pellet washed with 70% ethanol and air dried. The pellet was finally dissolved in DEPC-treated water (250–300 μL). The concentration and quality of tRNA was assessed by Nanodrop using a *A*_260_/*A*_280_ ratio (observed value >2).

### Aminoacylation assays

To measure the effects of 1–4 on ThrRS canonical tRNA charging activity, an assay modified from that reported by Ruan *et al.*^53^ was used, where active enzyme was incubated with its necessary substrates and its activity measured using ^14^C labeled Thr and a liquid scintillation counter. Purified ThrRS protein (10 nM) was pre-incubated with varying concentrations of compounds 1–4 or ACN for 10 min. After pre-incubation, ThrRS samples were added to a master reaction mixture with the final component concentrations of HEPES (100 mM, pH 7.0), ATP (4 mM), MgCl_2_ (10 mM), l-[U-^14^C]threonine (50 μM, Moravek Inc.), and tRNA^Thr^ (5 μM). This mixture was incubated at room temperature for 10 min, with samples taken at 1, 2.5, 5, and 10 min. At each time point, a 7 μL aliquot was spotted onto a 5% trichloroacetic acid (TCA) presoaked 3 MM Whatman paper (Cytiva) squares (repeated in triplicate). After allowing the spots to dry, the Whatman paper was washed three times with 5% TCA, and once with 95% EtOH. The Whatman paper was dried and the counts on each square of paper analyzed using a Tri-Carb 2910 TR liquid scintillation counter using Hydrofluor Liquid Scintillation Fluid (National Diagnostics). To calculate IC_50_ values, fractional initial velocities (velocity of inhibited reaction/velocity in the absence of inhibitor) were plotted against the log of 1 concentration and then fitted to eqn (1), using GraphPad Prism.1
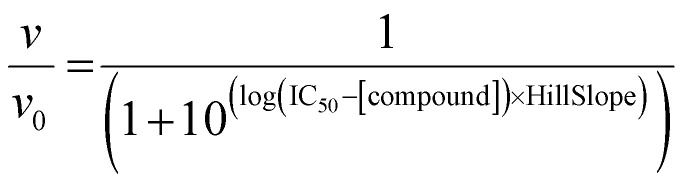


#### Antimicrobial assays

##### Determination of minimum inhibitory concentrations (MIC) using spot-on-lawn bioassays

Indicator strains were grown for 16–18 h in LB (5 mL) by inoculation from a single colony. An aliquot of the resulting culture (500 μL) was then used to inoculate LB (50 mL in 250 mL Erlenmeyer flasks), followed by orbital shaking at 250 rpm until they reached an OD_600_ of 0.3–0.4. These cultures were then diluted 1 : 10 with molten soft nutrient agar (SNA), before pouring into appropriately sized Petri dishes to set. Serial dilutions of the test compounds (from 256 μg mL^−1^ to 1 μg mL^−1^ plus an additional sample at 1000 μg mL^−1^) were prepared in acetonitrile (ACN), and 4 μL of each sample was applied directly onto the SNA surface. Kanamycin (50 μg mL^−1^), apramycin (50 μg mL^−1^) or aureobasidin A (0.2 μg mL^−1^) were used as positive controls, as appropriate, and ACN or water (4 μL) were used as negative controls. Plates were incubated for 16–18 h and the MIC was defined as the lowest concentration of compound that resulted in a zone of inhibition. Experiments were carried out in at least triplicate for each strain.

##### MIC determination under iron depleted/replete conditions

The method was the same as for the antibacterial MIC assays described above, with the following modifications: for excess iron, Fe(NO_3_)_3_·9H_2_O stock (400 mM) was added to give a final concentration of 1.5 or 2.0 mM; for iron depletion, bipy stock (1 M) was added to give a final concentration of 150 μM. For MRSA, 1 was further diluted to 0.5 0.25 and 0.125 μg mL^−1^ due to a decrease in MIC under excess iron conditions. The general effect of excess iron on the activity of alternative antibiotics was determined by performing a similar serial dilution of carbenicillin, kanamycin, streptomycin, nitrofurantoin and chloramphenicol in the presence of Fe^3+^.

### Monitoring the hydrolysis protection effect of Fe^3+^ binding for obafluorin (1) and analogues 2–4 by HPLC

Five solutions of HEPES buffer (111 mM) were prepared at pH 6.0, 6.5, 7.0, 7.5 and 8.0 respectively. A further five solution of HEPES buffer (111 mM) with Fe(NO_3_)_3_.9H_2_O (11.1 mM) were also prepared at pH 6.0, 6.5, 7.0, 7.5, 8.0 respectively. Stock solutions of 1–4 (10 mM) were prepared in DMSO. For each hydrolysis experiment, stock solution of 1–4 (10 μL) was added the HEPES buffer (90 μL) or the HEPES + Fe(NO_3_)_3_.9H_2_O buffer (90 μL), to give final concentrations of HEPES (100 mM), 1–4 (1 mM) and Fe^3+^ (0 or 1 mM). The reaction mixtures were shaken using a bench top vortex and left to stand for 30 min, after which the samples were analysed by HPLC using the Analytical HPLC method. All stock solutions of 1–4 were tested at every pH, with and without Fe^3+^ and all experiments were repeated in triplicate.

## Author contributions

S. F. D.: conceptualization; methodology; validation; investigation; supervision; writing – original draft; writing – reviewing & editing; visualization. M. J. D.: conceptualisation; methodology; investigation. E. S. H.: validation; investigation; supervision; writing – review & editing; visualization. J. D. L.: methodology; investigation; formal analysis; visualisation. T. A. S.: conceptualization; investigation; S. A.: investigation. C. S. F.: methodology. B. W.: conceptualization; methodology; resources; writing – reviewing & editing; supervision; project administration; funding acquisition.

## Conflicts of interest

The authors declare no competing interests.

## Supplementary Material

CB-004-D3CB00127J-s001
