# Supplementary material for: The catechol moiety of obafluorin is essential for antibacterial activity
Source: RSC Chem Biol. 2023 Aug 21;4(11):926–41. doi: 10.1039/d3cb00127j (PMC10619133; doi:10.1039/d3cb00127j)
Supplement: CB-004-D3CB00127J-s001 [file CB-004-D3CB00127J-s001.pdf]

Electronic Supplementary Material (ESI) for RSC Chemical Biology.
This journal is © The Royal Society of Chemistry 2023

Supporting Information

The catechol moiety of obafluorin is essential for antibacterial activity

Sibyl F. D. Batey[‡a], Melissa J. Davie[‡a], Edward S. Hems[‡a], Jonathon D. Liston[a], Thomas A. Scott[a], Silke Alt[a], Christopher S. Francklyn[b] & Barrie Wilkinson*[a]

[a]*Department of Molecular Microbiology, John Innes Centre, Norwich Research Park, Norwich NR4 7UH, UK. E-mail: barrie.wilkinson@jic.ac.uk*

[b]*Department of Biochemistry, College of Medicine, University of Vermont, Burlington, Vermont 05405, United States. E-mail: christopher.francklyn@med.uvm.edu*

[‡]These authors contributed equally to this work

Table of Contents

Chrome azurol S (CAS) assays 2
HRMS Metal Binding 4
Job plots to determine iron complex stoichiometry 6
4-(2-pyridylazo)-resorcinol (PAR) assays 7
Minimum inhibitory concentrations (MIC) bioassays 10
PfThrRS aminoacylation assays 16
Bioassays with modified iron concentrations 17
Hydrolysis of analogues 2-4 in the absence and presence of Fe^{3+} 21
Tabulated NMR and spectra 23
Obafluorin (1) 23
2-HBA-obafluorin (2) 29
3-HBA-obafluorin (3) 35
BA-obafluorin (4) 41
Hydrolysed obafluorin (5) 47
Methanolysed obafluorin (6) 53
Hydrolysed BA-obafluorin (7) 59
References 65

Chrome azurol S (CAS) assays

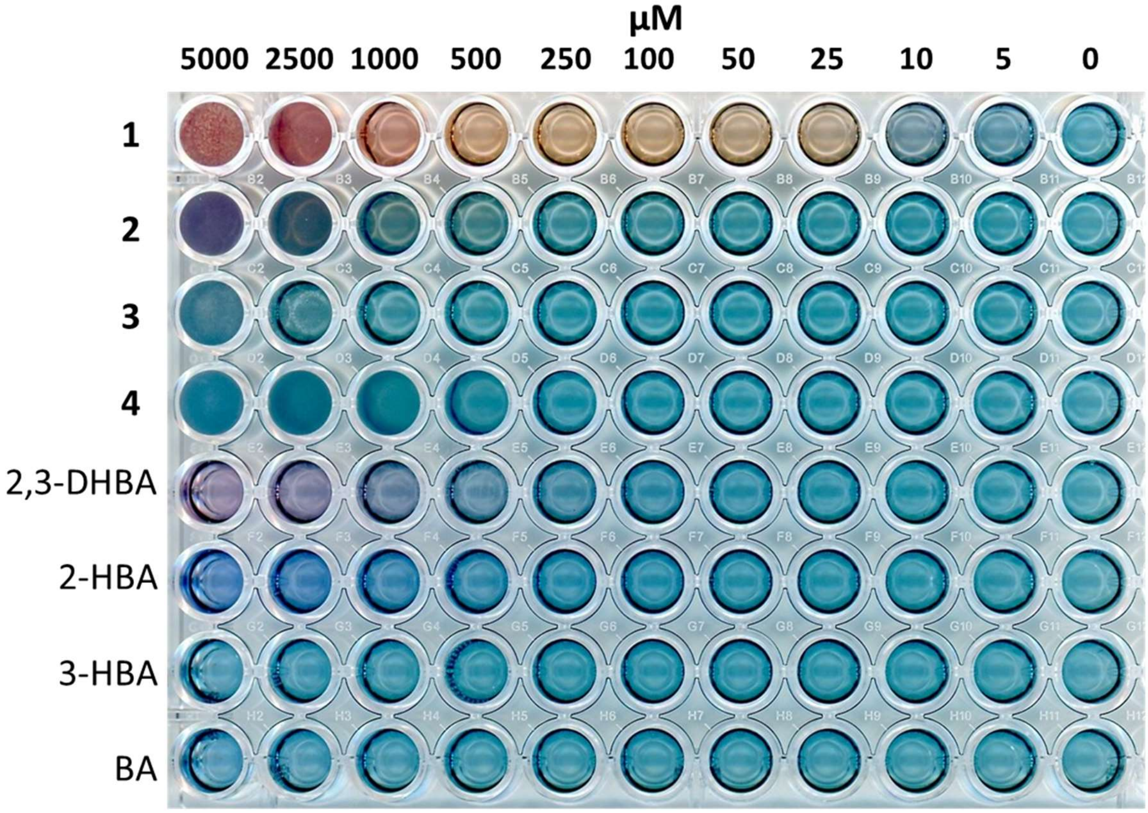

Figure S1: The catechol is essential for the binding of obafluorin (1) to Fe^{3+}. Chrome azurol S (CAS) assay monitoring the binding of Fe^{3+} by **1** and the analogues 2-HBA-obafluorin (**2**), 3-HBA-obafluorin (**3**) and BA-obafluorin (**4**), alongside 2,3-dihydroxybenzoic acid (2,3-DHBA), 2-hydroxybenzoic acid (2-HBA), 3-hydroxybenzoic acid (3-HBA) and benzoic acid (BA) controls. A colour change of blue to orange is indicative of Fe^{3+} binding. [1] Modification of the catechol group in **2**, **3** and **4** abolishes Fe^{3+} binding and **1** shows significantly stronger Fe^{3+} binding than 2,3-DHBA, consistent with the stabilisation of the *ortho*-phenolate by hydrogen bonding of the **1** amide proton. [2] Concentrations of test materials is shown above each column of wells. The concentration of all other components including Fe^{3+} is constant.

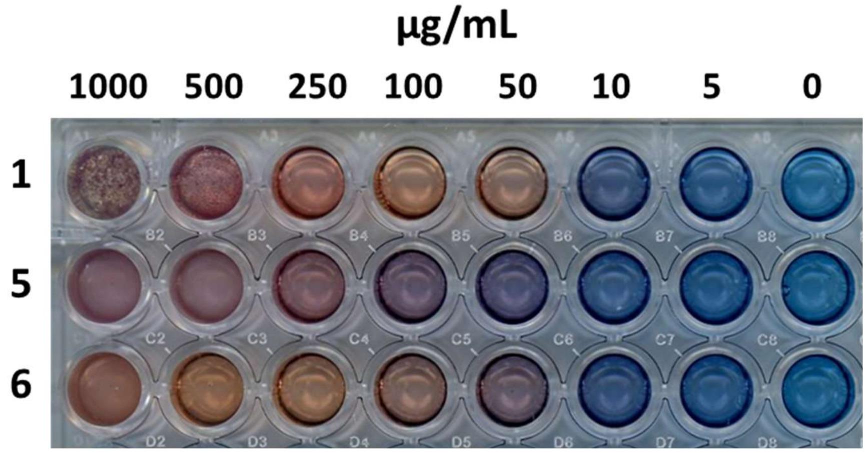

Figure S2: Ring-open 1 analogues bind Fe^{3+}. CAS assay demonstrating ring-open **1** analogues β-lactone hydrolysed obafluorin (**5**) and methanolysed obafluorin (**6**), display similar Fe^{3+} binding to **1**. Concentrations of test substances are shown in µg/mL, with a concentration of 1000 µg/mL equivalent to 2793, 2659 and 2563 µM for **1**, **5** and **6** respectively.

HRMS Metal Binding

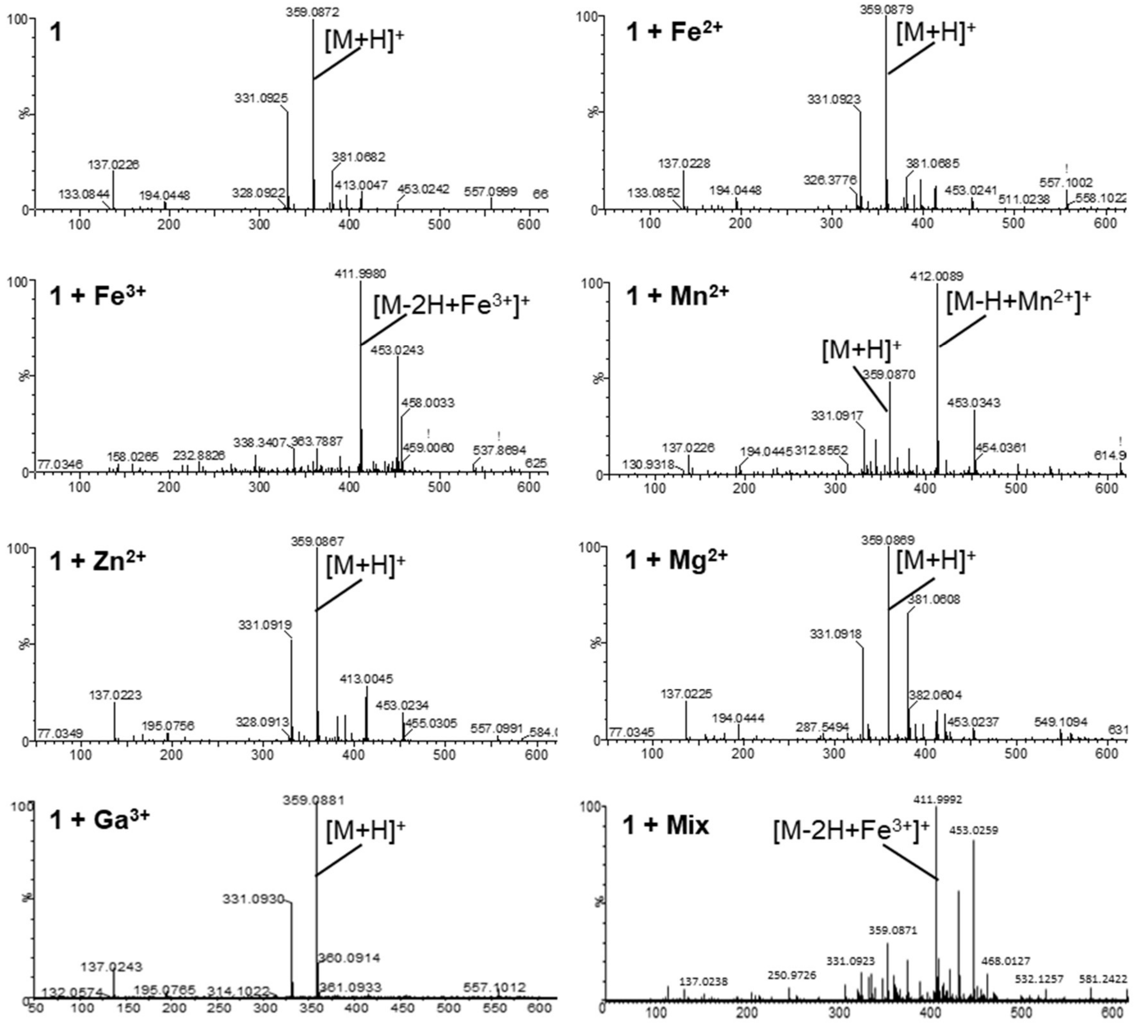

Figure S3: 1 forms a selective interaction with Fe^{3+} detectable by HRMS. 1 was incubated with a range of metal ions and the resultant adducts were monitored by ESI-HRMS on a Synapt G2-Si mass spectrometer. Only in the presence of Fe^{3+} was the $[M+H]^+$ peak fully depleted and the shift to 411.9980 *m/z* demonstrates the formation of a $[M-2H+Fe^{3+}]^+$ species. The additional peak at 453.0243 *m/z* is consistent with the **1**-Fe^{3+} species with an additional acetonitrile (ACN) ligand. In the presence of Mn^{2+}, a peak consistent with $[M-H+Mn^{2+}]^+$ was observed, along with some residual $[M+H]^+$ species. When a mixture of all metal ions tested was added, $[M-2H+Fe^{3+}]^+$ was the major peak. The combined data demonstrate that **1** forms a strong, selective interaction with Fe^{3+}.

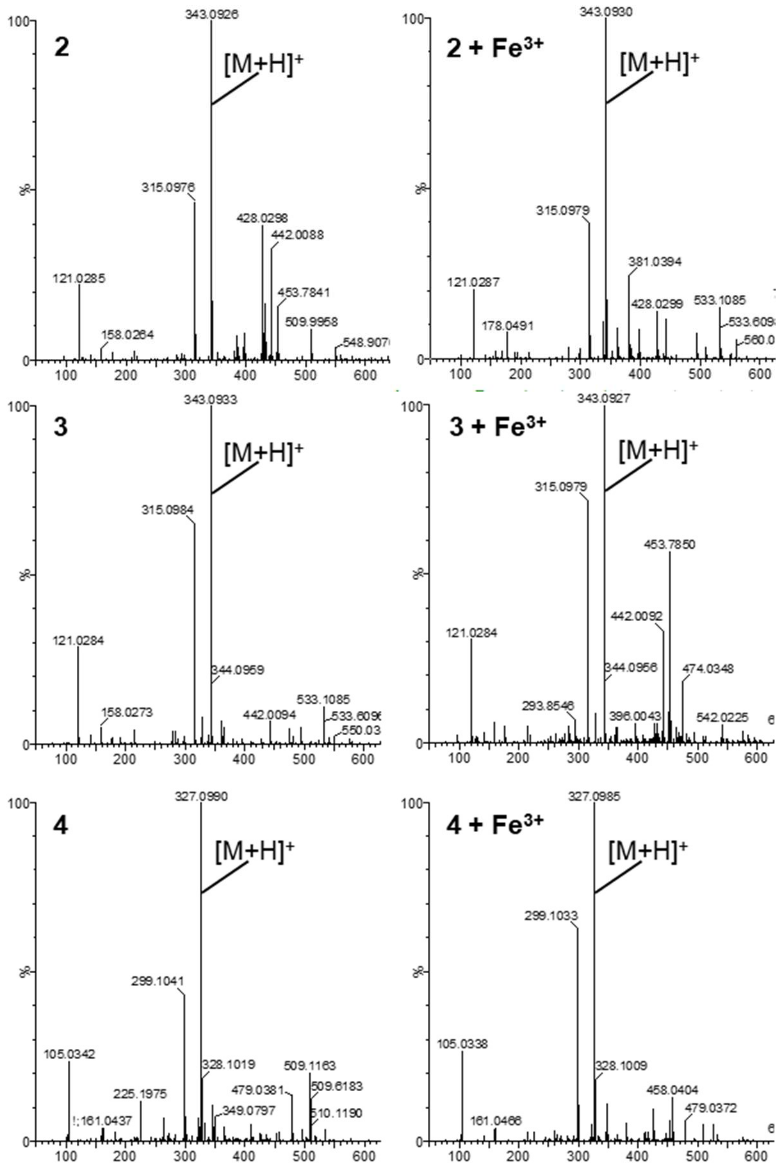

Figure S4: Analogues 2-4 do not bind Fe^{3+} by HRMS. 2-4 were incubated with Fe^{3+} and the resultant adducts were monitored by ESI-HRMS on a Synapt G2-Si mass spectrometer. No binding to Fe^{3+} was observed and the $[M+H]^+$ ions are the major peaks in the presence of Fe^{3+}, in contrast to **1** (Figure S3).

Job plots to determine iron complex stoichiometry

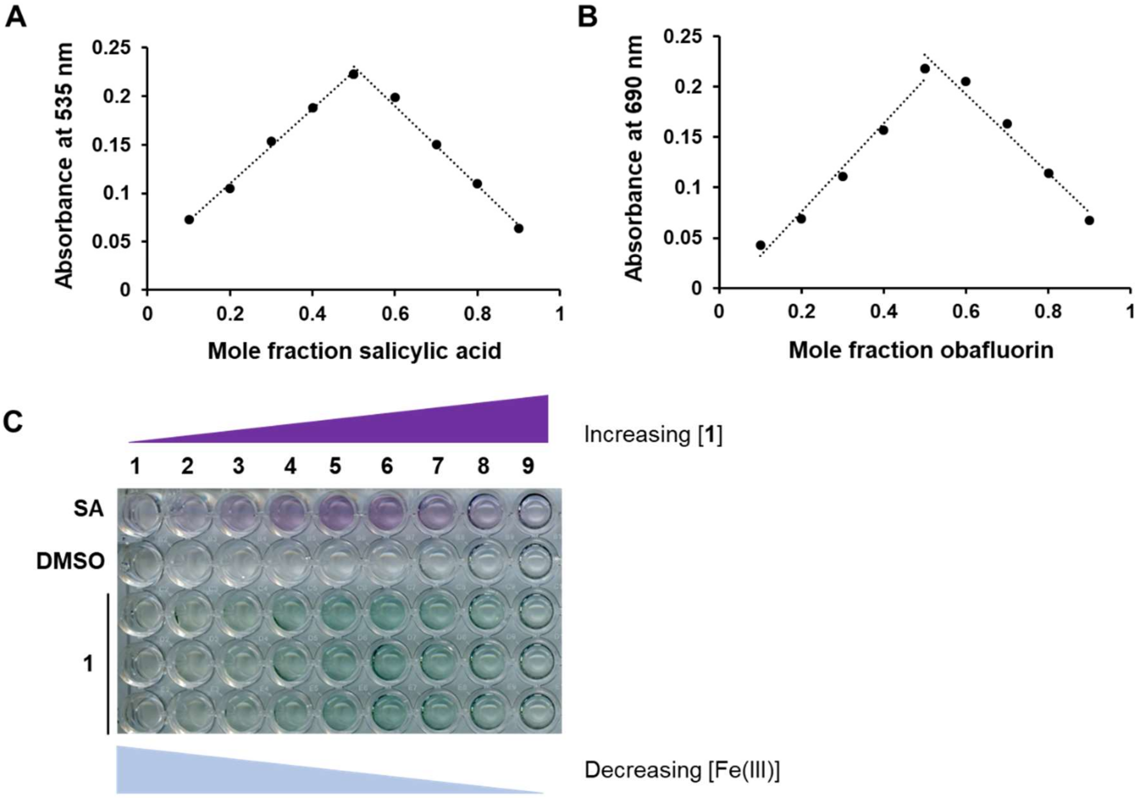

Figure S5: 1 binds Fe^{3+} with a 1:1 stoichiometry. Job plots for measuring iron complex stoichiometry via UV-visible spectroscopy were carried out with (A) salicylic acid (2-HBA) as a positive control and (B) **1.** The λ_{max} of the salicylic acid-Fe^{3+} complex was monitored at 535 nm and the λ_{max} of the **1**-Fe^{3+} complex was determined to be 690 nm in the DMSO:H_2O solvent system employed.[3] Job plots[4] show maximum absorbance at λ_{max} of the respective complexes at mole fraction 0.5, indicating a 1:1 ratio of **1**:Fe^{3+}, in common with the salicylic acid control. The experiments were performed in triplicate and error bars are shown but are too small to be visible. C) Image of 96-well plate with increasing **1/SA**:Fe^{3+} ratio from column 1-9 showing changes in colouration over this range. SA, salicylic acid (positive control), DMSO only (negative control) and **1** in triplicate.

4-(2-pyridylazo)-resorcinol (PAR) assays

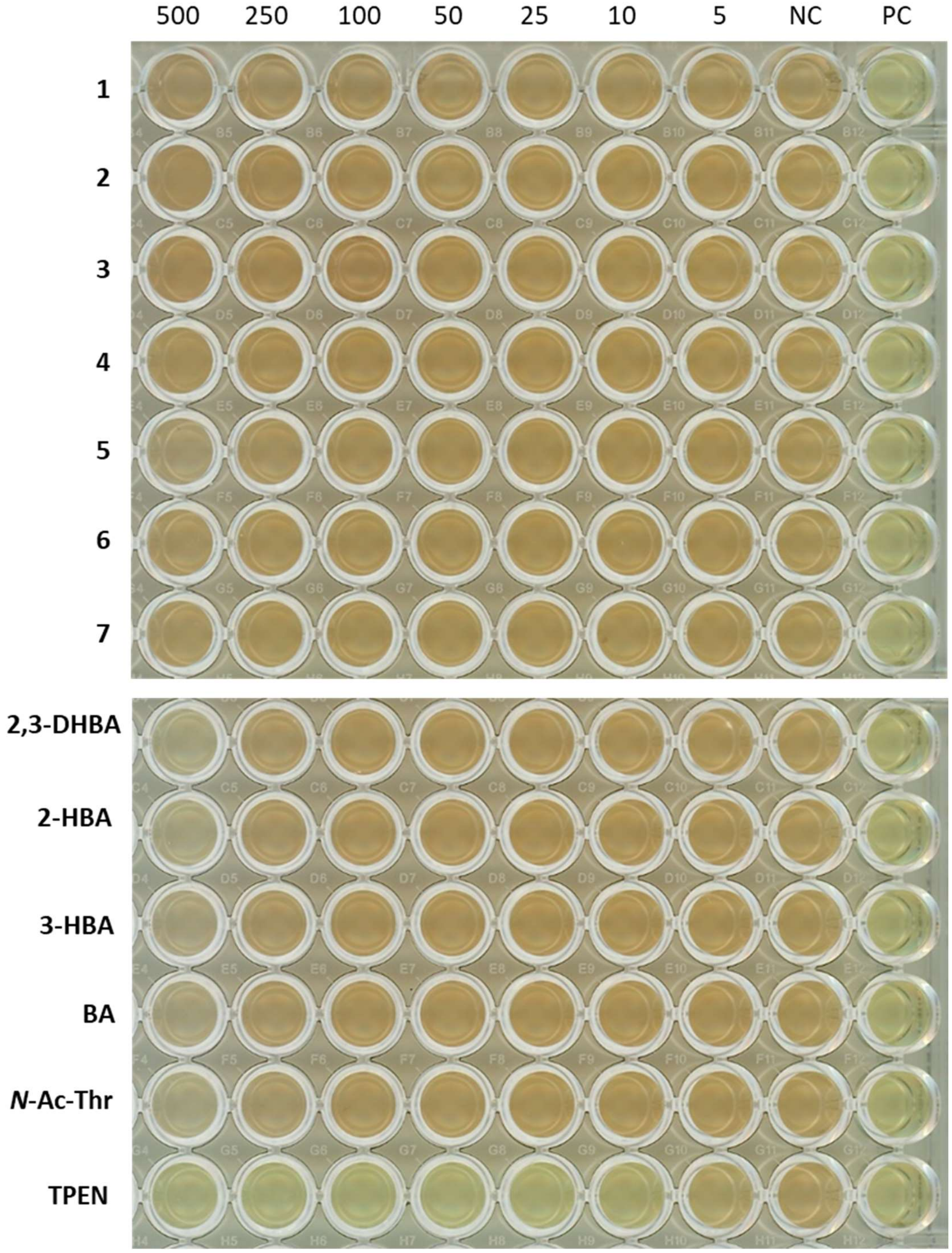

Figure S6: The catechol moiety of 1 shows weak Zn^{2+} binding *in vitro*. Images of PAR assay monitoring the Zn^{2+} binding ability of **1**-**7**, and 2,3-dihydroxybenzoic acid (2,3-DHBA), 2-hydroxybenzoic acid (2-HBA), 3-hydroxybenzoic acid (3-HBA), benzoic acid (BA), *N*-acetyl-threonine (*N*-Ac-Thr) and *N,N,N′,N′*-tetrakis(2-pyridinylmethyl)-1,2-ethanediamine (TPEN). Concentrations are in µM. NC (negative control) = 10% DMSO in $Zn(PAR)_2$ solution. PC (positive control) = 10% DMSO in PAR only solution. The concentration of test substances (µM) are shown above each column of wells. The concentration of all other components including Zn^{2+} were constant.

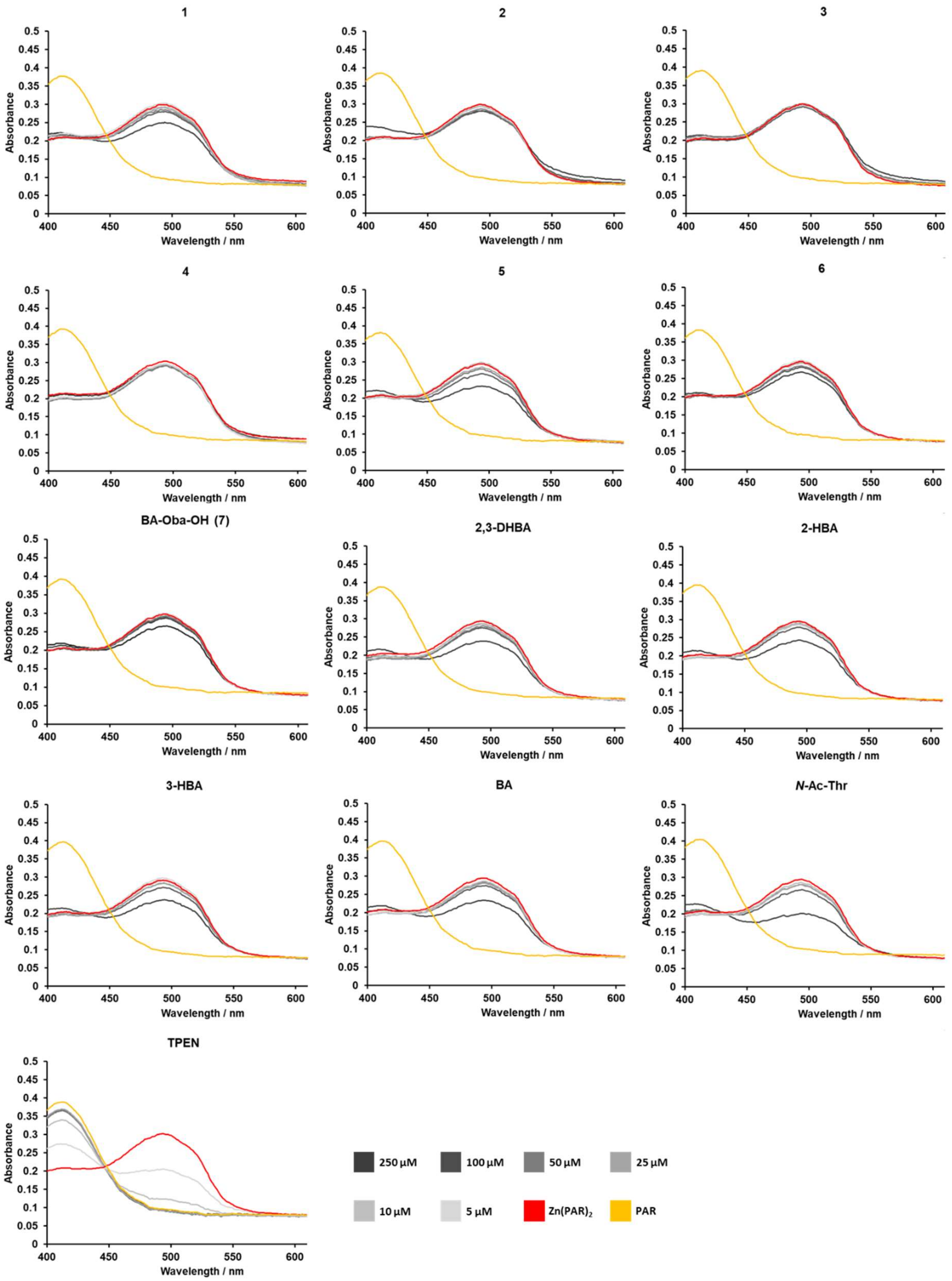

Figure S7: The catechol moiety of 1 shows weak Zn^{2+} binding *in vitro*. Absorbance spectra of **1**-**6** and β-lactone hydrolysed BA-obafluorin (**7**), 2,3-dihydroxybenzoic acid (2,3-DHBA), 2-hydroxybenzoic acid (2-HBA), 3-hydroxybenzoic acid (3-HBA), benzoic acid (BA), *N*-acetyl-threonine (*N*-Ac-Thr) and *N,N,N′,N′*-tetrakis(2-pyridinylmethyl)-1,2-ethanediamine (TPEN) against $Zn(PAR)_2$. PAR (yellow; λ_{max} 410 nm) and Zn^{2+} forms $Zn(PAR)_2$ (red; λ_{max}

495 nm). Titrations of 0.5-25 equivalents of substrate against $Zn(PAR)_2$ (grey gradient) were carried out, with Zn^{2+} binding indicated by a decrease in $Zn(PAR)_2$ and increase in the free PAR as it is displaced from the complex. [5] Whilst the TPEN Zn^{2+} binding positive control showed complete displacement of PAR at molar ratios >1, all **1** and BA analogues showed no or very modest decreases in $Zn(PAR)_2$ and only at the highest molar ratios, indicative of weak or negligible Zn^{2+} binding. We were surprised to observe a decrease in $Zn(PAR)_2$ for the BA control, suggesting that the carboxylic acid group was able to bind Zn^{2+} and thus explaining the similar absorbance spectra for 2,3-DHBA, 2-HBA, 3-HBA and BA. To account for potential interference by free carboxylic acids, compound **7** was included, and comparison of the spectra for **4** and **7** demonstrates a slight contribution to Zn^{2+} binding by the carboxylic acid released in β-lactone hydrolysis. Likewise, N-Ac-Thr was included as an analogue of the hydrolysed β-lactone lacking the aromatic groups to demonstrate the contribution from the β-hydroxy acid. Comparison of compounds with intact β-lactones showed that for **1** but not **2**-**4** there is a modest decrease in $Zn(PAR)_2$ and slight increase in free PAR, suggestive of some weak Zn^{2+} binding via the catechol.

Minimum inhibitory concentrations (MIC) bioassays

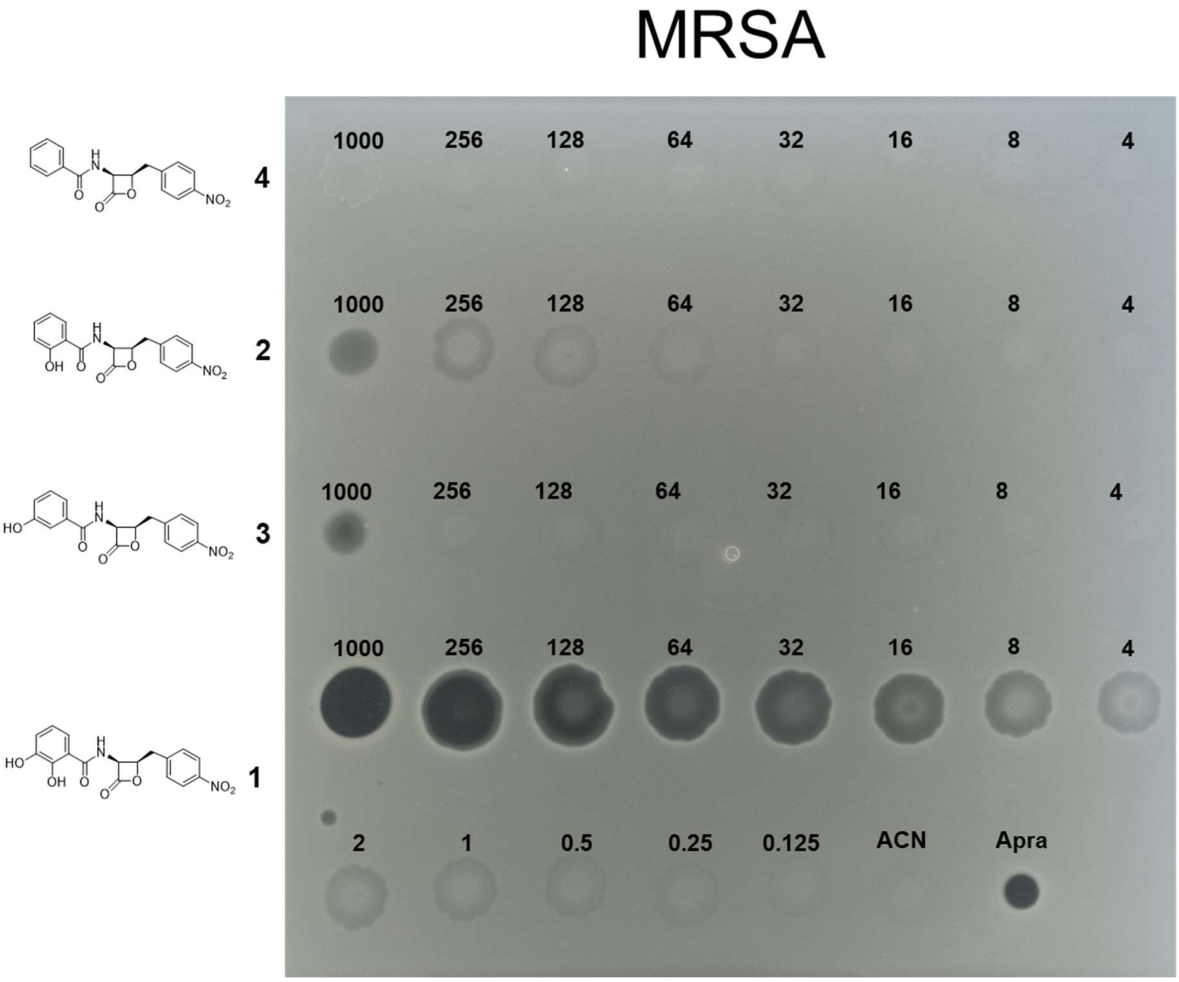

Figure S8: Modification of the 1-catechol abolishes antibacterial activity against methicillin-resistant *S. aureus* (MRSA). Spot-on-lawn bioassay of **1**, **2**, **3** and **4** dissolved in acetonitrile (ACN) against MRSA, with zones of clearing indicating growth inhibition. Numbers indicate concentration of **1, 2, 3** or **4** in µg/mL. Minimum inhibitory concentrations are **1**: 2 µg/mL; **2** and **3**: 1 mg/mL; **4**: > 1 mg/mL. ACN and apramycin (Apra) (50 µg/mL) were used as negative and positive controls respectively. Part of this image was previously published in Scott, Batey *et al*. 2019, Supplementary Figure 1.[6]

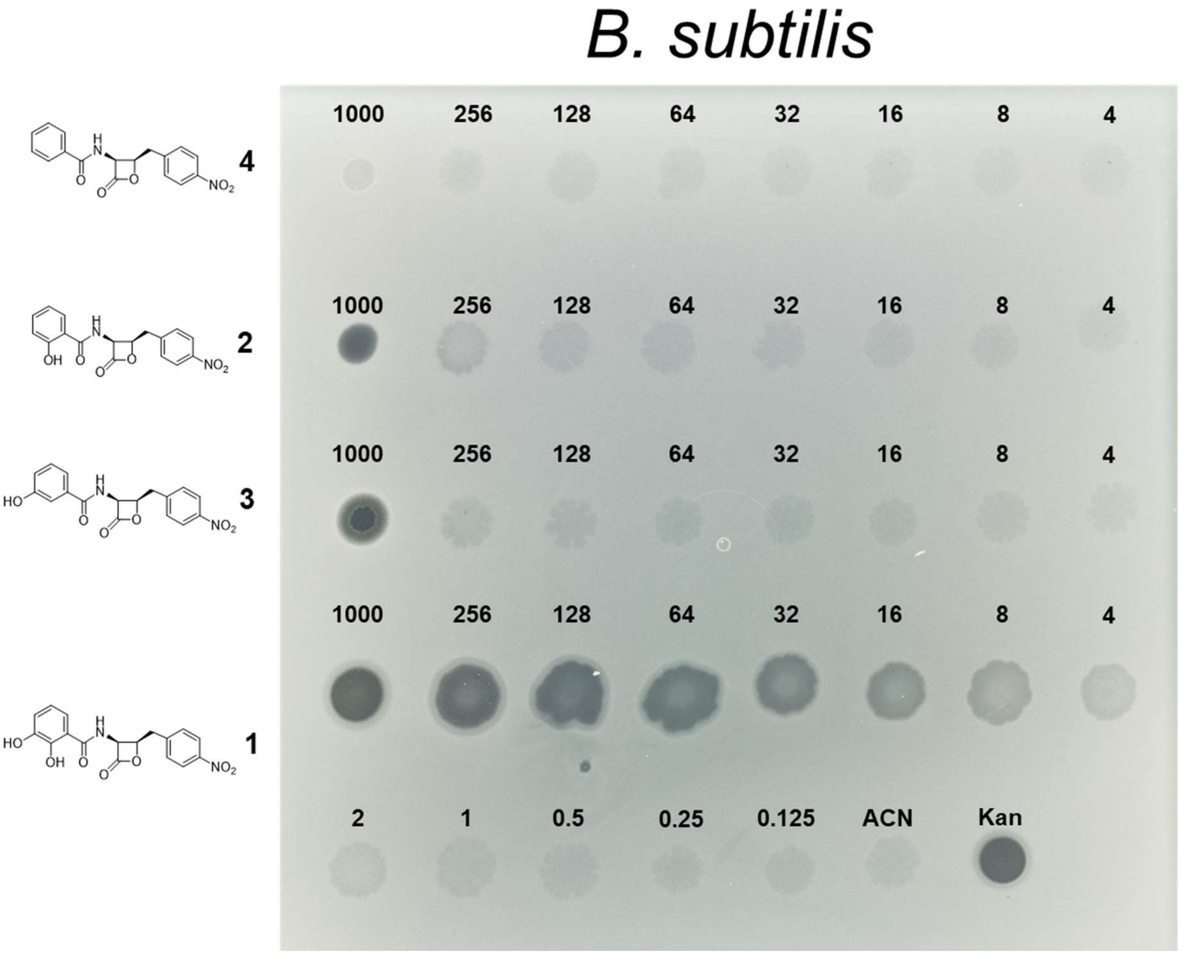

Figure S9: Modification of the 1-catechol abolishes antibacterial activity against *B. subtilis*. Spot-on-lawn bioassay of **1**, **2**, **3** and **4** dissolved in acetonitrile (ACN) against *B. subtilis*, with zones of clearing indicating growth inhibition. Numbers indicate concentration of **1, 2, 3** or **4** in µg/mL. Minimum inhibitory concentrations are **1**: 4 µg/mL; **2** and **3**: 1 mg/mL; **4**: > 1 mg/mL. ACN and kanamycin (Kan) (50 µg/mL) were used as negative and positive controls respectively. Part of this image was previously published in Scott, Batey et al 2019, Supplementary Figure 1.[6]

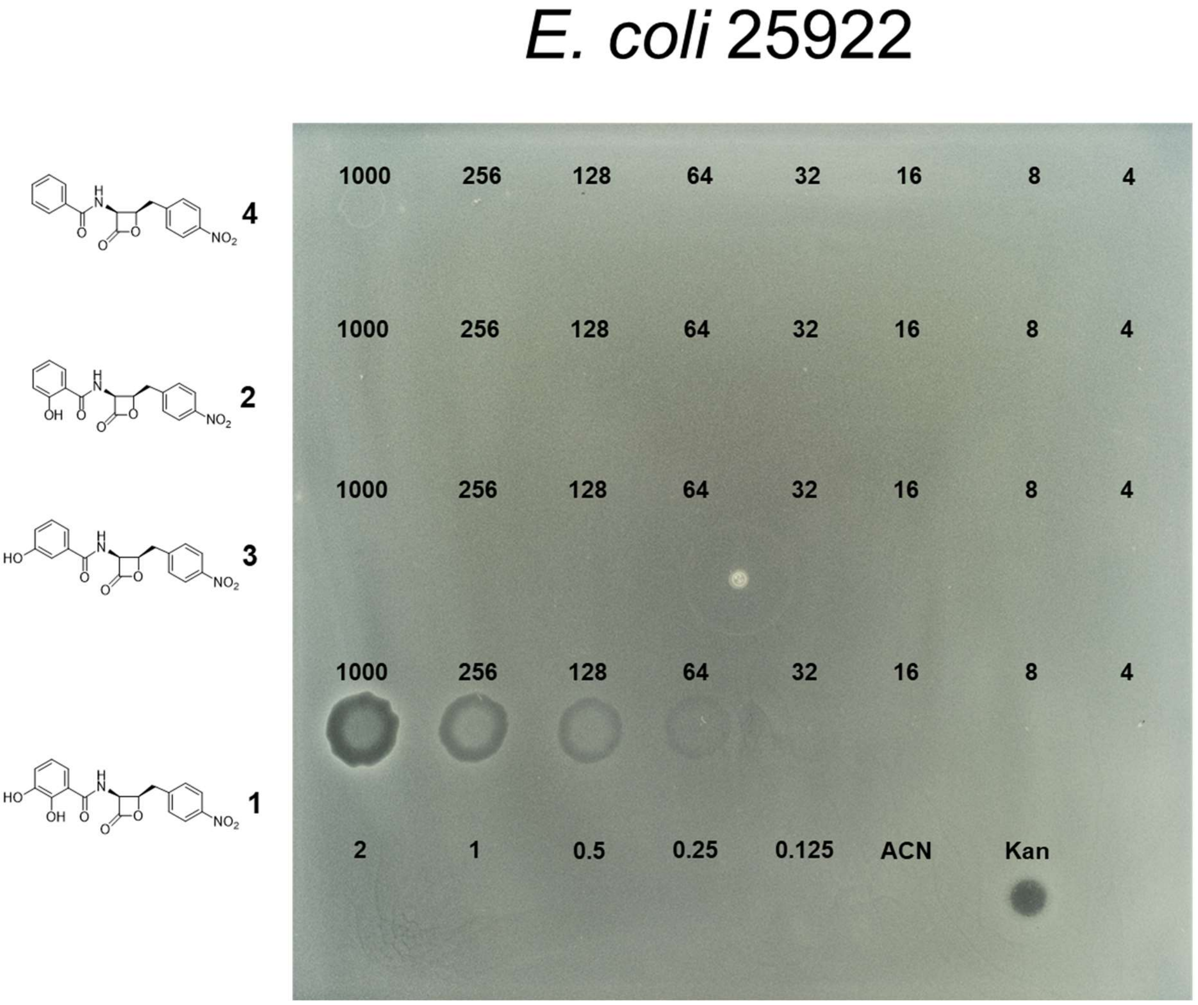

Figure S10: Modification of the 1-catechol abolishes antibacterial activity against *E. coli 25922*. Spot-on-lawn bioassay of **1**, **2**, **3** and **4** dissolved in acetonitrile (ACN) against *E. coli* ATCC25922, with zones of clearing indicating growth inhibition. Numbers indicate concentration of **1, 2, 3** or **4** in µg/mL. Minimum inhibitory concentrations are **1**: 256 µg/mL; **2**, **3** and **4**: > 1 mg/mL. Acetonitrile (ACN) and kanamycin (Kan) (50 µg/mL) were used as negative and positive controls respectively. Part of this image was previously published in Scott, Batey et al 2019, Supplementary Figure 1.[6]

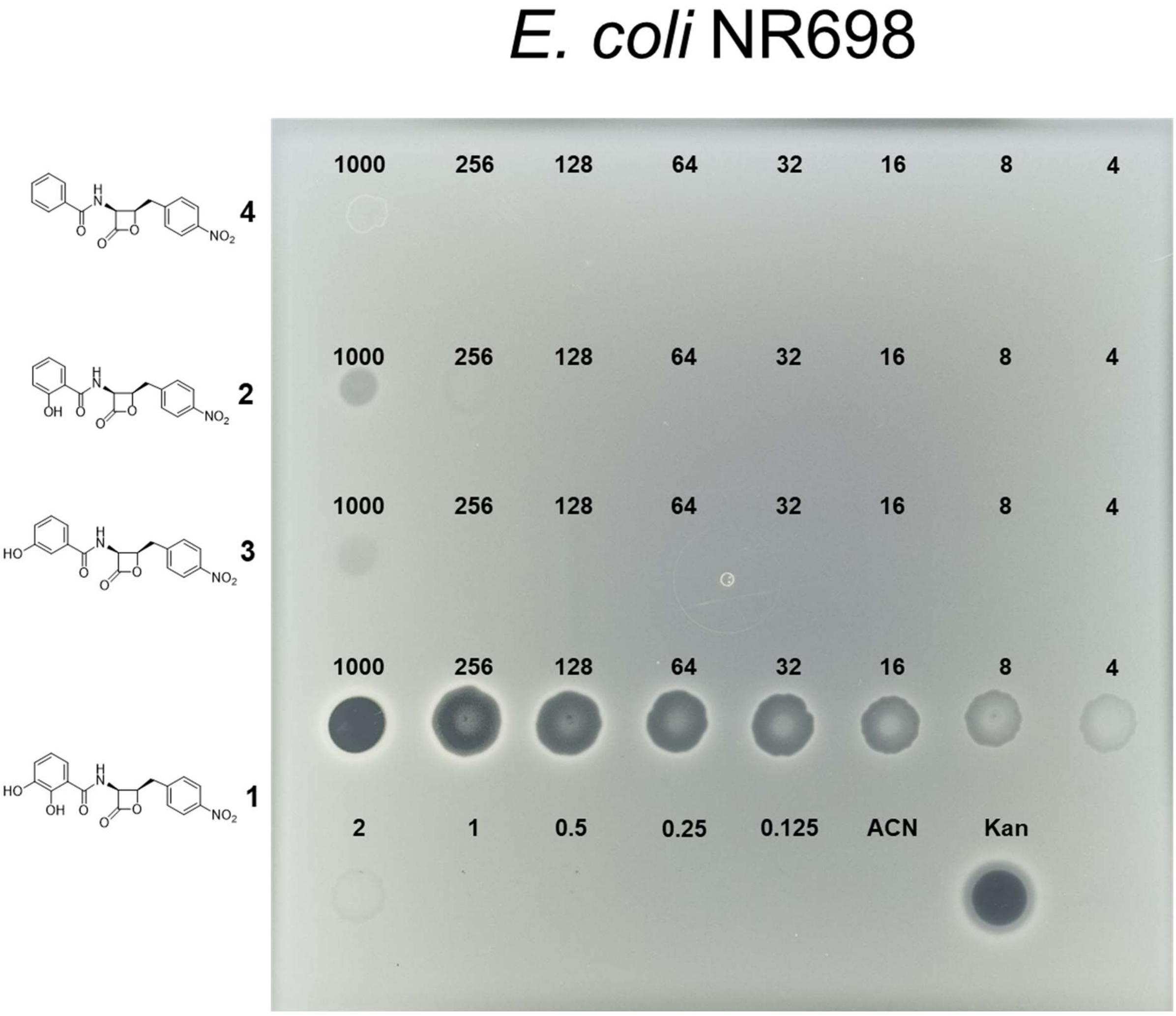

Figure S11: Modification of the 1-catechol abolishes antibacterial activity against *E. coli* NR698. Spot-on-lawn bioassay of **1**, **2**, **3** and **4** dissolved in acetonitrile (ACN) against *E. coli* NR698, with zones of clearing indicating growth inhibition. Numbers indicate concentration of **1, 2, 3** or **4** in µg/mL. Minimum inhibitory concentrations are **1**: 4 µg/mL; **2**, **3** and **4**: > 1 mg/mL. ACN and kanamycin (Kan) (50 µg/mL) were used as negative and positive controls respectively. Part of this image was previously published in Scott, Batey et al 2019, Supplementary Figure 1.[6]

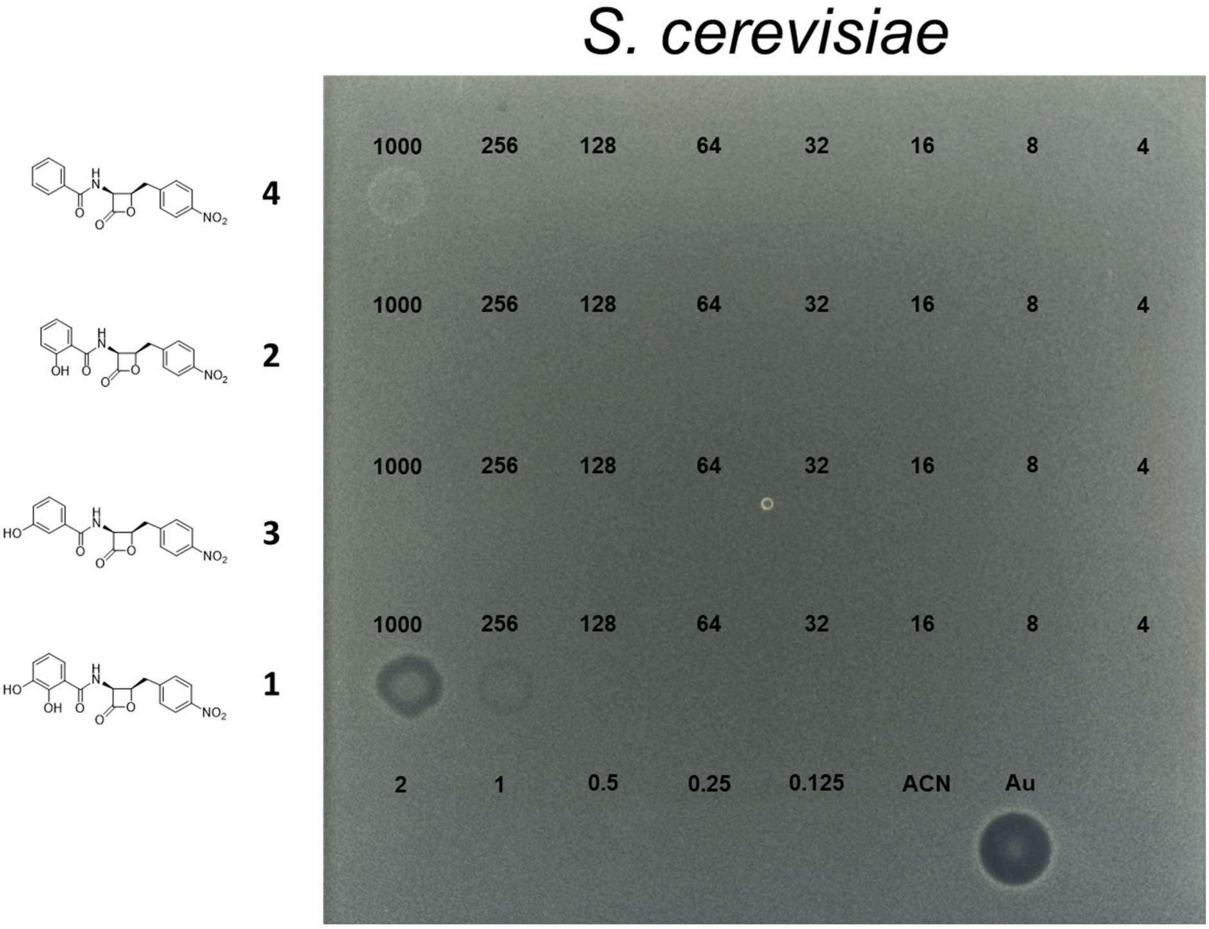

Figure S12: Modification of the 1-catechol abolishes antifungal activity against *S. cerevisiae*. Spot-on-lawn bioassay of **1**, **2**, **3** and **4** with *S. cerevisiae,* with zones of clearing indicating growth inhibition. Numbers indicate concentration of **1, 2, 3** or **4** in µg/mL. Minimum inhibitory concentrations are **1**: 1 mg/mL; **2**, **3** and **4**: > 1 mg/mL. Acetonitrile (ACN) and aureobasidin A (Au) (0.2 µg/mL) were used as negative and positive controls respectively.

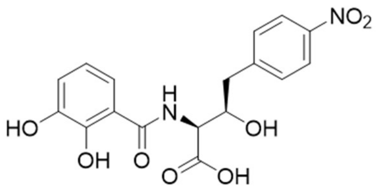

5

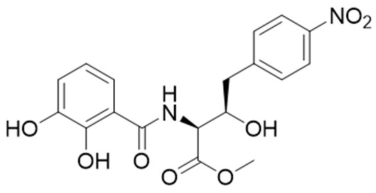

6

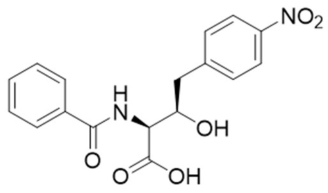

7

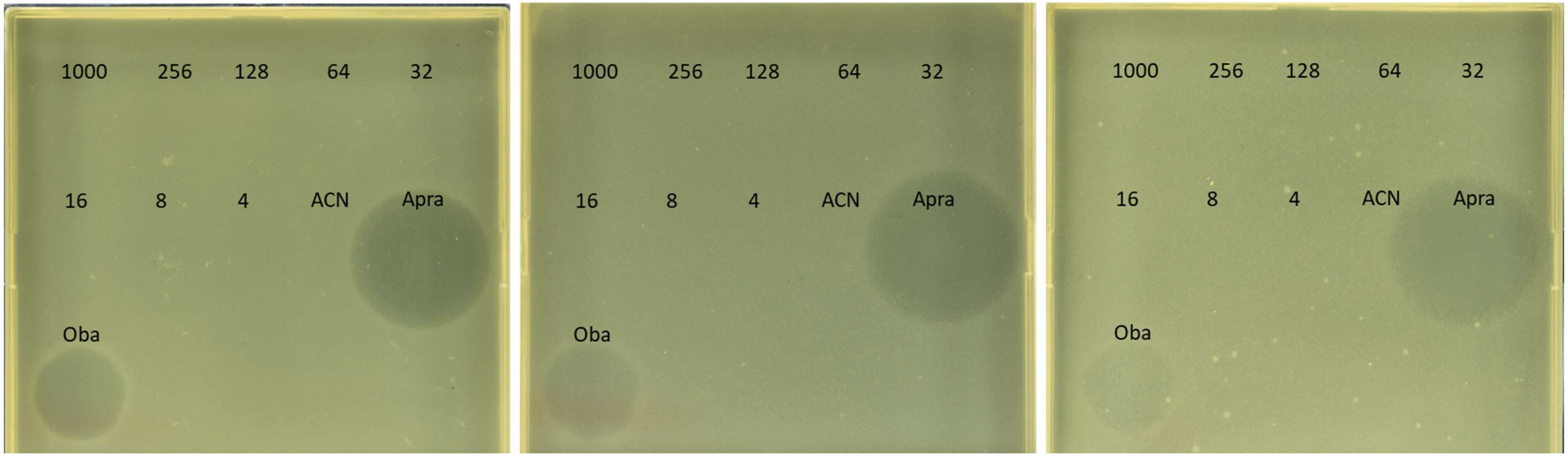

Figure S13: Ring-open 1-analogues lack antibacterial activity. Spot-on-lawn bioassays of ring-open analogues **5**, **6** and **7** dissolved in acetonitrile (ACN) against MRSA, with zones of clearing indicating growth inhibition. Numbers indicate concentration of **5**, **6** and **7** in µg/mL. ACN was used as a negative control and apramycin (Apra) (50 µg/mL) and obafluorin (Oba) (64 µg/mL) were used as positive controls.

PfThrRS aminoacylation assays

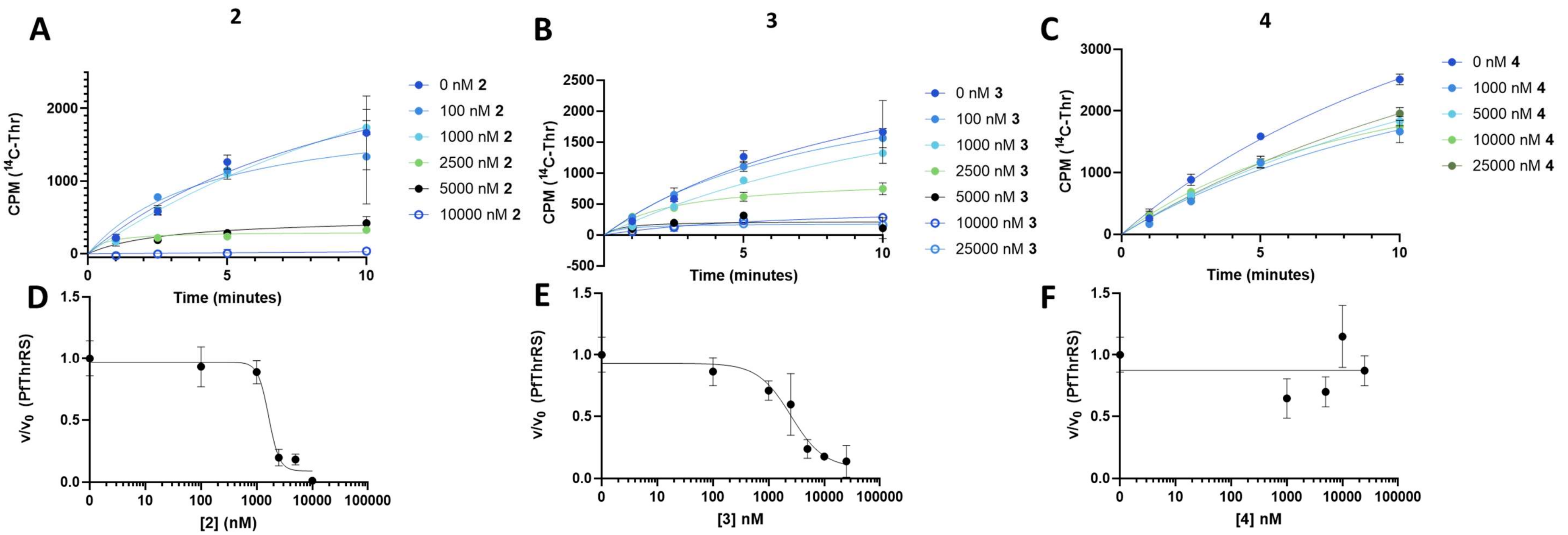

Figure S14: The catechol moiety of 1 is required for inhibition of PfThrRS. Progress curves for PfThrRS in the presence of varying (0–25 µM) concentrations of **2** (**A**), **3** (**B**) and **4** (**C**). Reactions (n = 3) included enzyme at 10 nM, which was preincubated with compound for 10 min prior to the addition of saturating concentrations of tRNA, threonine, and ATP. Compounds **2** and **3** fully inhibited PfThrRS only at 10000 nM and 5000 nM respectively, in comparison to 10 nM for **1** (Figure 5), whereas compound **4** showed no significant inhibition of PfThrRS up to 25000 nM. The linear portions of the progress curves were fit to a linear equation to derive initial rates. Error bars represent the standard error for each time point. Dose response curves for PfThrRS with compound **2** (**D**), **3** (**E**) and **4** (**F**) were calculated from the data in (**A**), (**B**) and (**C**) respectively by plotting the fractional velocity (v/v_0) at each measured inhibitor concentration against log [compound]. IC_{50} values for compounds **2** and **3** are 1600 ± 300 nM and 2500 ± 800 nM, respectively. PfThrRS was not inhibited by compound **4**. Details of the fitting routines are presented in the Methods. CPM = counts per min.

Bioassays with modified iron concentrations

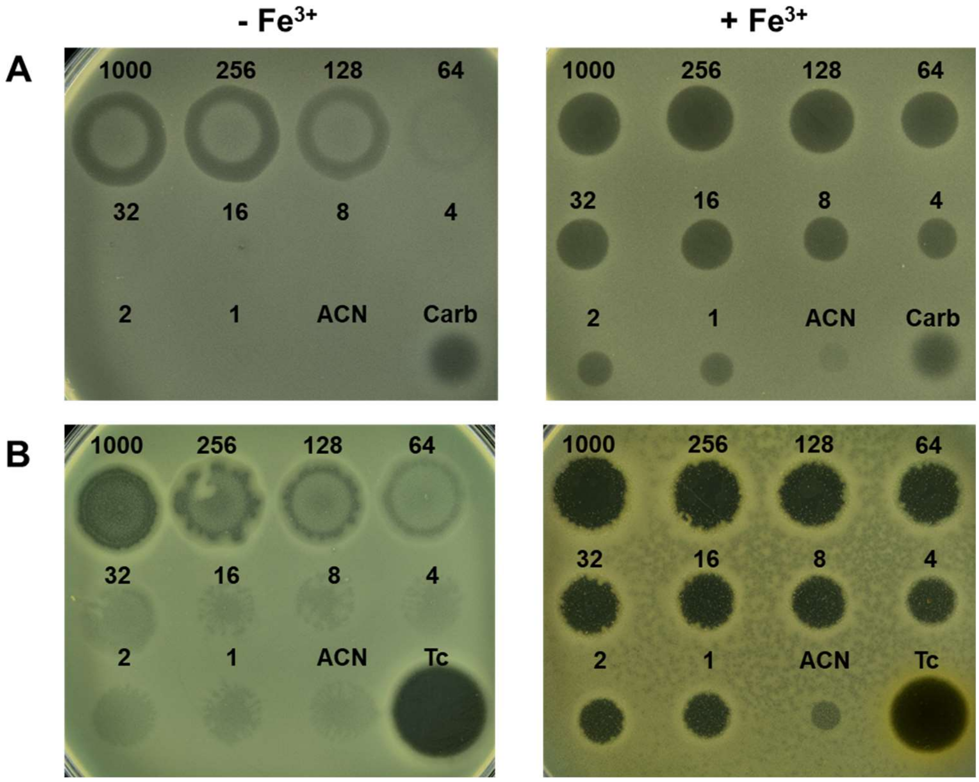

Figure S15: The presence of additional Fe^{3+} increases the bioactivity of 1 against Gram-negative strains. Spot-on-lawn bioassays of **1** against A) *E. coli* 25922 in the absence and presence of Fe^{3+} (2 mM). B) *P. aeruginosa* PA01 in the absence and presence of Fe^{3+} (1.5 mM). Concentrations of Fe^{3+} are the maximum tolerated by each organism before inhibition of growth was observed. Numbers indicate concentrations of **1** (in μg/mL). Acetonitrile (ACN) was used as a negative control and carbenicillin (Carb; 1000 μg/mL) or tetracycline (Tc; 1000 μg/mL), were used as the positive controls for *E. coli* 25922 or *P. aeruginosa* respectively. MICs are altered from 256 to ≤ 1 μg/mL and 128 to ≤ 1 μg/mL for *E. coli* 25922 and *P. aeruginosa* PA01 respectively.

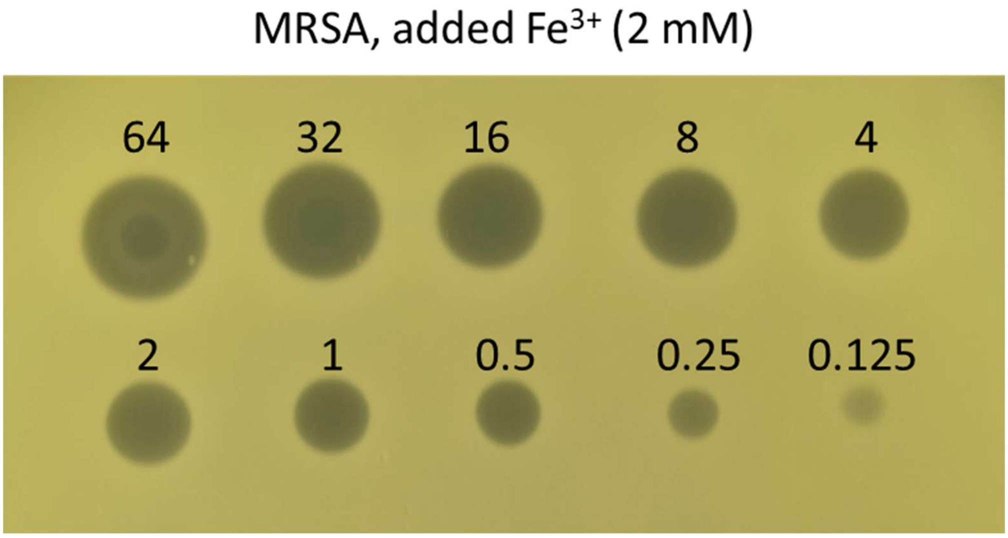

Figure S16: The presence of additional Fe^{3+} decreases the MIC of 1 against MRSA. Spot-on-lawn bioassay of **1** against MRSA with added Fe^{3+} (2 mM). Comparison with Figure S8 shows a reduction in the MIC from 2 to 0.25 µg/mL. Numbers indicate concentrations of **1** (in µg/mL) dissolved in acetonitrile (ACN).

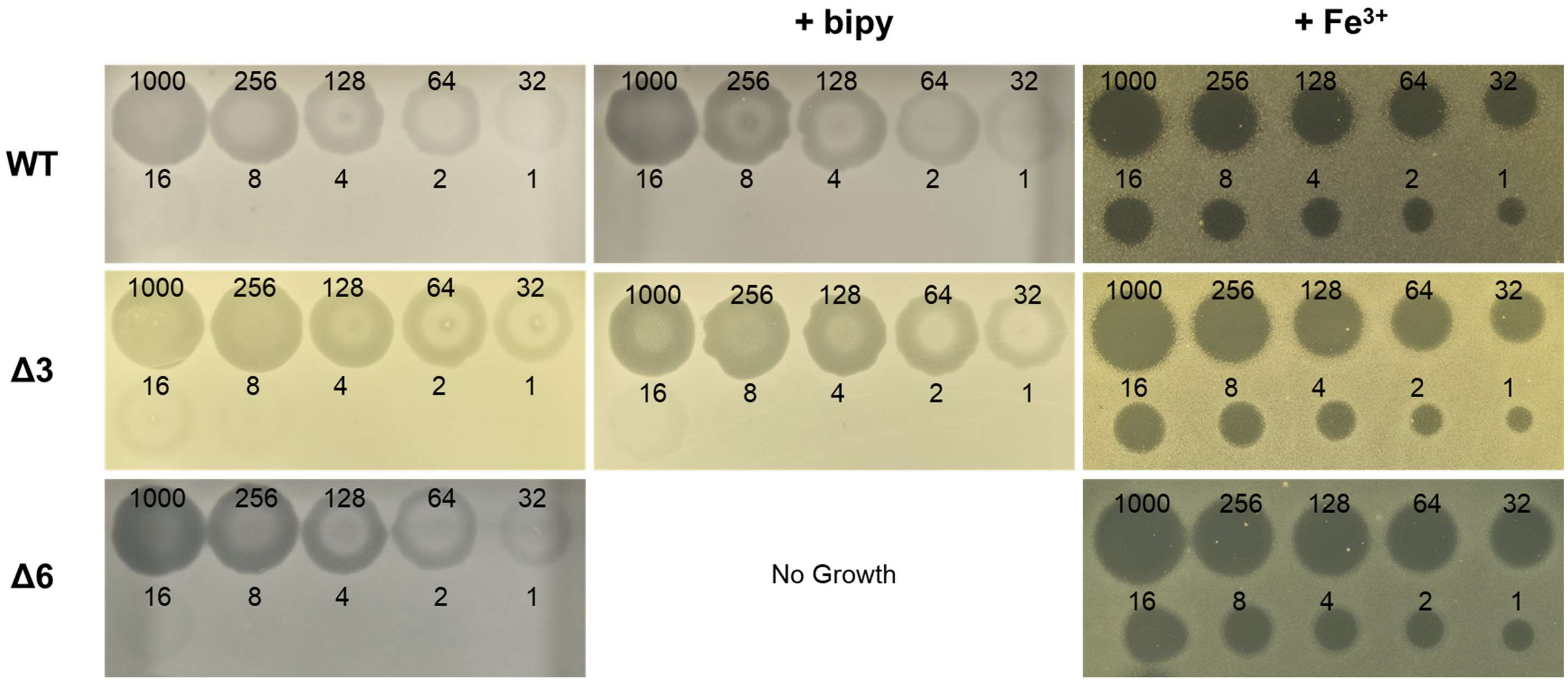

Figure S17: No active uptake of 1 via TonB-dependent transporters. Spot-on-lawn bioassays of **1** against *E. coli* BW25113 WT and the TonB dependent transporter (TBDT) knock-out mutants, Δ3 = Δ*fhu*AΔ*fecA*Δ*cirA* and Δ6 = Δ*fhuA*Δ*fecA*Δ*cirA*Δ*fepA*Δ*fhuE*Δ*fiu*. The minimum inhibitory concentration (MIC) of 256 µg/mL for the WT and mutant strains is unchanged in the presence of the Fe^{3+} chelator bipy (150 µM), demonstrating there is no active uptake of **1** via TBDTs. *E. coli* BW25113 Δ6 was unable to grow in iron deplete conditions. As for *E. coli* 25922 (Figure S16) the MIC is reduced to ≤ 1 µg/mL in the presence of Fe^{3+} (2 mM).

Table S1: Changes to antibacterial activity of antibiotics with additional Fe^{3+}. The lowest concentrations (μg/mL) of a range of antibiotics which gave an inhibition zone against *S. aureus* and *E. coli* 25922 in bioassay conditions, with and without additional Fe^{3+} (2 mM).

**Antibiotic**	***S. aureus***		***E. coli* 25922**	
	**- Fe^{3+}**	**+ Fe^{3+}**	**- Fe^{3+}**	**+ Fe^{3+}**
Carbenicillin	50	50	1000	1000
Kanamycin	50	500	50	250
Streptomycin	50	500	50	100
Nitrofurantoin	500	250	500	100
Chloramphenicol	1000	250	1000	500
Apramycin	50	1000	50	1000

Hydrolysis of analogues 2-4 in the absence and presence of Fe^{3+}

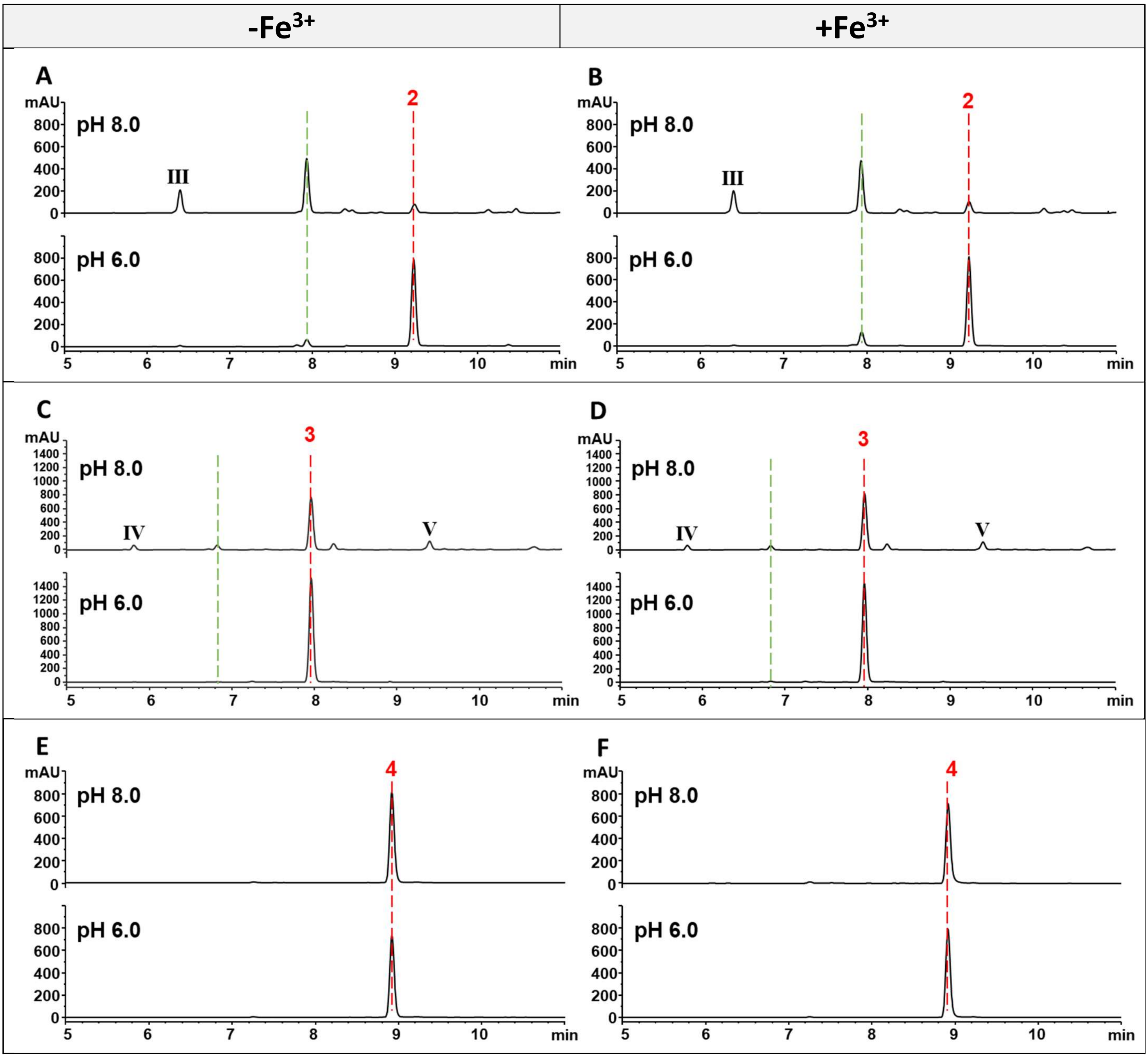

Figure S18: Fe^{3+} does not have a protective effect against hydrolysis of analogues 2-4. UV chromatograms at 270 nm recorded 30 min after the mixing of solutions of **A**) **2** (1mM) in HEPES buffer (100 mM) at pH 6.0 and 8.0, **B**) **2** (1 mM) in HEPES buffer (100 mM) with added Fe^{3+} (1 mM) at pH 6.0 and 8.0; **C**) **3** (1mM) in HEPES buffer (100 mM) at pH 6.0 and 8.0, **D**) **3** (1 mM) in HEPES buffer (100 mM) with added Fe^{3+} (1 mM) at pH 6.0 and 8.0; **E**) **4** (1mM) in HEPES buffer (100 mM) at pH 6.0 and 8.0, **F**) **4** (1 mM) in HEPES buffer (100 mM) with added Fe^{3+} (1 mM) at pH 6.0 and 8.0. The red lines indicate the intact obafluorin analogue (**2-4** respectively) and the green lines indicate the hydrolysed analogue. The side products **III** and **IV** are proposed to be adducts of HEPES and the obafluorin analogue, and **V** is proposed to be a dimer of **3** (Figure S19).

I

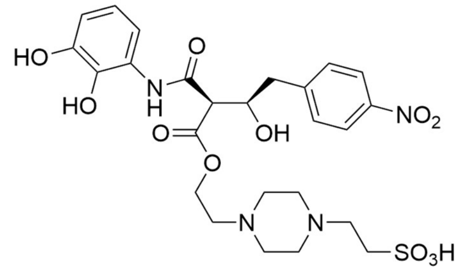

Chemical Formula: $C_{25}H_{32}N_4O_{11}S$
Calculated $[M+H]^+$ = 597.1861
Found $[M+H]^+$ = 597.1867, Δ = 1.0 ppm

II

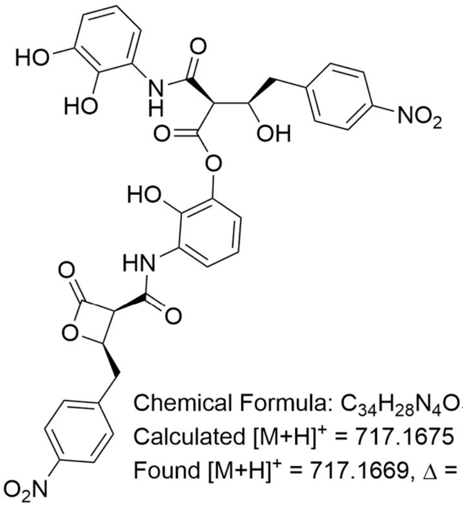

Chemical Formula: $C_{34}H_{28}N_4O_{14}$
Calculated $[M+H]^+$ = 717.1675
Found $[M+H]^+$ = 717.1669, Δ = 1.0 ppm

III

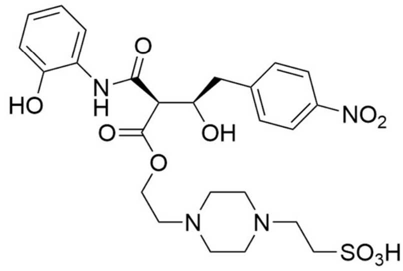

Chemical Formula: $C_{25}H_{32}N_4O_{10}S$
Calculated $[M+H]^+$ = 581.1912
Found $[M+H]^+$ = 581.1911, Δ = -0.2 ppm

IV

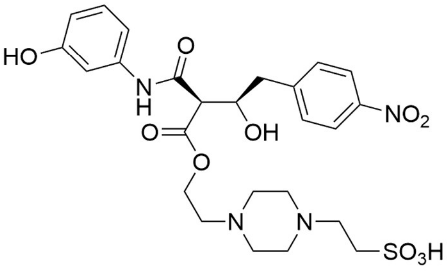

Chemical Formula: $C_{25}H_{32}N_4O_{10}S$
Calculated $[M+H]^+$ = 581.1912
Found $[M+H]^+$ = 581.1906, Δ = -1.0 ppm

V

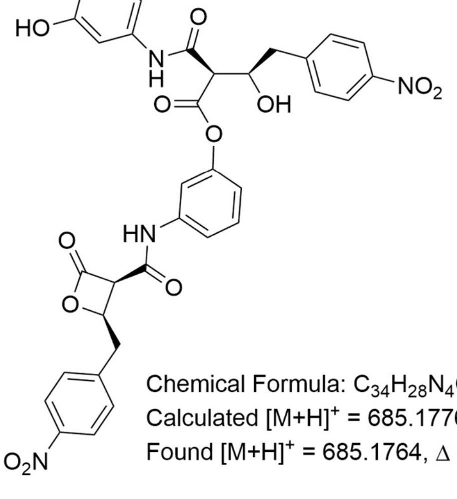

Chemical Formula: $C_{34}H_{28}N_4O_{12}$
Calculated $[M+H]^+$ = 685.1776
Found $[M+H]^+$ = 685.1764, Δ = -1.8 ppm

Figure S19: Proposed structures of side products seen in HPLC analysis of hydrolysis experiments. The side products **I**, **III** and **IV** are proposed to be adducts from the nucleophilic attack of **1** and its analogues (**2** and **3**) by HEPES buffer, and **II** and **V** are proposed to be a dimers of **1** and **3** respectively. We propose dimerization of **1** via 3-OH to give **II** because the calculated pKa of 2-OH and 3-OH are 8.3 and 11.9 respectively.* The higher pKa of 3-OH means it is a stronger conjugate base, and thus we'd expect higher electron density on this oxygen, making it the better nucleophile.

* Marvin was used for calculating the pKa values, Marvin 19.3.0, Chemaxon (https://www.chemaxon.com)

Tabulated NMR and spectra

NMR spectra were recorded on a Bruker AVANCE III 400 MHz spectrometer at 298 K. Chemical shifts are reported in parts per million (ppm) relative to the solvent residual peak of acetone (^{1}H: 2.05 ppm, quintet; ^{13}C: 29.92 ppm, septet).

Obafluorin (1)

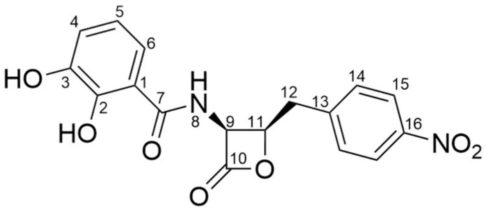

Table S2: Resonances assignment in ^{1}H and ^{13}C NMR spectra of **1** in acetone-d_6 at 298 K.

Position	δ_H (no. of protons, multiplicity, *J* in Hz)	δ_c	COSY	HMBC
1	9.05 (1H, d, 4.84)	114.8		
2		150.7		
3		147.4		
4	7.37 (1H, d, 7.5)	118.2	5	2, 6, 7
5	6.84 (1H, dd, 7.5 & 7.9)	119.8	4, 6	1, 3
6	7.05 (1H, d, 7.9)	120.4	5	2, 3, 4
7		171.4		
8	9.05 (1H, d, 4.7)	-	9	
9	6.05-6.02 (1H, m)	59.9	8, 11	10
10		168.8		
11	5.24-5.19 (1H, m)	78.3	9, 12	10
12	3.54 (1H, dd, 14.7 & 9.2)<br>3.41 (1H, dd, 14.7 & 4.4)	36.1	9, 11	9, 12, 13, 14
13		145.5		
14	7.60 (2H, d, 8.5)	131.2	15	12, 14, 15, 16
15	8.16 (2H, d, 8.5)	124.4	14	13, 15, 16
16		147.9		
2-OH	11.93 (1H, s)			2, 3, 6
3-OH	8.16 (1H, m)			

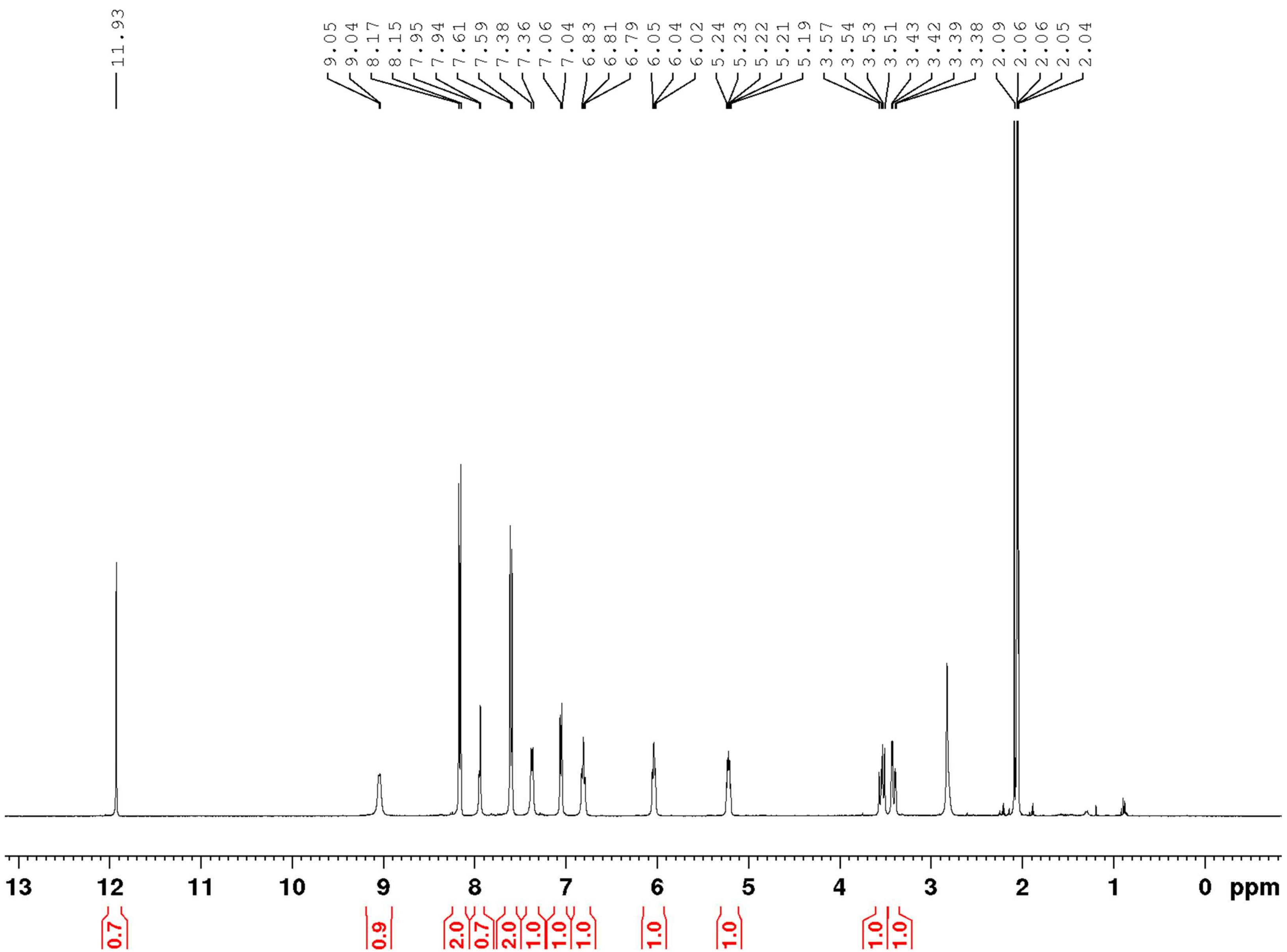

Figure S20: ^{1}H NMR of 1 in acetone-d_6 at 298 K.

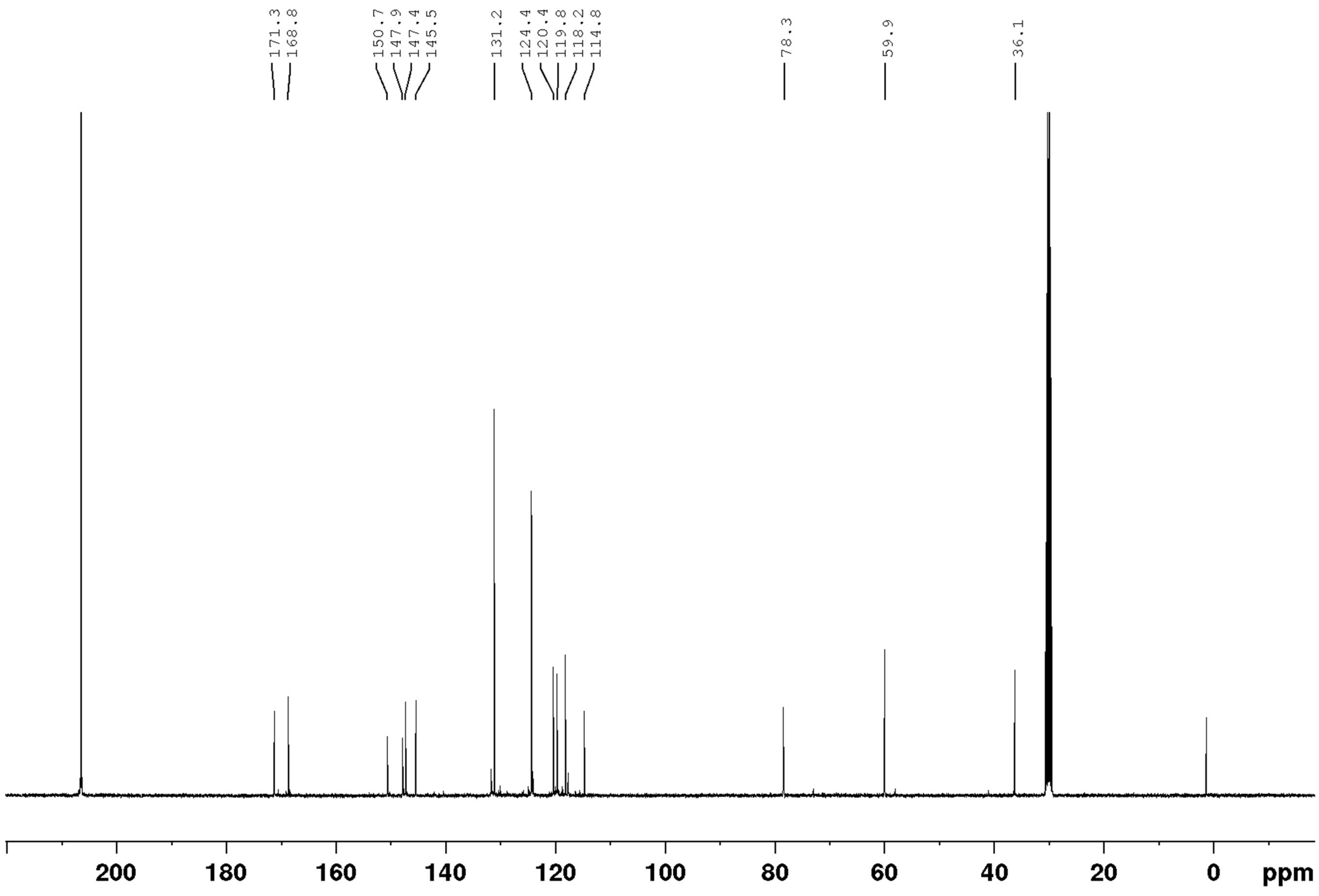

Figure S21: ^{13}C NMR of **1** in acetone-d_6 at 298 K.

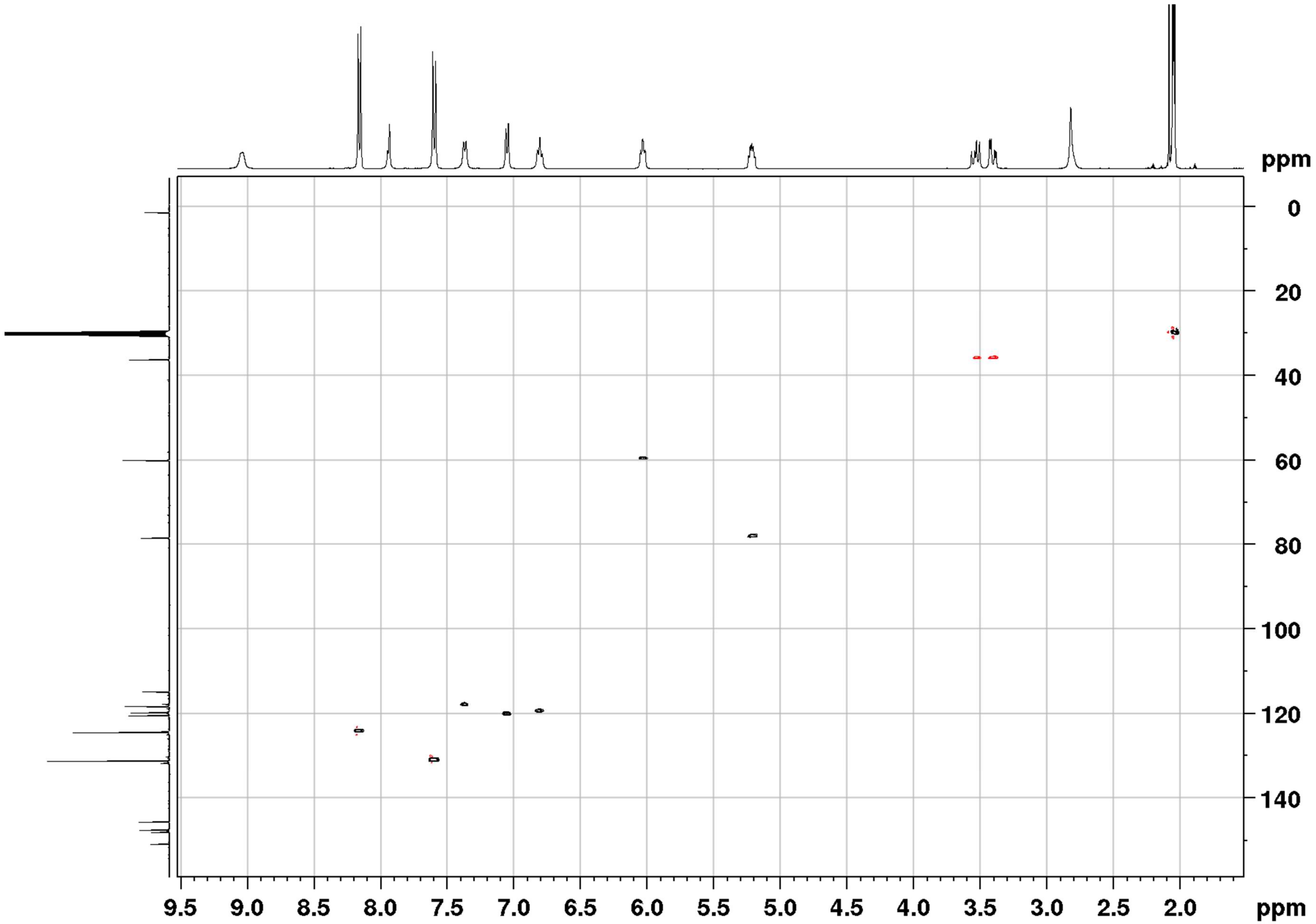

Figure S22: HSQC-edited NMR of **1** in acetone-d_6 at 298 K.

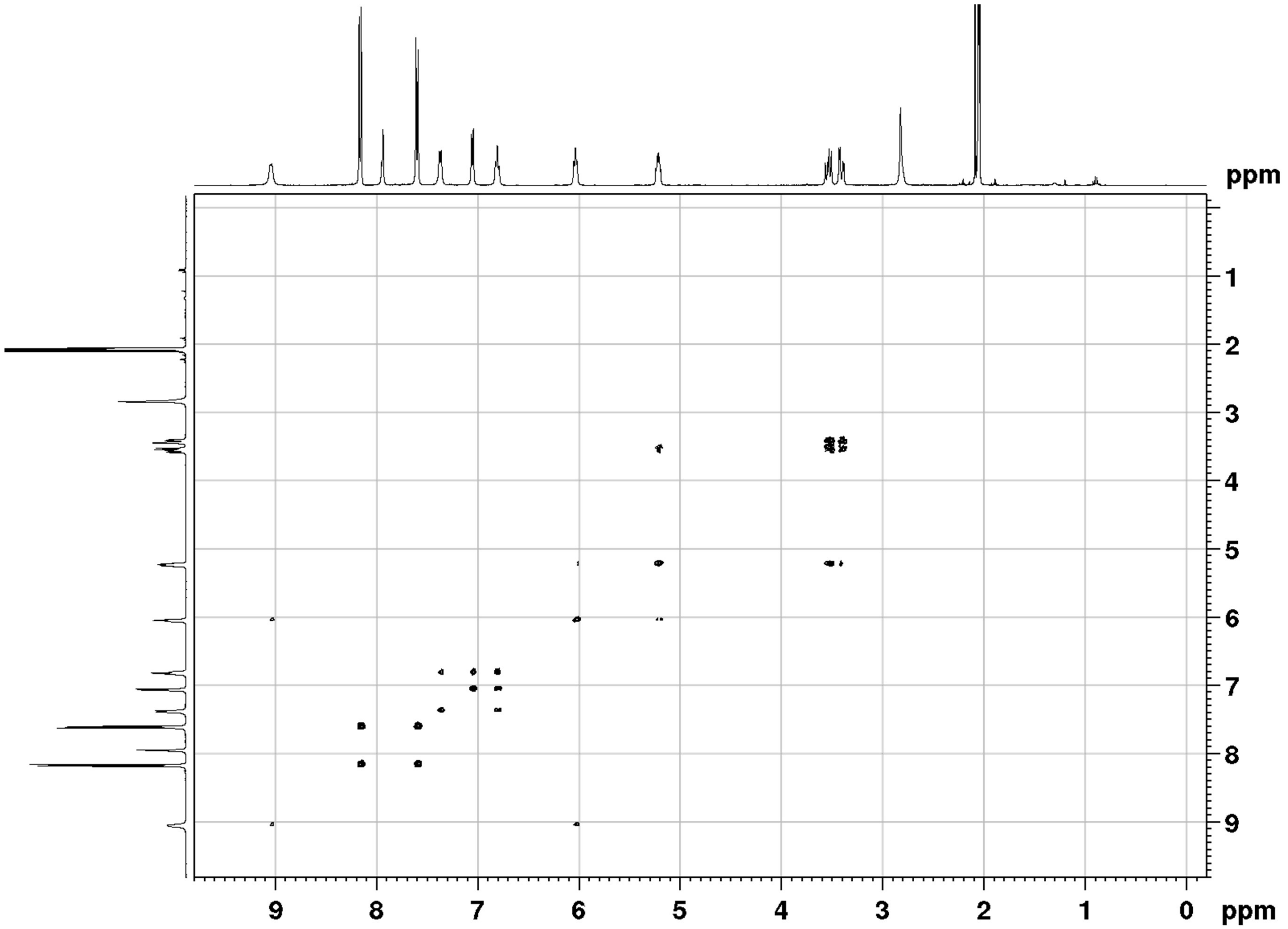

Figure S23: COSY NMR of **1** in acetone-d_6 at 298 K.

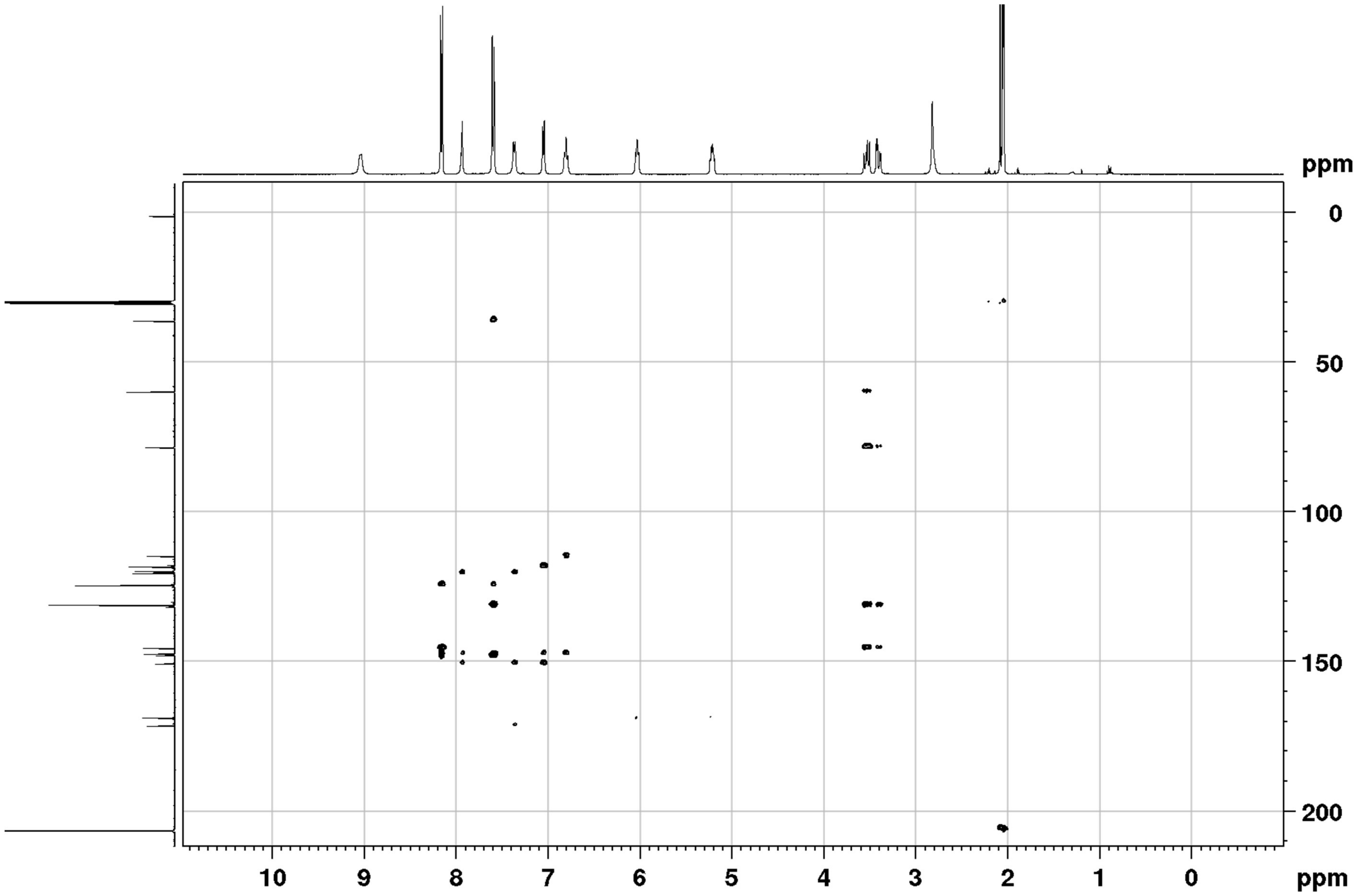

Figure S24: HMBC NMR of **1** in acetone-d_6 at 298 K.

2-HBA-obafluorin (2)

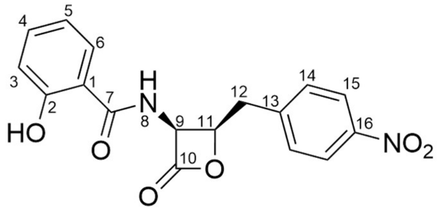

Table S3: Resonances assignment in ^{1}H and ^{13}C NMR spectra of **2** in acetone-d_6 at 298 K.

Position	δ_H (no. of protons, multiplicity, *J* in Hz)	δ_c	COSY	HMBC
1		114.8		
2		162.2		
3	6.92-6.92 (1H, m)*	118.9	4, 5, 6	1, 2 (weak), 5
4	7.52-7.46 (1H, m)	135.8	3, 5	2, 6
5	6.92-6.92 (1H, m)*	120.0	3, 4, 6	1, 3, 6 (weak)
6	7.90-7.86 (1H, m)	128.3	3, 5	2, 4, 7
7		170.9		
8	9.07 (1H, d, 8.2)			
9	6.07-6.02 (1H, m)	59.8	8, 11	7, 10, 11
10		168.9		
11	5.26-5.19 (1H, m)	78.4	9, 12	10
12	3.54 (1H, dd, 14.8 & 9.3)<br>3.40 (1H, dd, 14.8 & 4.7)	36.2	11, 12	9, 11, 13, 14
13		145.6		
14	7.60 (2H, d, 8.8)	131.2	15	12, 14, 16
15	8.16 (2H, d, 8.8)	124.4	14	13, 15, 16
16		148.0		
2-OH	11.78 (1H, s)			

*H-3 and H-5 are under the same signal.

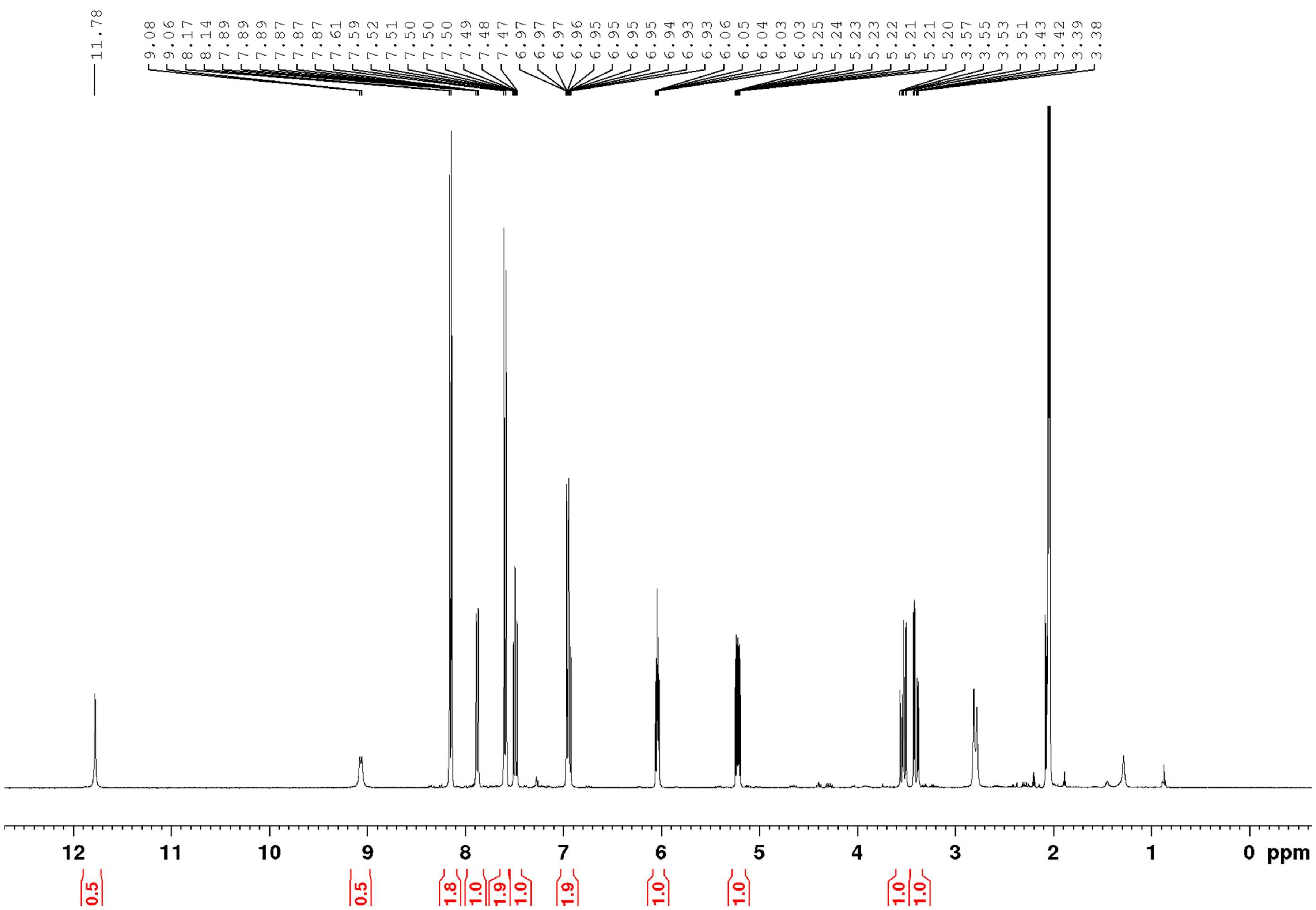

Figure S25: ^{1}H NMR of **2** in acetone-d_6 at 298 K.

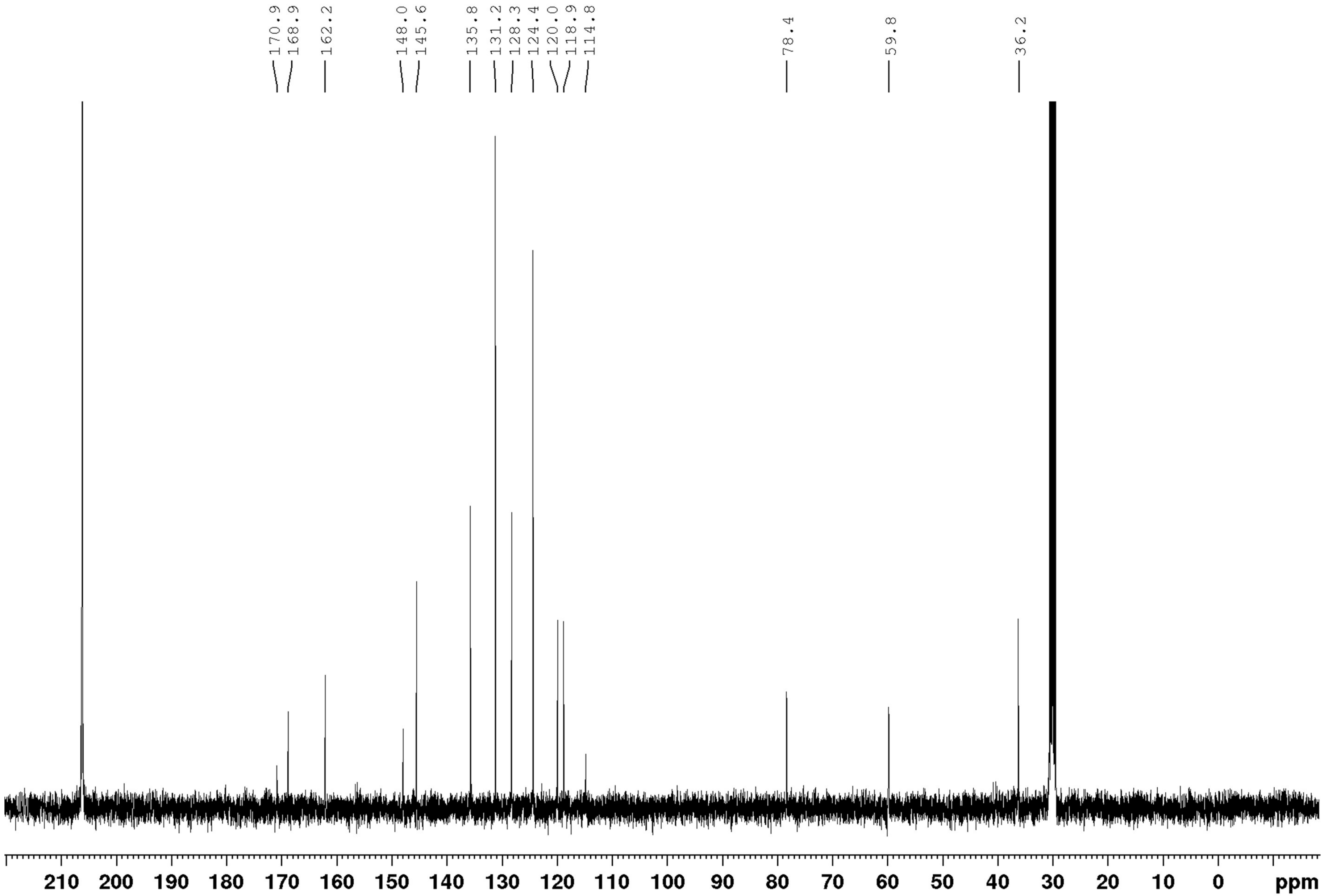

Figure S26: ^{13}C NMR for **2** in acetone-d_6 at 298 K.

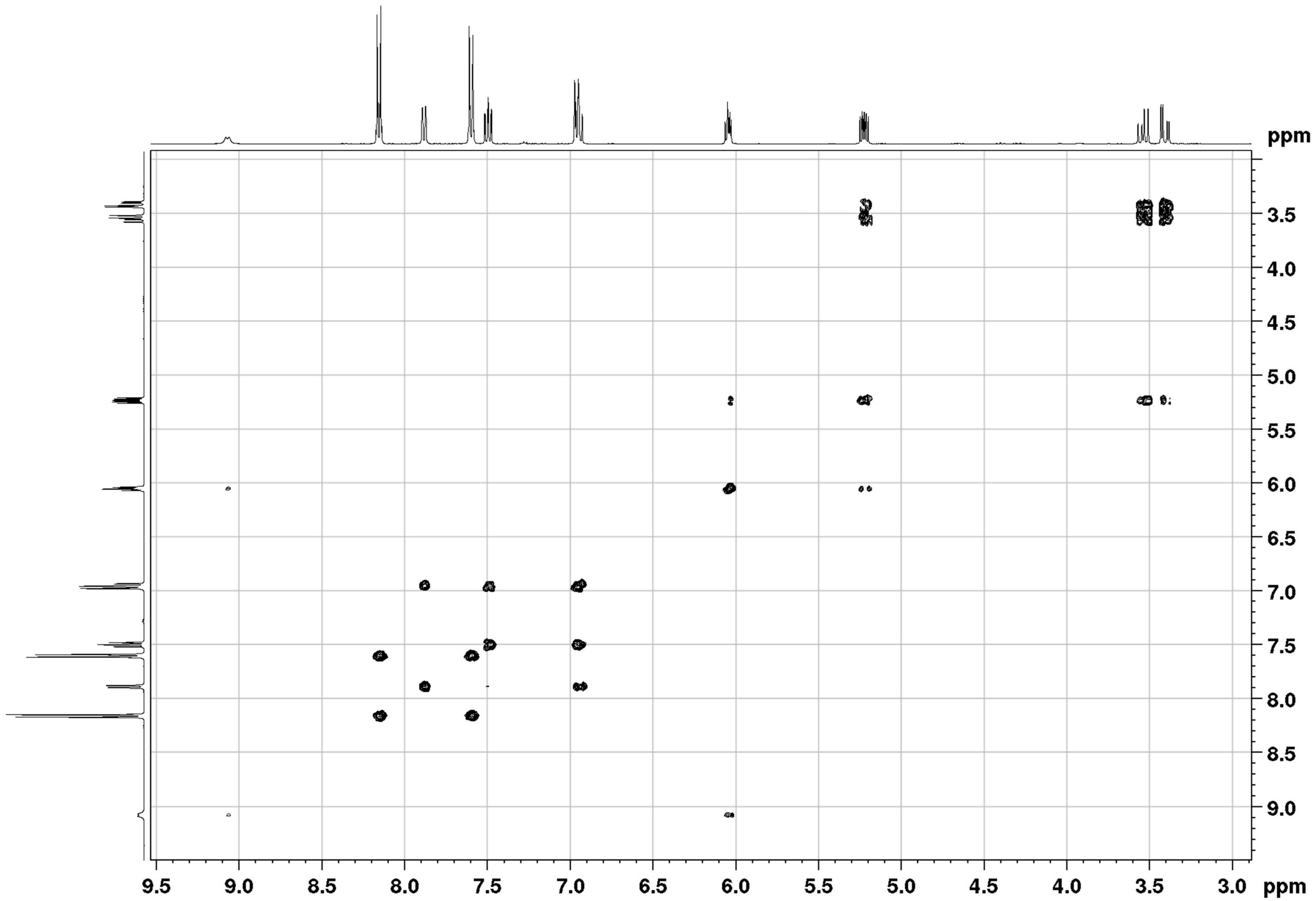

Figure S27: COSY NMR of **2** in acetone-d_6 at 298 K.

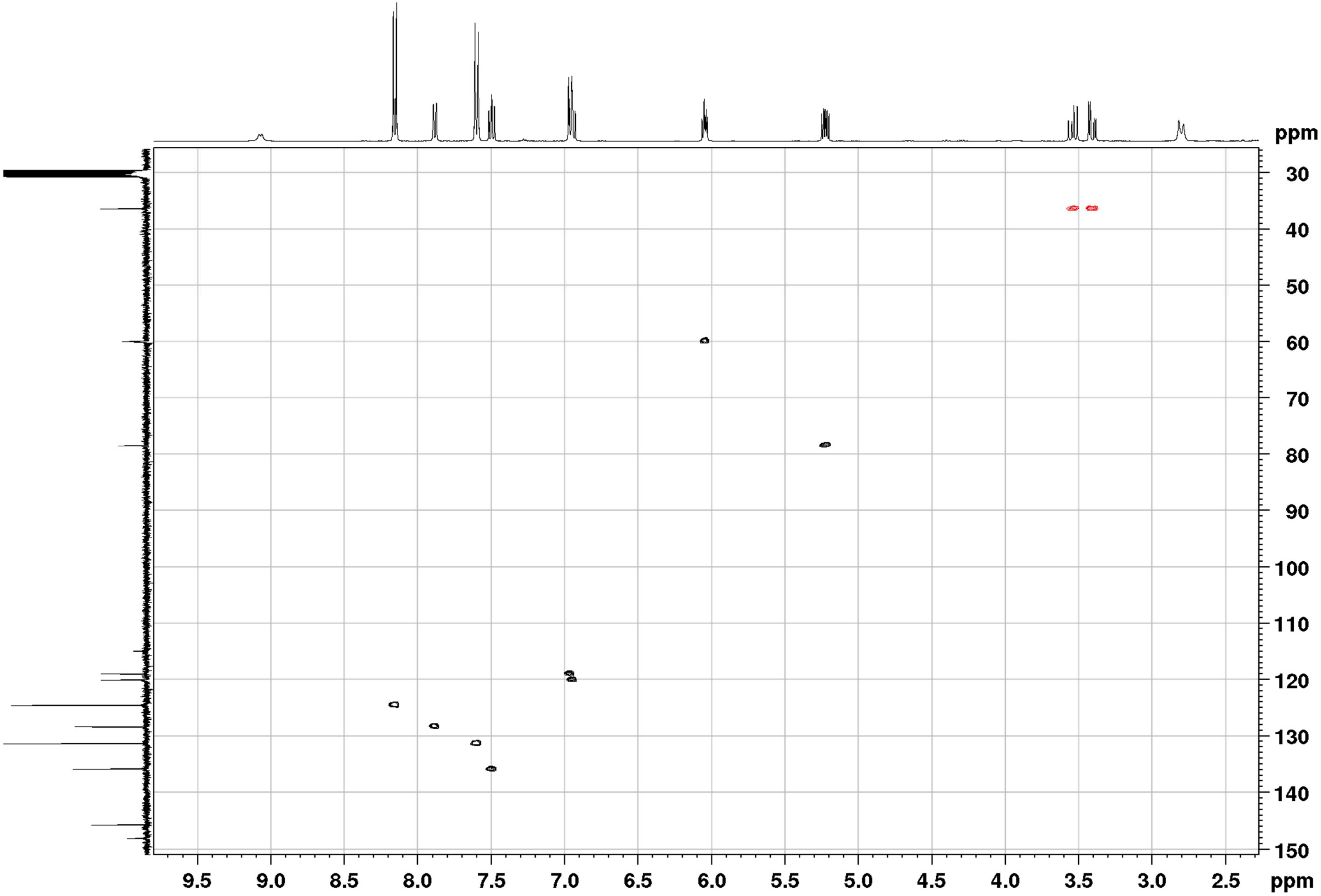

Figure S28: HSQC-edited NMR of **2** in acetone-d_6 at 298 K.

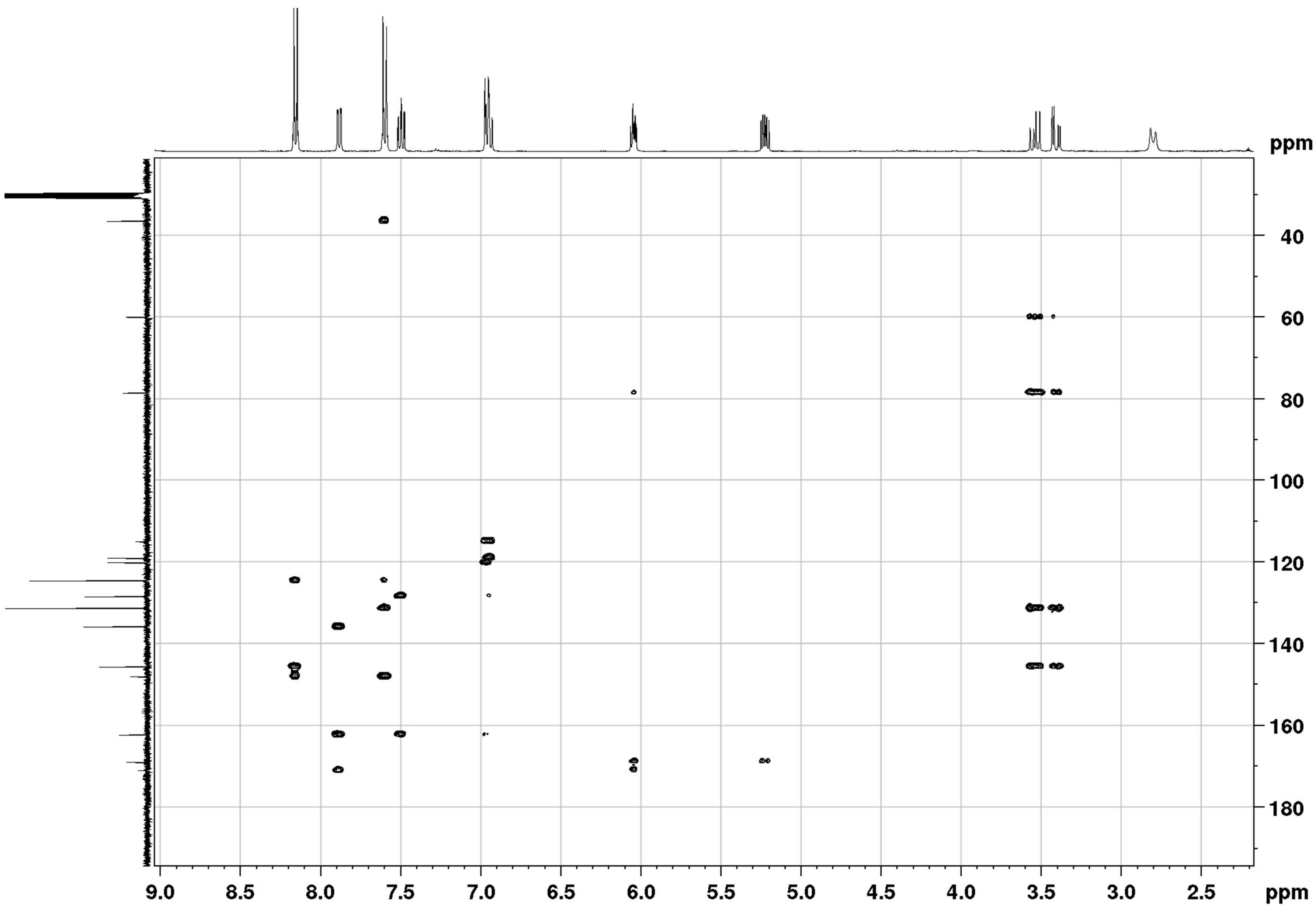

Figure S29: HMBC NMR of **2** in acetone-d_6 at 298 K.

3-HBA-obafluorin (3)

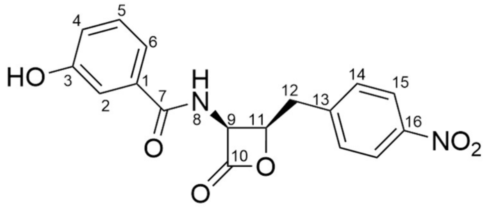

Table S4: Resonances assignment in ^{1}H and ^{13}C NMR spectra of **3** in acetone-d_6 at 298 K.

Position	δ_H (no. of protons, multiplicity, *J* in Hz)	δ_c	COSY	HMBC
1		135.7		
2	7.42-7.39 (1H, m)**	115.4	4, 5, 6	3, 4, 6, 7
3		158.6		
4	7.06 (1H, ddd, 8.1 & 2.5 & 1.1)	120.0	2, 5, 6	2, 6
5	7.33 (1H, dd, 8.1 & 8.1)	130.7	2, 4, 6	1, 3
6	7.42-7.39 (1H, m)**	119.3	2, 4, 5	2, 3, 4, 7
7		167.6		
8	8.70-8.65 (1H, m)*		9	
9	6.00-5.96 (1H, m)	60.4	8, 11	7, 10
10		169.5		
11	5.19-5.12 (1H, m)	78.5	9, 12	10
12	3.52 (1H, dd, 14.9 & 9.2)<br>3.38 (1H, dd, 14.9 & 4.6)	36.2	11, 12	9, 12, 13, 14
13		145.9		
14	7.60 (2H, d, 8.8)	131.3	15	12, 13, 14, 15
15	8.17 (2H, d, 8.8)	124.5	14	13, 15, 16
16		148.0		
3-OH	8.70-8.65 (1H, m)*			

* H-8 and 3-O<u>H</u> are both under the same signal

** H-6 and H-2 are both under the same signal

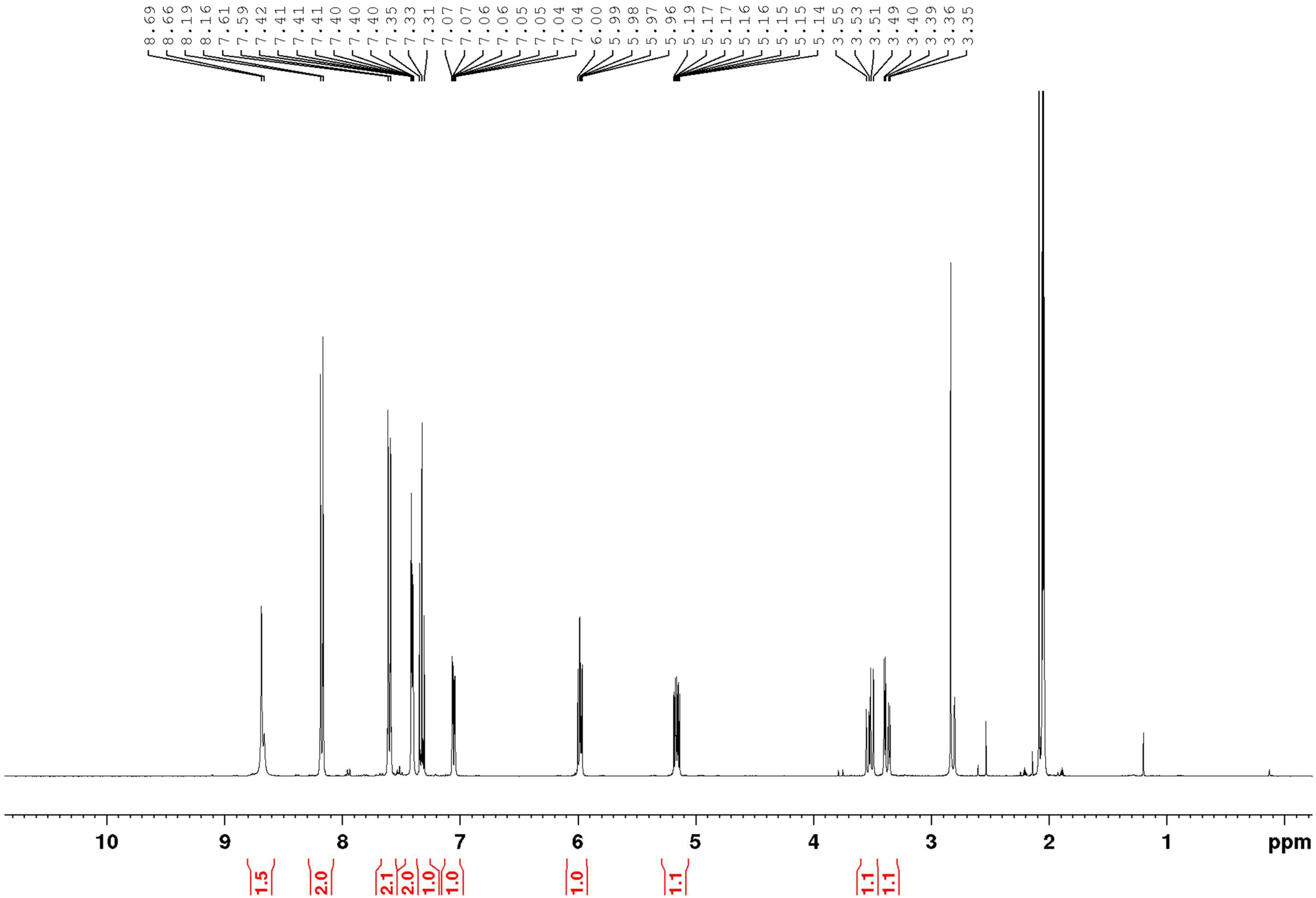

Figure S30: ^{1}H NMR of **3** in acetone-d_6 at 298 K.

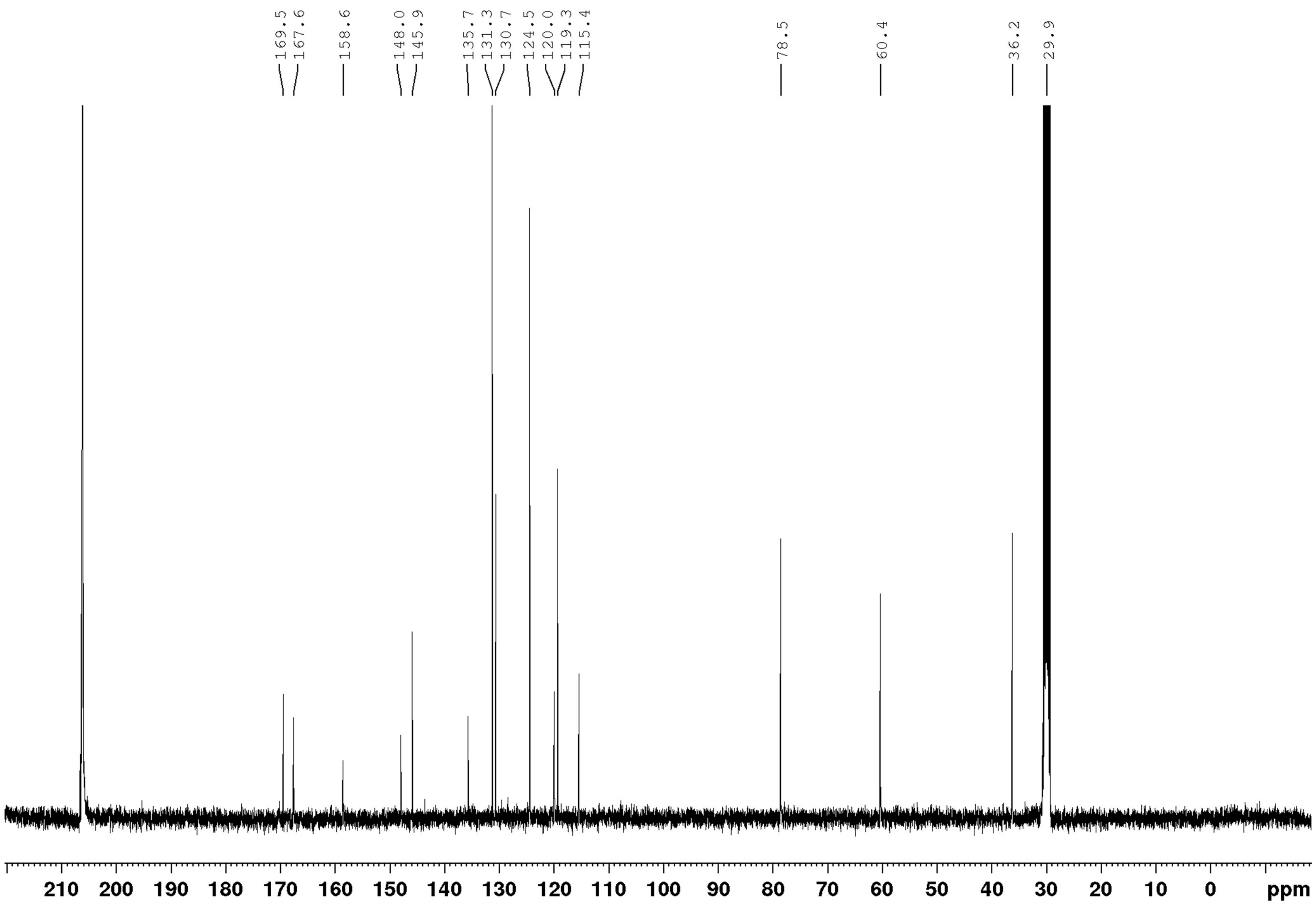

Figure S31: ^{13}C NMR of **3** in acetone-d_6 at 298 K.

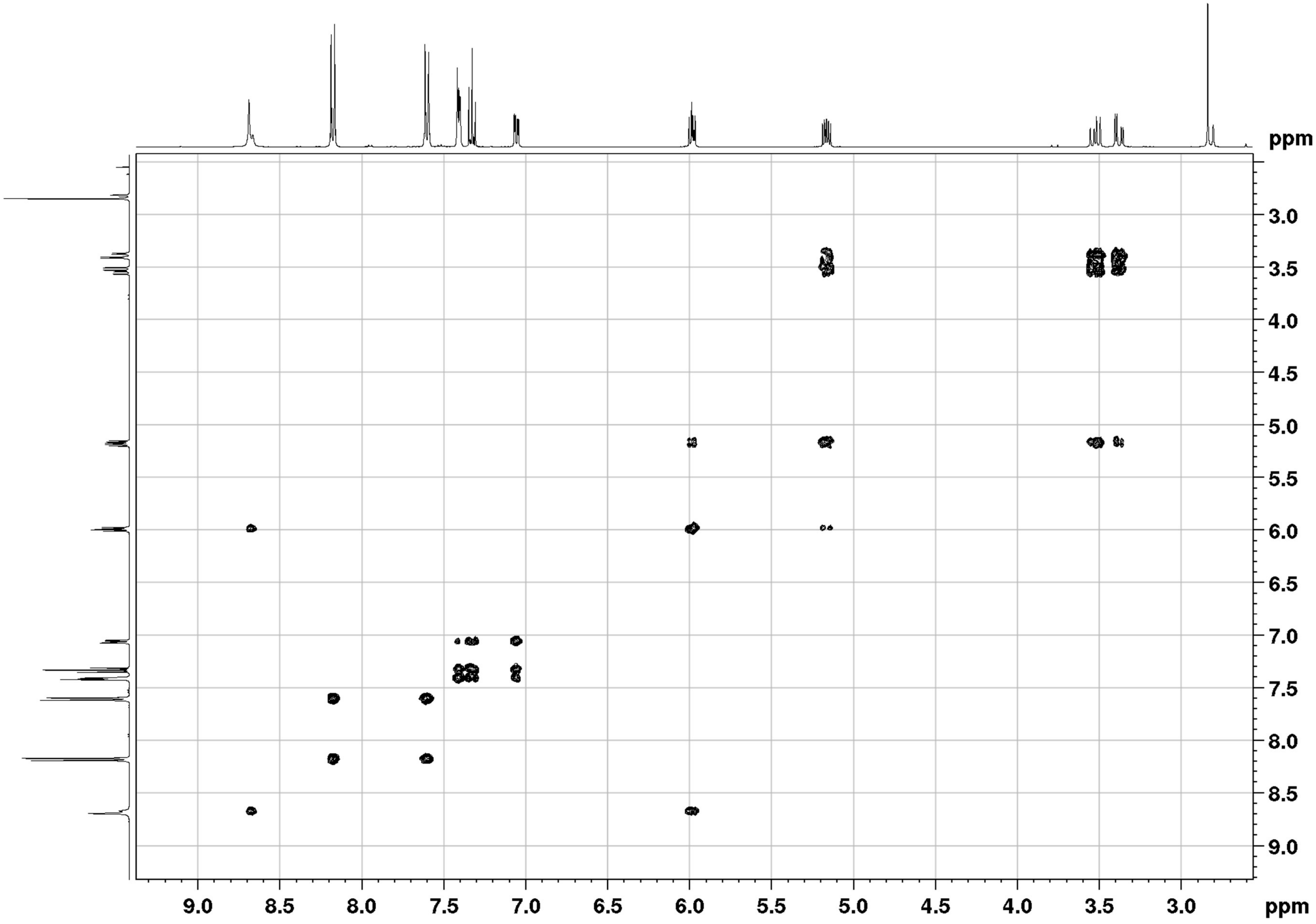

Figure S32: COSY NMR of **3** in acetone-d_6 at 298 K.

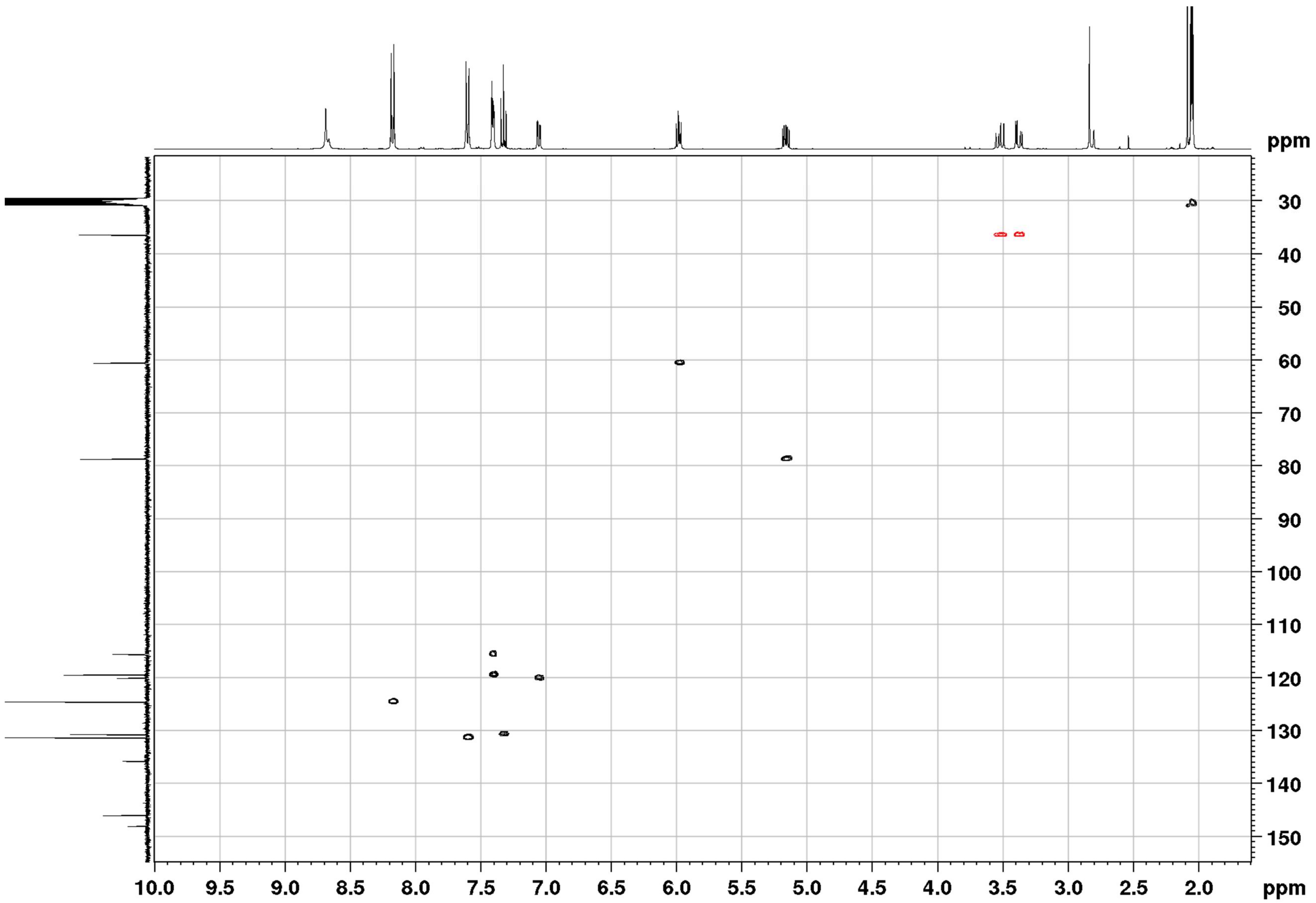

Figure S33: HSQC-edited NMR of **3** in acetone-d_6 at 298 K.

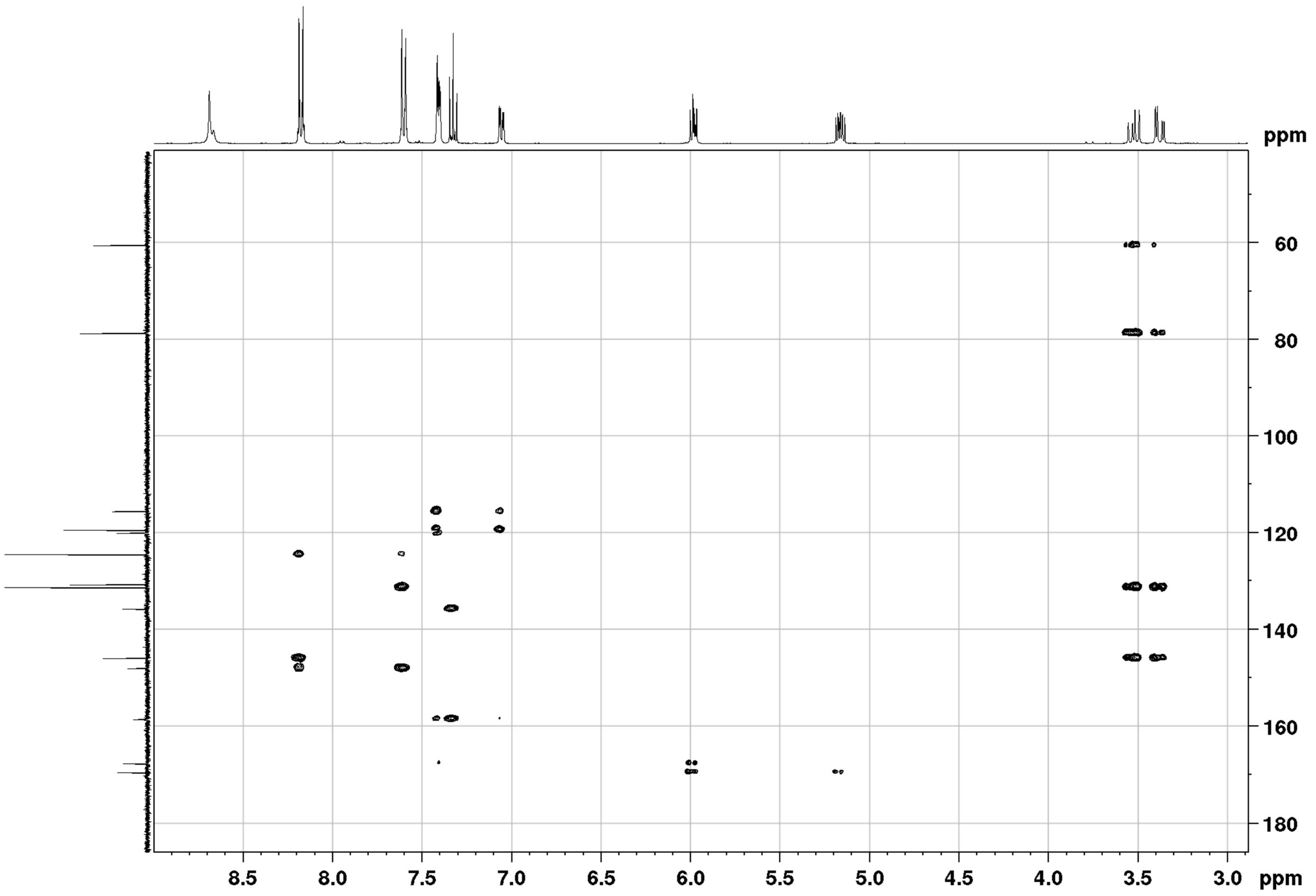

Figure S34: HMBC of **3** in acetone-d_6 at 298 K.

BA-obafluorin (4)

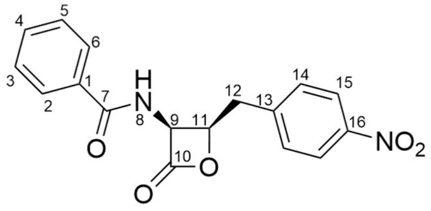

Table S5: Resonances assignment in ^{1}H and ^{13}C NMR spectra of **4** in acetone-d_6 at 298 K.

Position	δ_H (no. of protons, multiplicity, *J* in Hz)	δ_C	COSY ^{1}H-^{1}H	HMBC ^{1}H-^{13}C
1		134.2		
2/6	7.95 (2H, d, 7.3 Hz)	128.4	3/5	2/6, 4, 7
3/5	7.52 (2H, dd, 7.3, 7.6)	129.5	2/6, 4	1, 3/5
4	7.62-7.58 (1H, m)*	133.0	3/5	2/6, 14
7		167.6		
8	8.75 (1H, d, 8.1)		9	
9	6.03-5.96 (1H, m)	60.3	8, 11	7, 10, 11
10		169.4		10
11	5.20-5.15 (1H, m)	78.5	9, 12	
12	3.53 (1H, dd, 15.0 & 9.4)<br>3.38 (1H, dd, 15.0 & 4.5)	36.2	11, 12	9, 11, 13, 14
13		145.9		
14	7.60 (2H, d, 8.7)	131.3	15	12, 16
15	8.17 (2H, d, 8.7)	124.4	14	13, 15, 16
16		148.0		

*under the signal for H-14

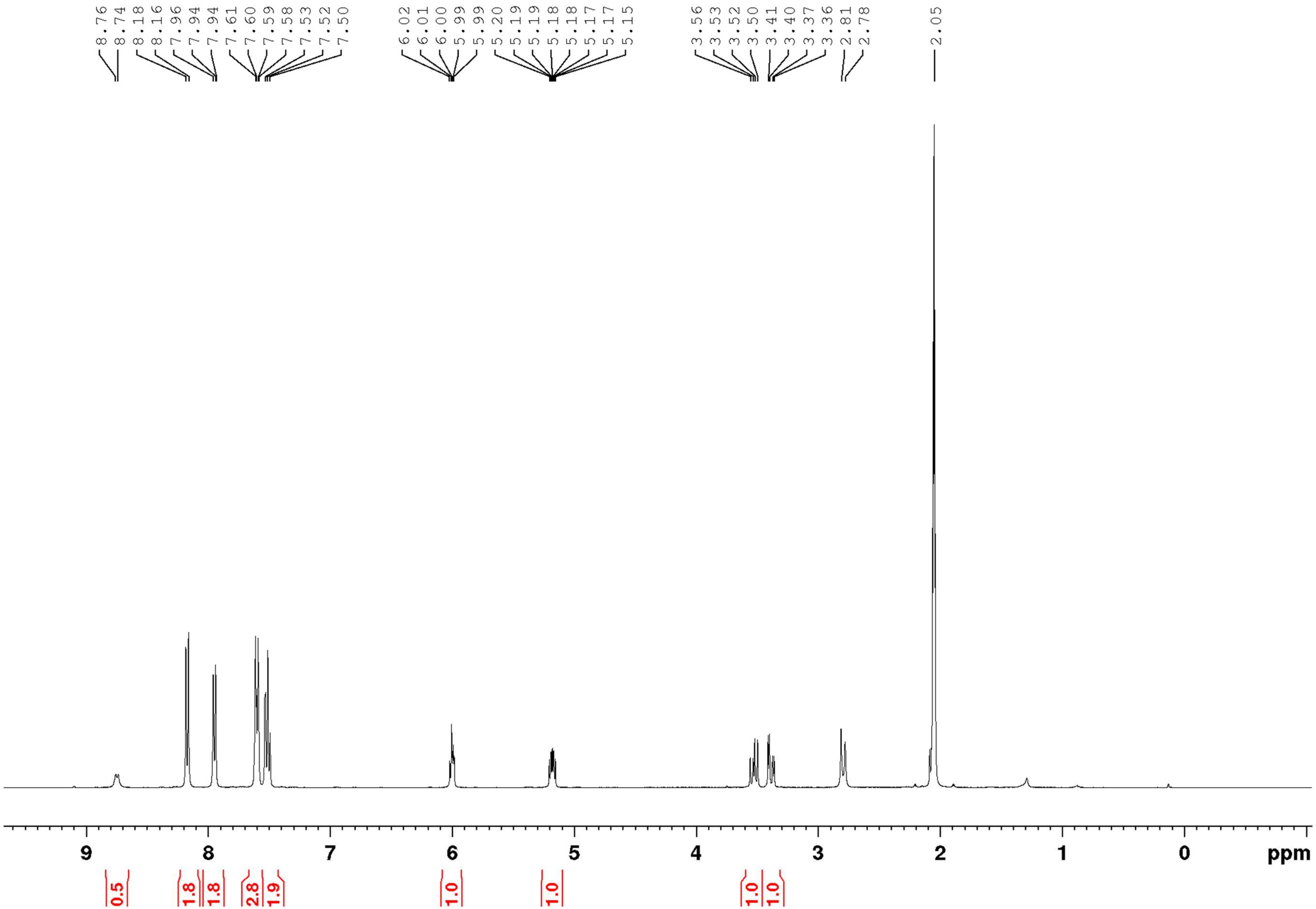

Figure S35: ^{1}H NMR of **4** in acetone-d_6 at 298 K.

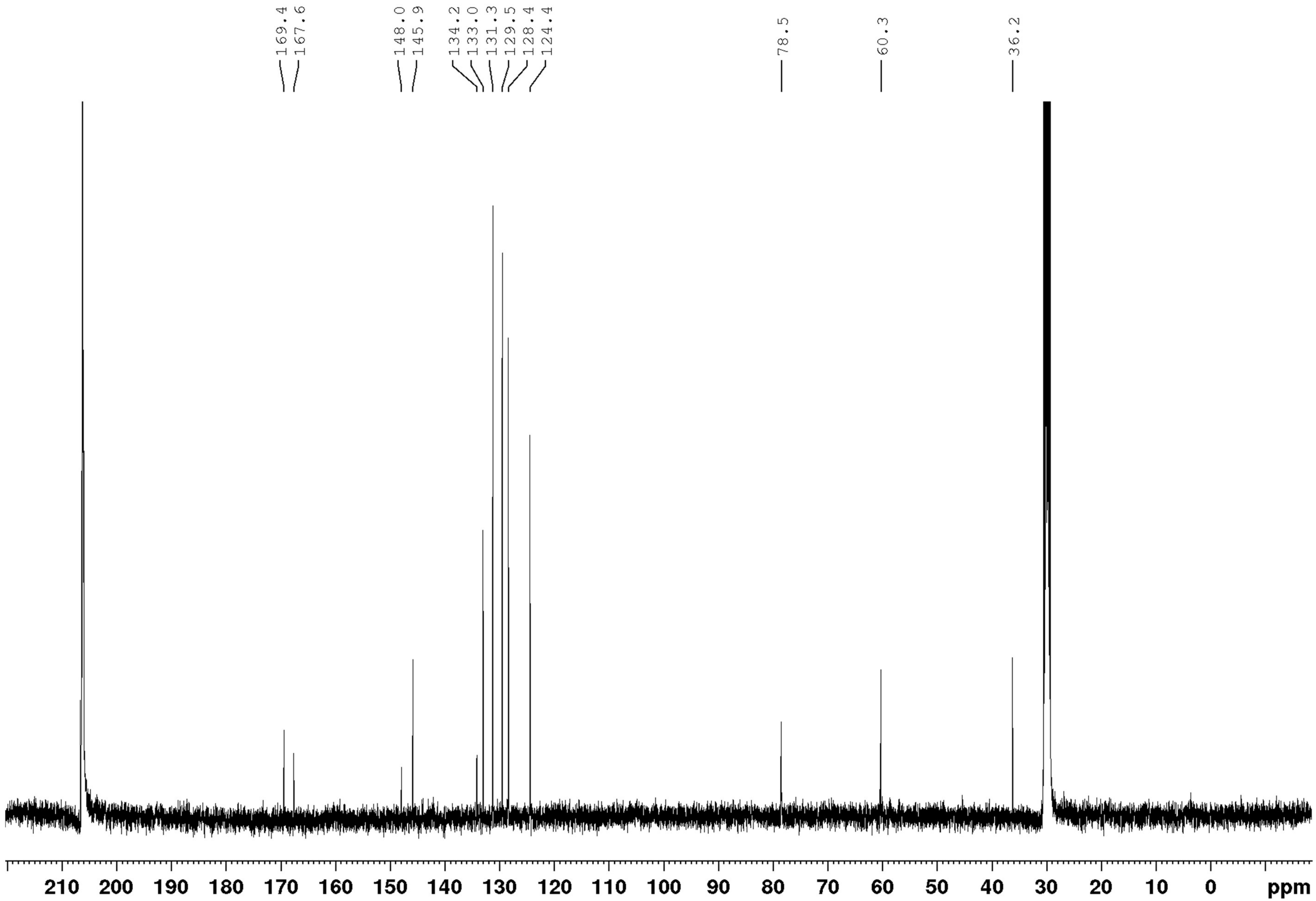

Figure S36: ^{13}C NMR of **4** in acetone-d_6 at 298 K.

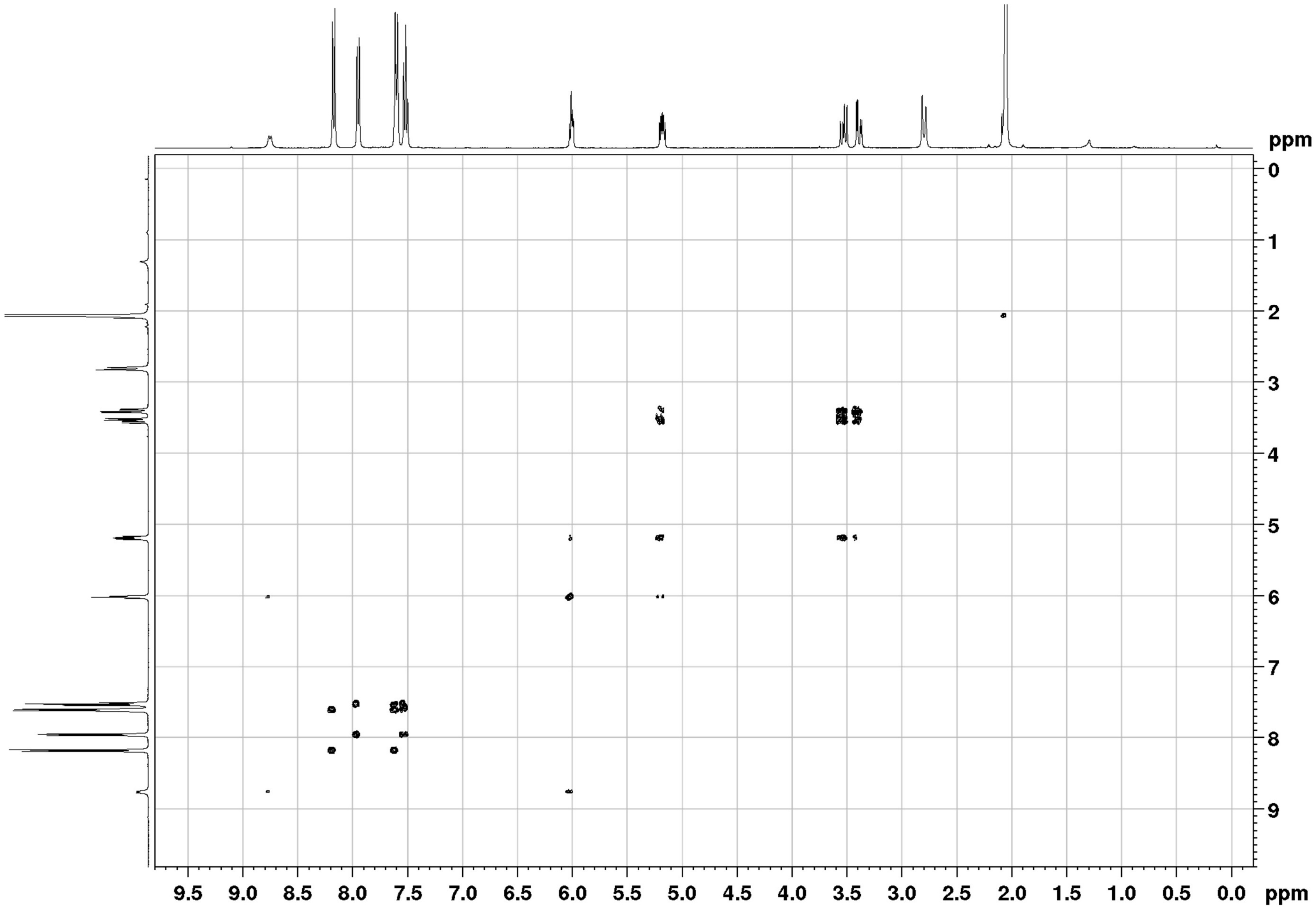

Figure S37: COSY NMR for **4** in acetone-d_6 at 298 K.

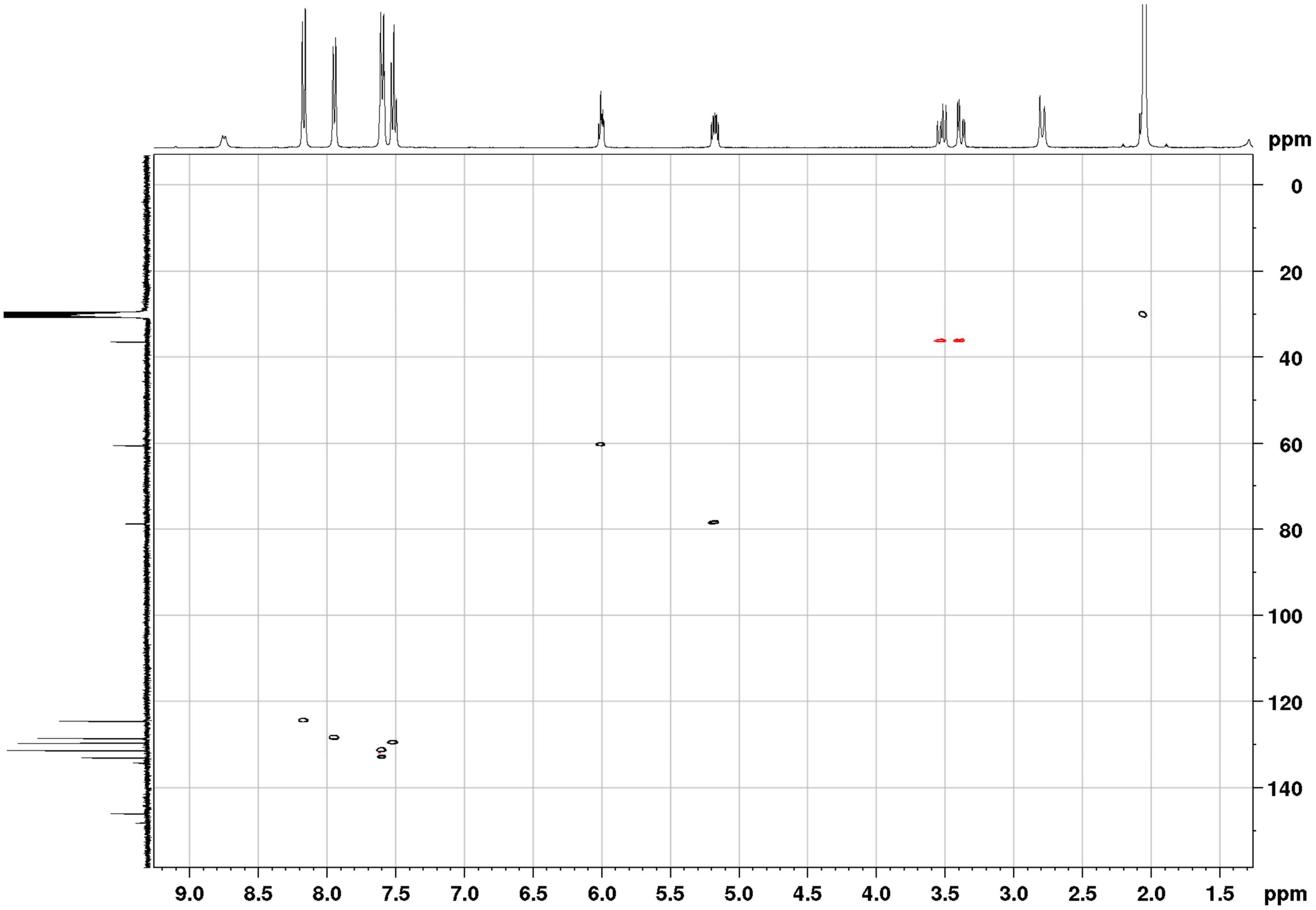

Figure S38: HSQC-edited NMR of **4** in acetone-d_6 at 298 K.

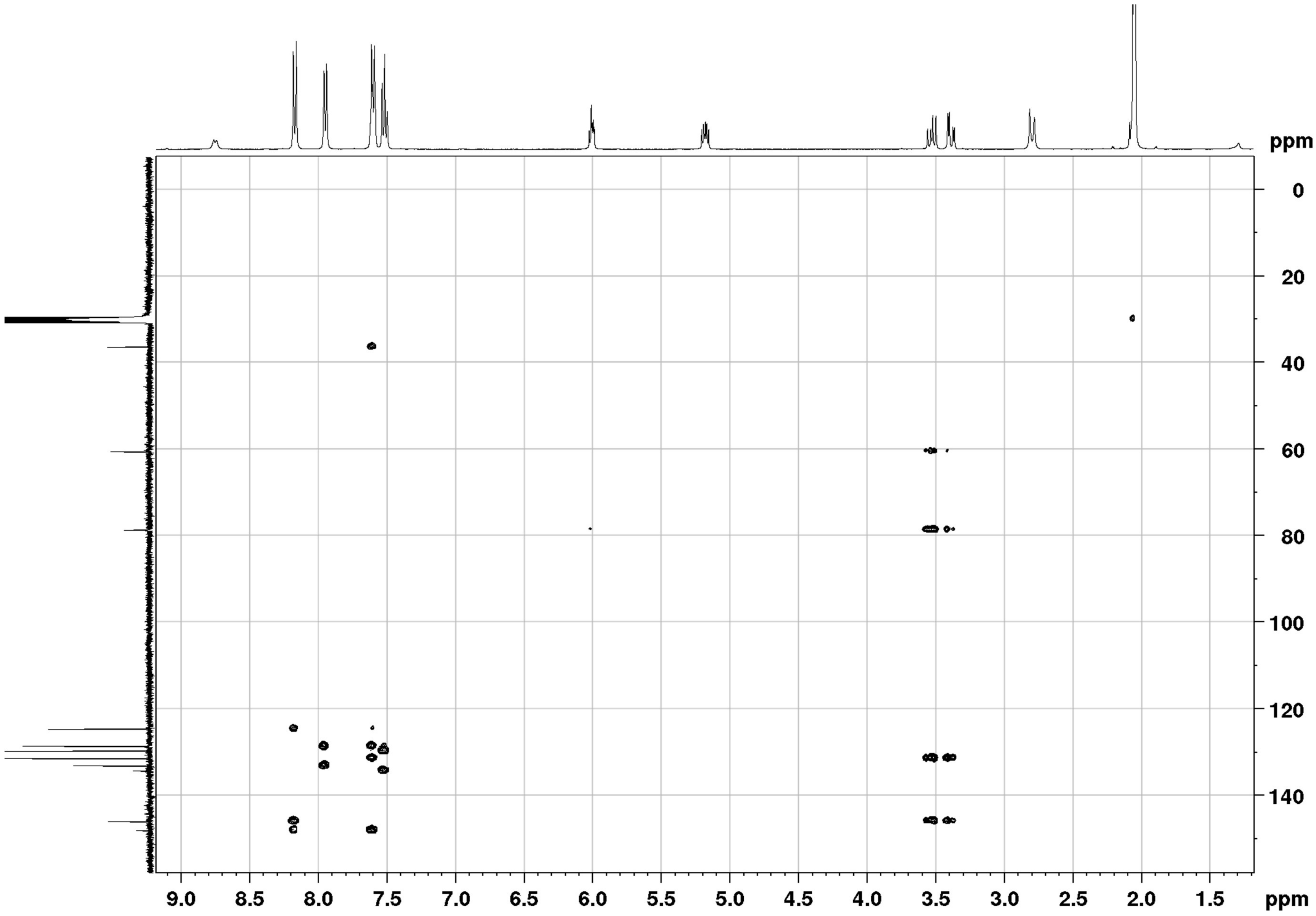

Figure S39: HMBC NMR of **4** in acetone-d_6 at 298 K.

Hydrolysed obafluorin (5)

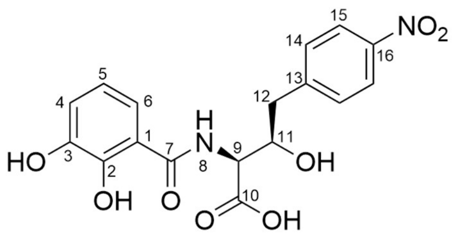

Table S6: Resonances assignment in ^{1}H and ^{13}C NMR spectra of **5** in acetone-d_6 at 298 K.

Position	δ_H (no. of protons, multiplicity, *J* in Hz)	δ_c	COSY	HMBC
1		115.5		
2		150.6		
3		147.4		
4	7.44-7.40 (1H, m)	118.4	5	2, 6, 7
5	6.83-6.78 (1H, m(	119.5	4, 6	1, 3, 6
6	7.05-7.02 (1H, m)	119.9	5	2, 3, 4
7		171.4		
8	7.92-7.86 (1H, m)*		9	2, 3, 6
9	4.86 (1H, dd, 8.9 & 2.4)	57.2	8	10
10	CO_2<u>H:</u> 11.27 (1H, bs)	171.7		
11	4.68-4.61 (1H, m)	73.0	11-OH (weak), 12	
12	3.16 (1H, dd, 13.6 & 5.1)<br>3.06 (1H, dd, 13.6 & 8.5)	41.3	11, 12	9, 11, 13, 14
13		147.8		
14	7.63 (2H, d, 8.6)	131.7	15	12, 14, 15, 16
15	8.18 (2H, d, 8.6)	124.2	14	15, 16
16		147.8		
2-OH	12.25 (1H, s)			
3-OH	7.92-7.86 (1H, m)*			
11-OH	4.79-4.74 (1H, m)		11 (weak)	11

*H-8 and 3-OH are under the same signal.

Note that there was some formic acid from purification in the sample used for ^{13}C and HMBC (δ_H 8.10 and δ_C 162.4).[7]

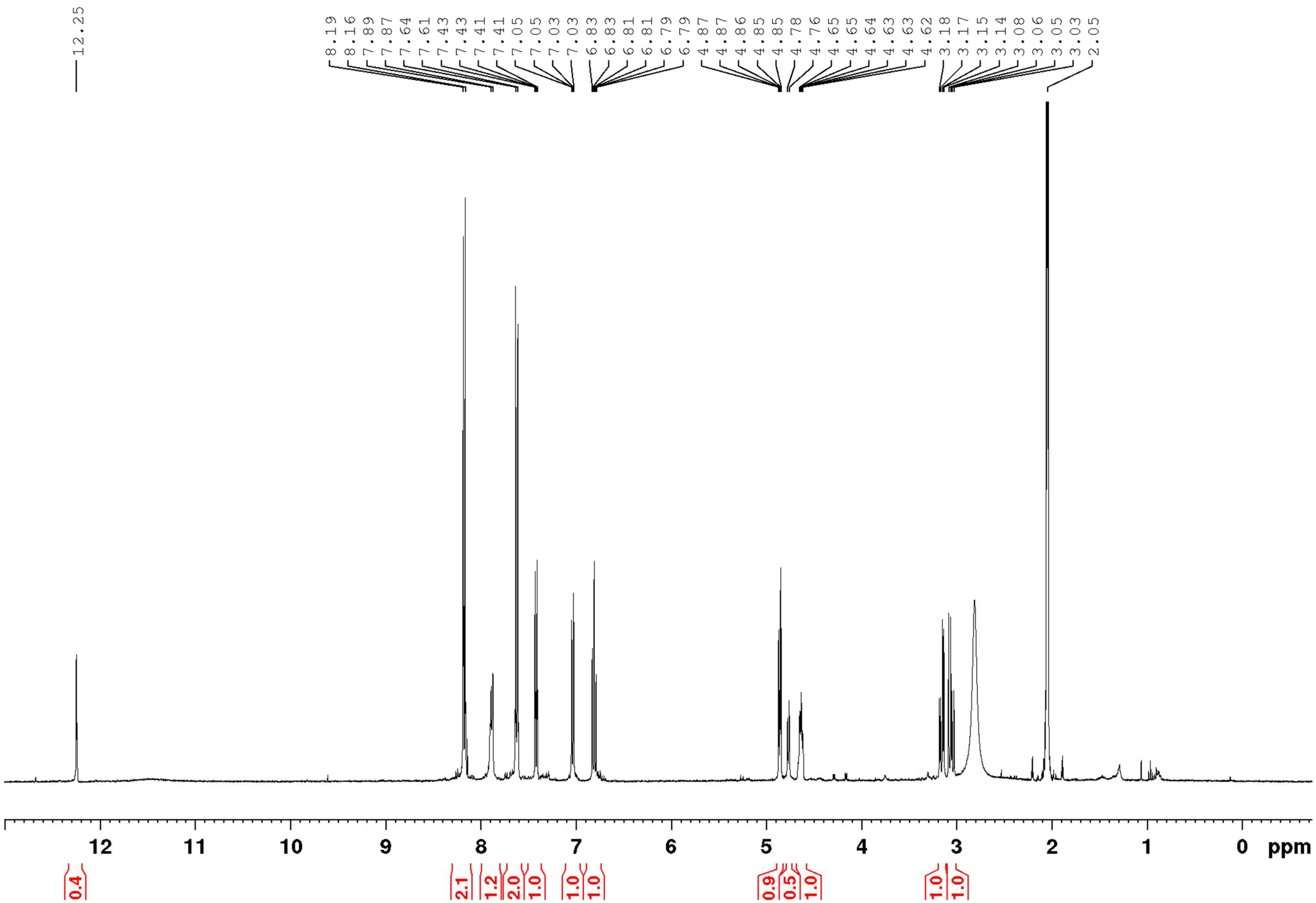

Figure S40: ^{1}H NMR of **5** in acetone-d_6 at 298 K.

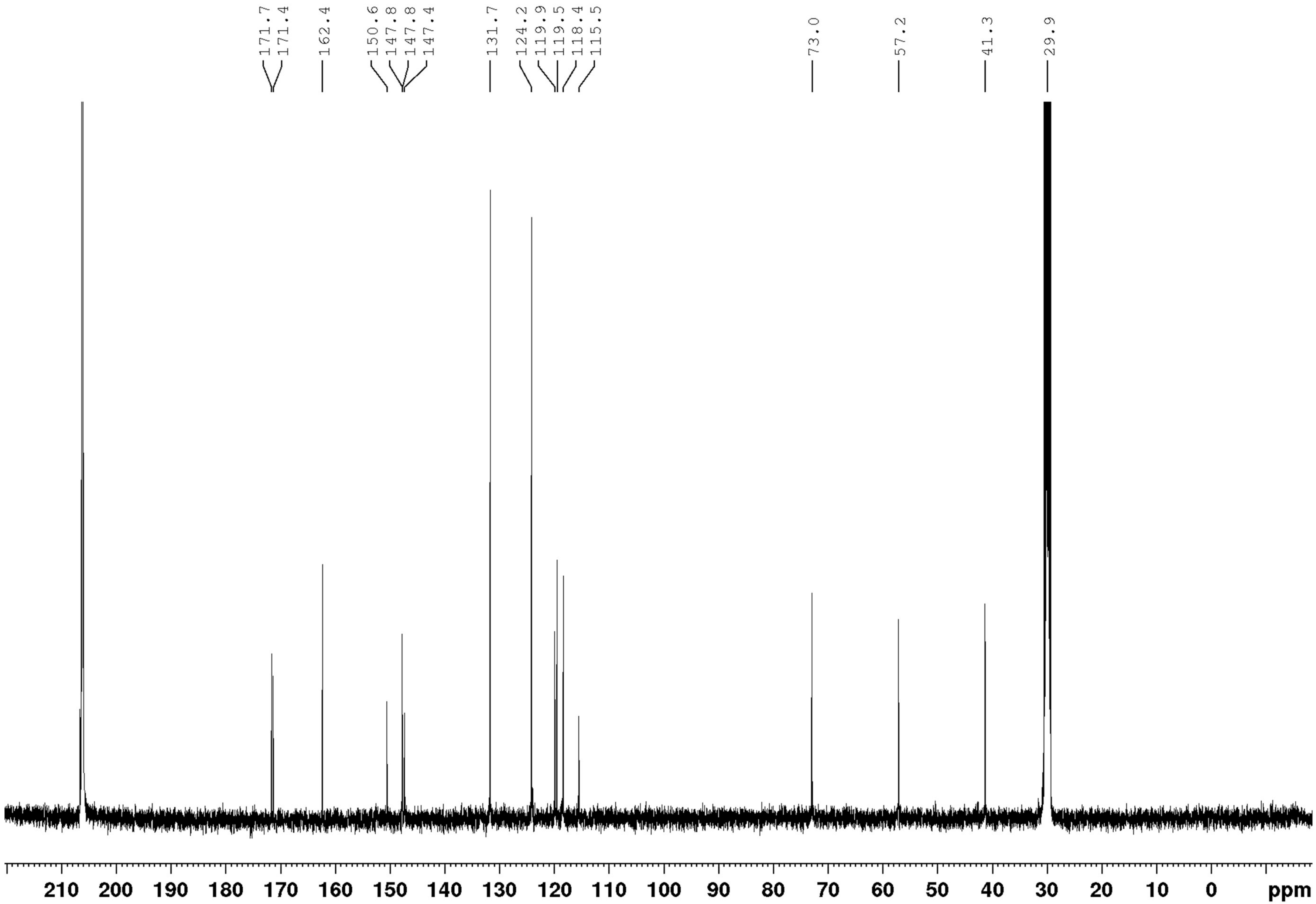

Figure S41: ^{13}C NMR of **5** in acetone-d_6 at 298 K.

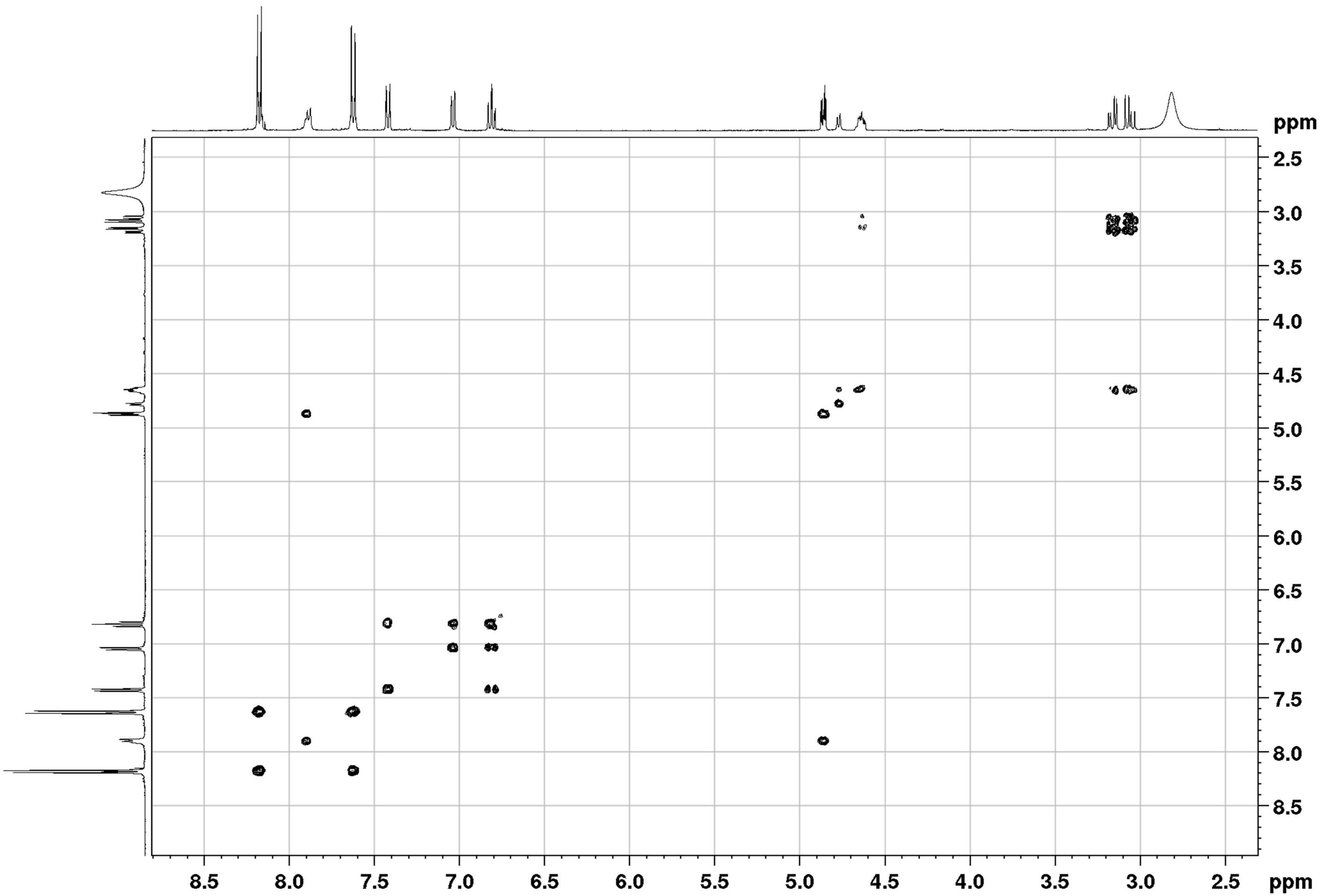

Figure S42: COSY NMR of **5** in acetone-d_6 at 298 K.

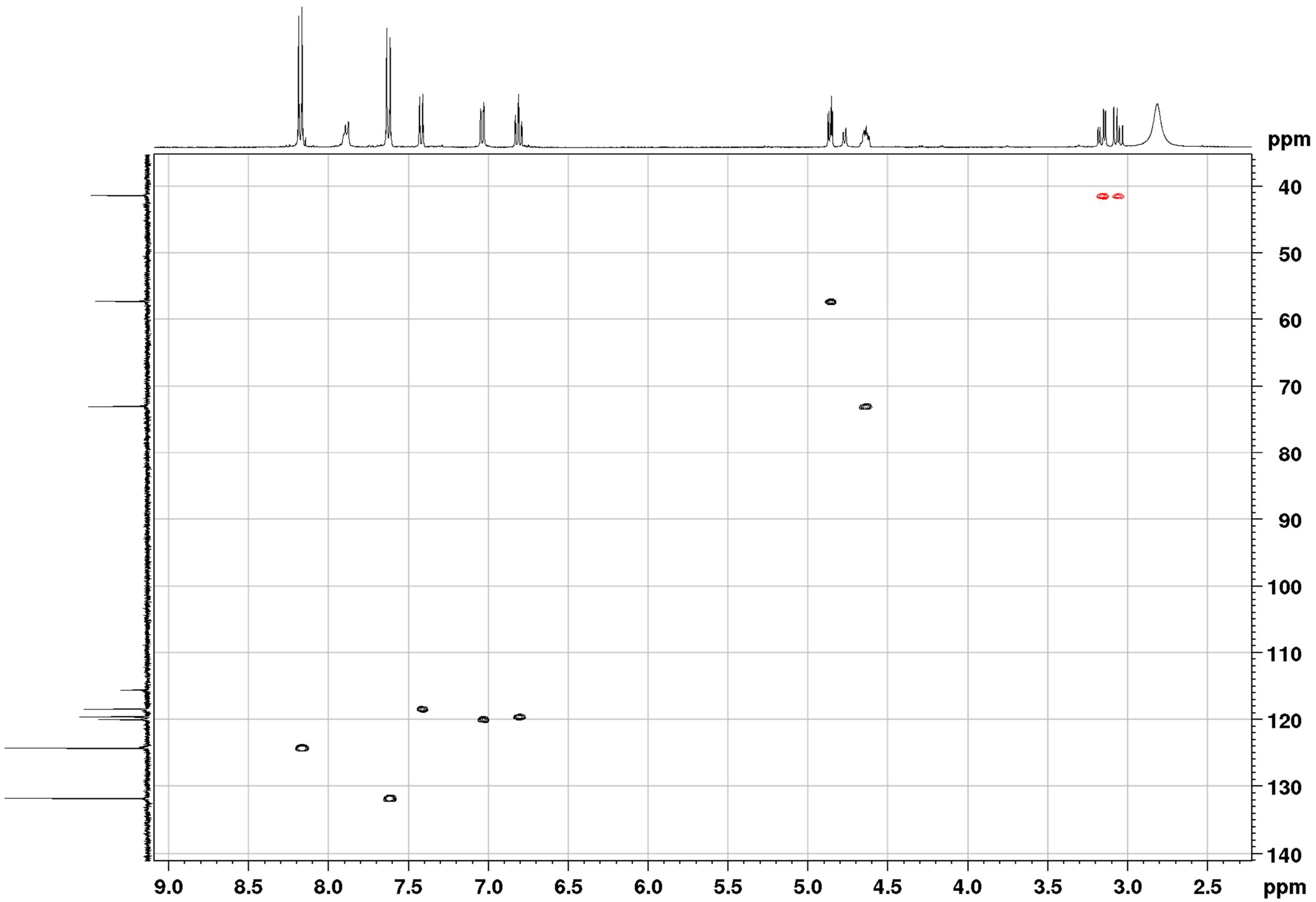

Figure S43: HSQC-edited NMR for **5** in acetone-d_6 at 298 K.

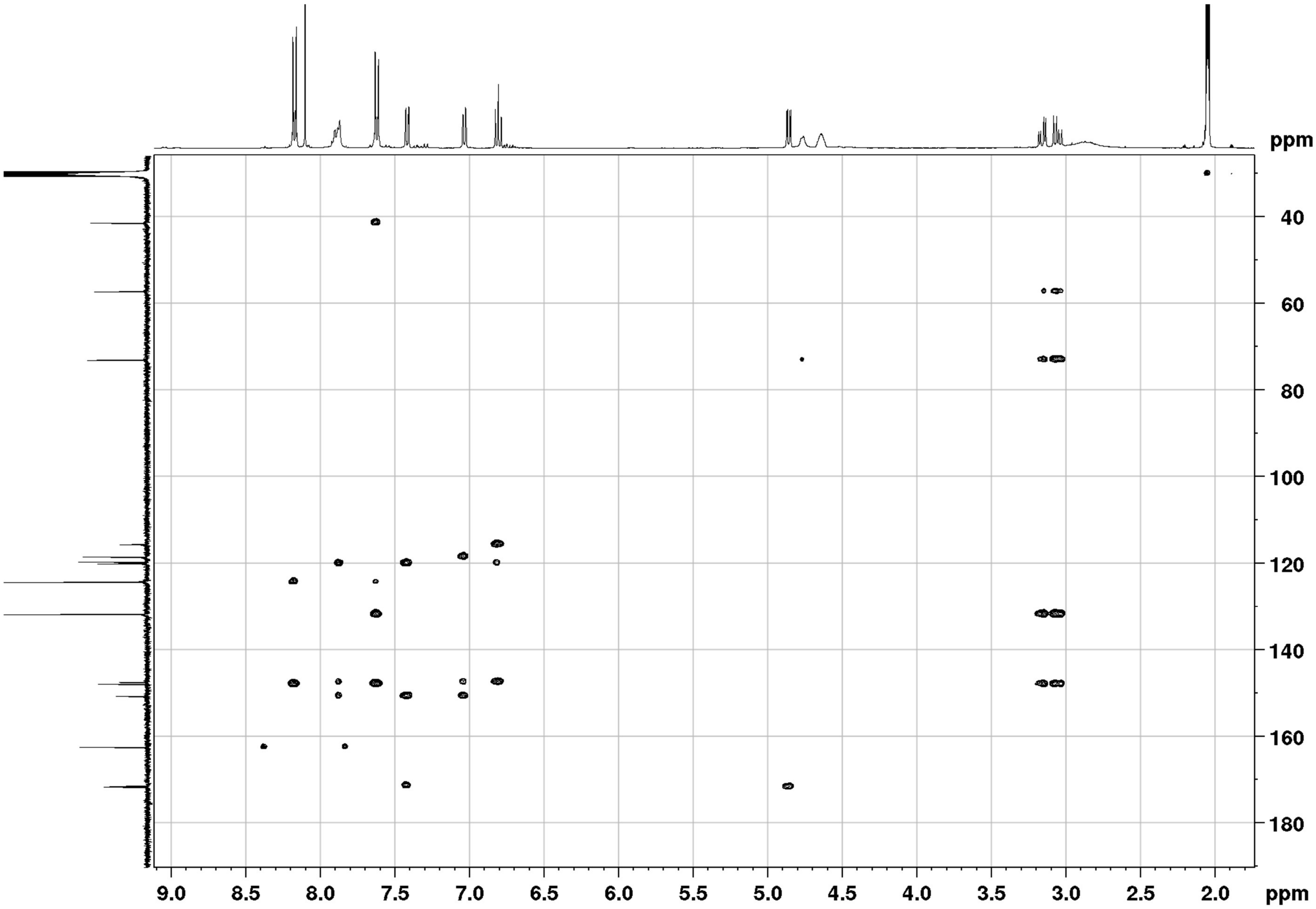

Figure S44: HMBC NMR for **5** in acetone-d_6 at 298 K.

Methanolysed obafluorin (6)

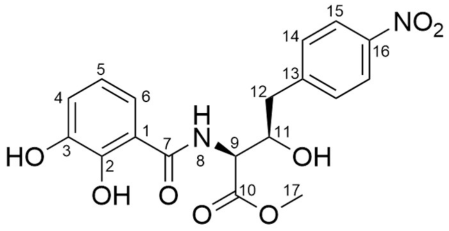

Table S7: Resonances assignment in ^{1}H and ^{13}C NMR spectra of **6** in acetone-d_6 at 298 K.

Position	δ_H (no. of protons, multiplicity, *J* in Hz)	δ_c	COSY	HMBC
1		115.6		
2		150.5		
3		147.4		
4	7.43 (1H, dd, 8.2 & 1.4)	118.5	5	2, 6, 7
5	6.81 (1H, dd, 8.2 & 7.9)	119.5	4, 6	1, 3, 6
6	7.04 (1H, dd, 7.9 & 1.0	120.0	5	2, 3, 4
7		171.3		
8	7.98 (1H, d, 8.6)		9	
9	4.86 (1H, dd, 8.6 & 2.6)	57.5	8	10, 11 (weak)
10		171.3		
11	4.62-4.56 (1H, m)	72.9	11-OH, 12	
12	3.14 (1H, dd, 13.8 & 5.1)<br>3.04 (1H, dd, 13.8 & 8.4)	41.4	11, 12	9, 11, 13, 14
13		147.7		
14	7.61 (2H, d, 8.6)	131.7	15	12, 13, 14, 15
15	8.17 (2H, d, 8.8)	124.2	14	15, 16
16		147.7		
17	3.72 (3H, s)	52.8		10
2-OH	12.14 (1H, s)			
3-OH	7.92 (1H, s)			2, 3, 5
11-OH	4.82-4.74 (1H, m)		11	9, 11, 12,

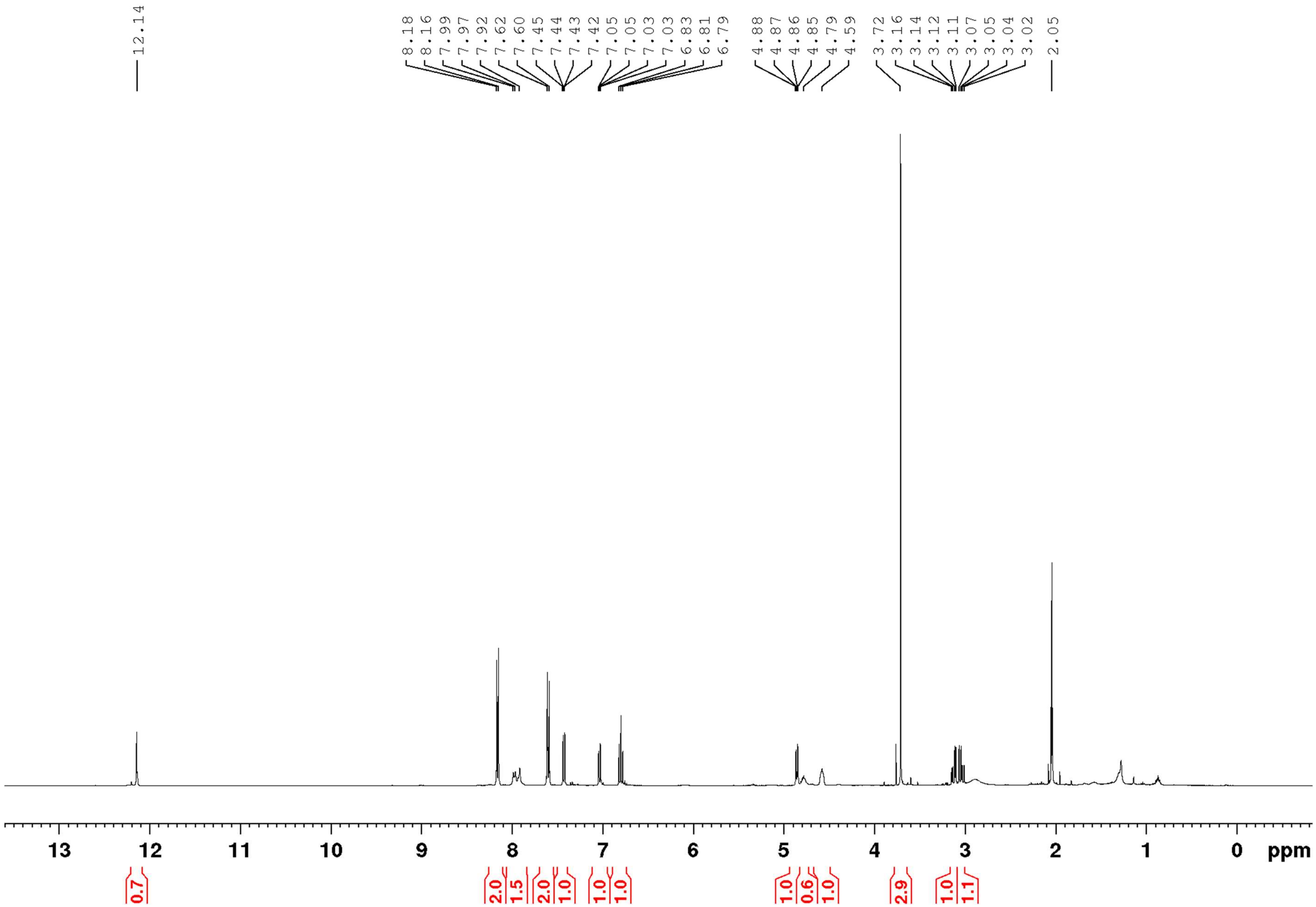

Figure S45: ^{1}H NMR for **6** in acetone-d_6 at 298 K.

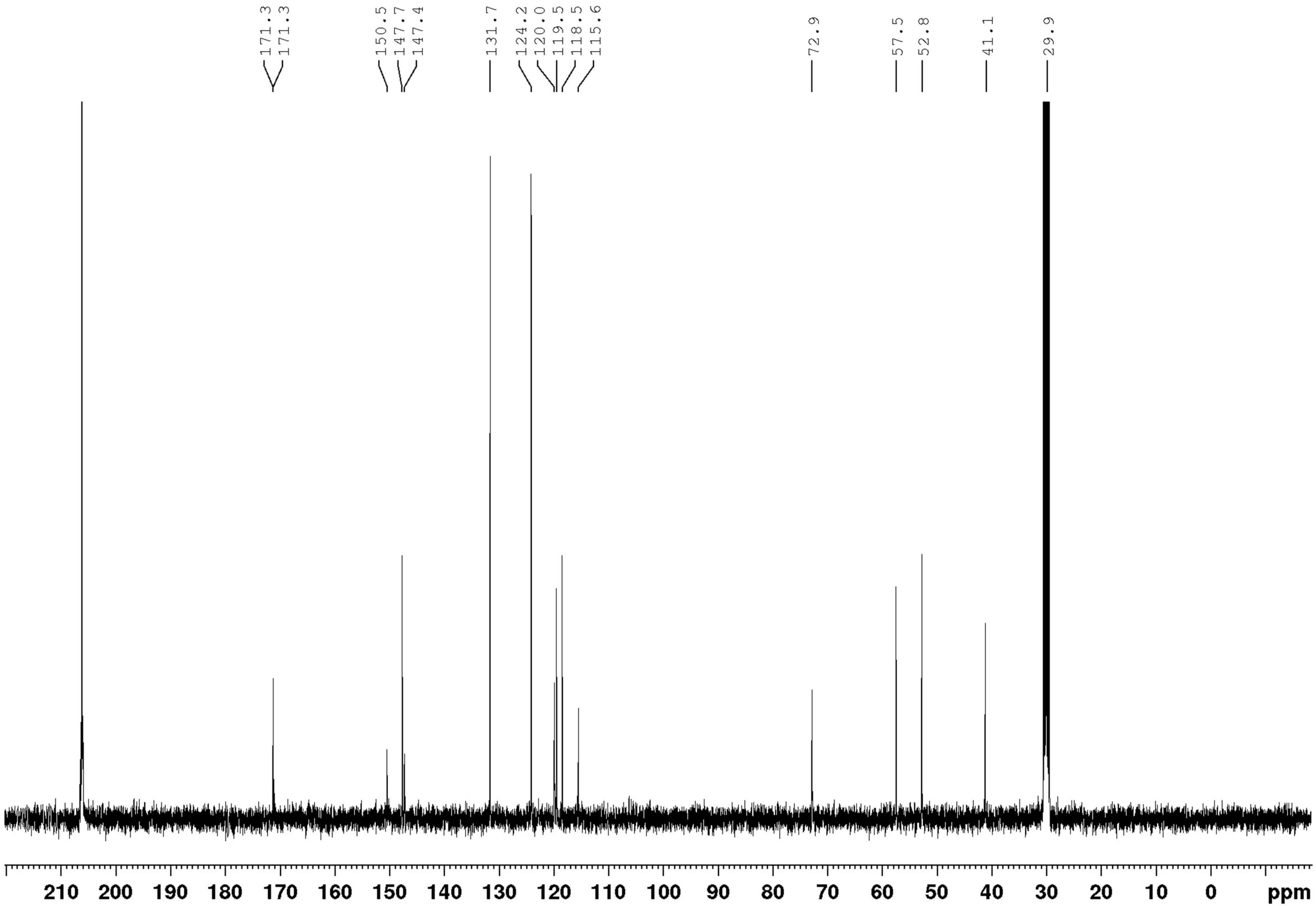

Figure S46: ^{13}C NMR for **6** in acetone-d_6 at 298 K.

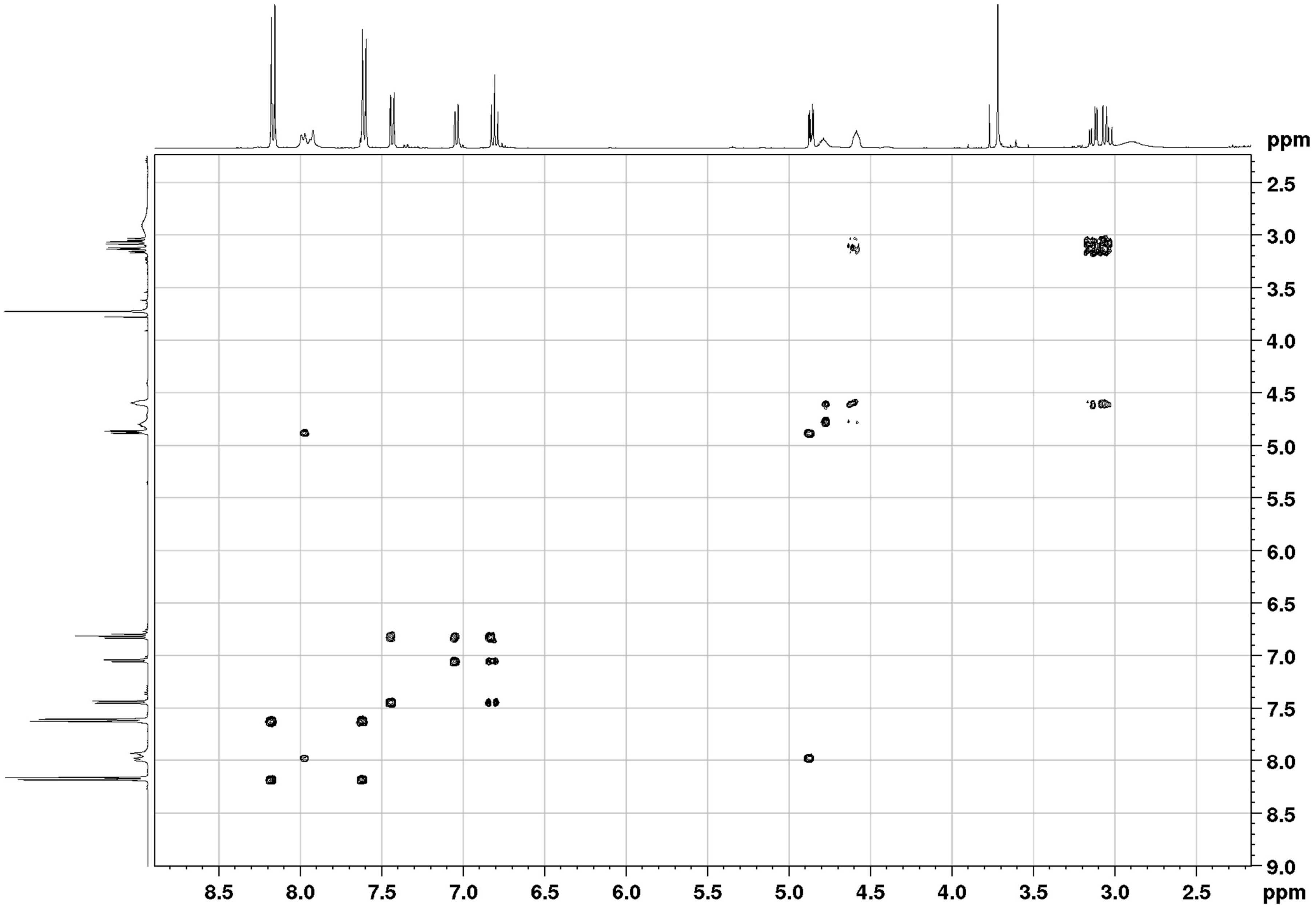

Figure S47: COSY NMR for **6** in acetone-d_6 at 298 K.

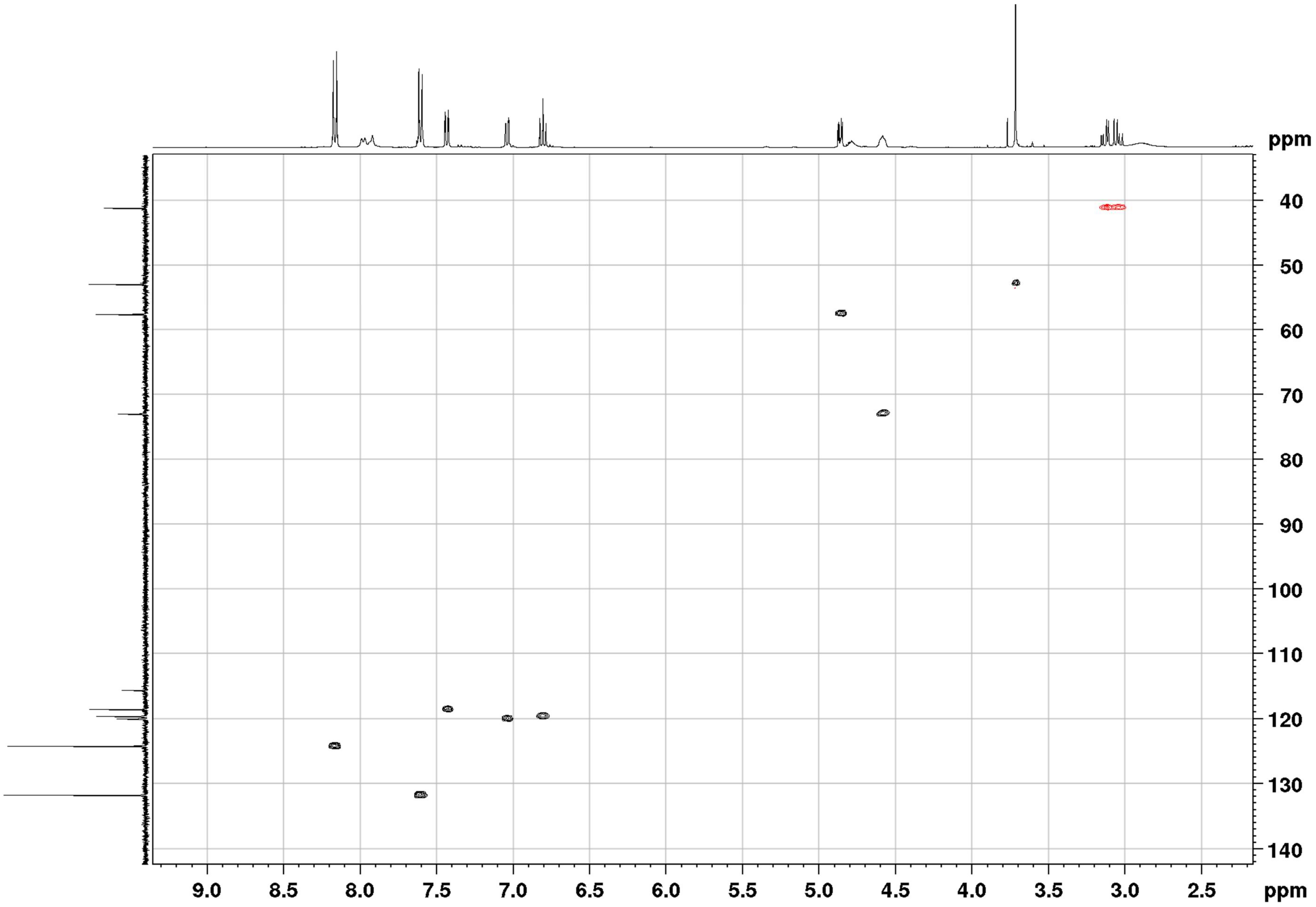

Figure S48: HSQC-edited NMR for **6** in acetone-d_6 at 298 K.

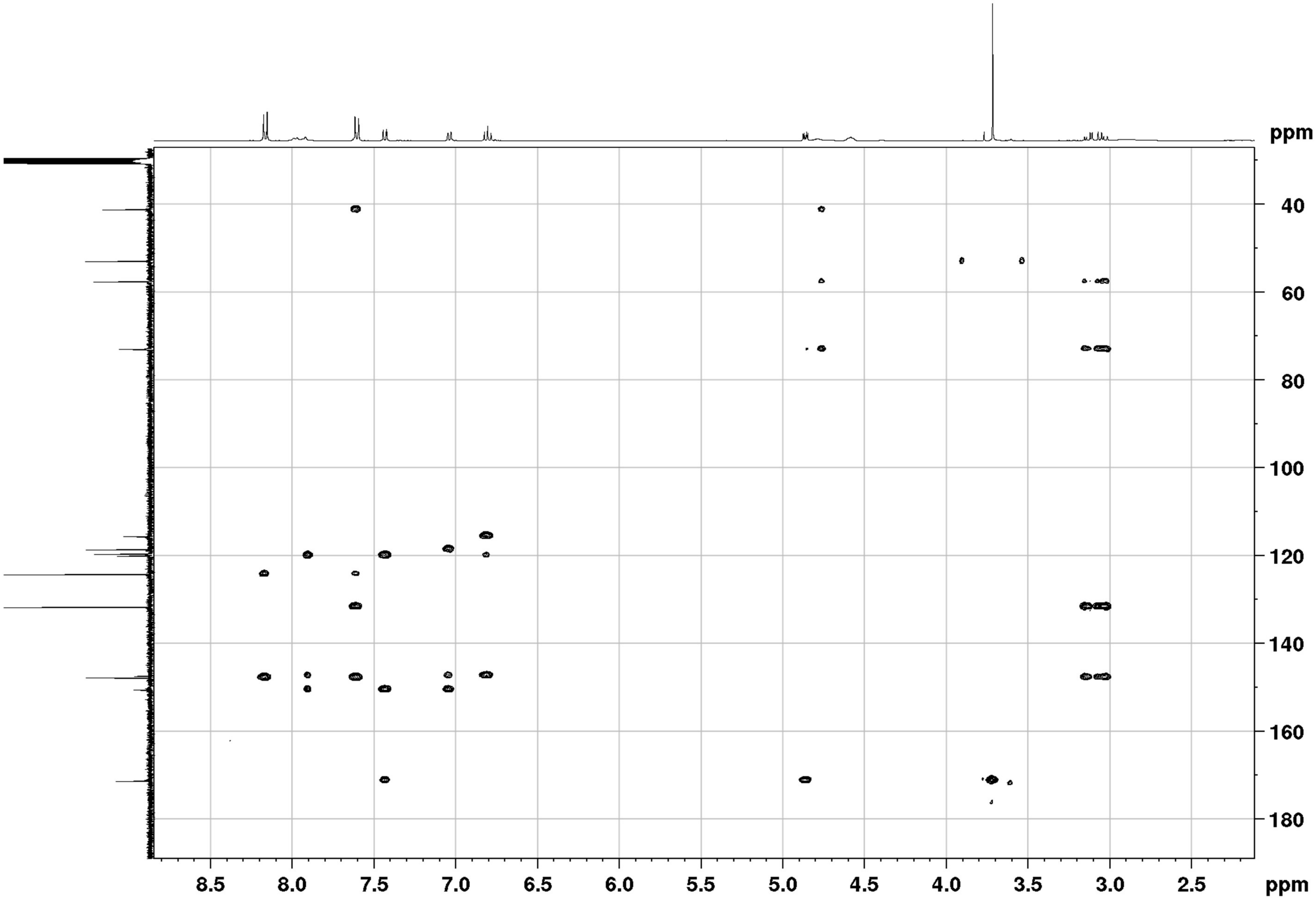

Figure S49: HMBC NMR for **6** in acetone-d_6 at 298 K.

Hydrolysed BA-obafluorin (7)

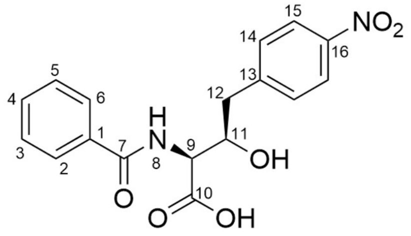

Table S8: Resonances assignment in ^{1}H and ^{13}C NMR spectra of **7** in acetone-d$_6$ at 298 K.

Position	δ_H (no. of protons, multiplicity, *J* in Hz)	δ_c	COSY	HMBC
1		132.4		
2/6	7.98 (2H, dd, 8.4 & 1.4)	128.3	3/5	2/6, 4, 7
3/5	7.56-7.49 (2H, m)*	129.4	2/6	1, 3/5
4	7.60-7.55 (1H, m)*	132.5		2/6
7		168.0		
8	7.56-7.51 (1H, m)*		9	
9	4.85 (1H, dd, 8.9 & 2.5)	57.5	8, 11	7 (weak), 10, 11
10		172.3		
11	4.62-4.58 (1H, m)	73.3	9, 12	
12	3.14 (1H, dd, 13.7 & 5.1)<br>3.03 (1H, dd, 13.7 & 8.3)	41.4	11, 12	9, 11, 13, 14
13		148.1		
14	7.62 (2H, d, 8.8)	131.7	15	14, 15, 16
15	8.17 (2H, d, 8.8)	124.1	14	13, 15
16		147.7		
10-OH	NS			
11-OH	NS			

*H-3/5, H-4 and H-8 make up a multiplet from 7.60-7.49 ppm. More accurate chemical shift ranges were found from HSQC-edited (H-3/5 and H-4) and COSY (H-8) NMR.

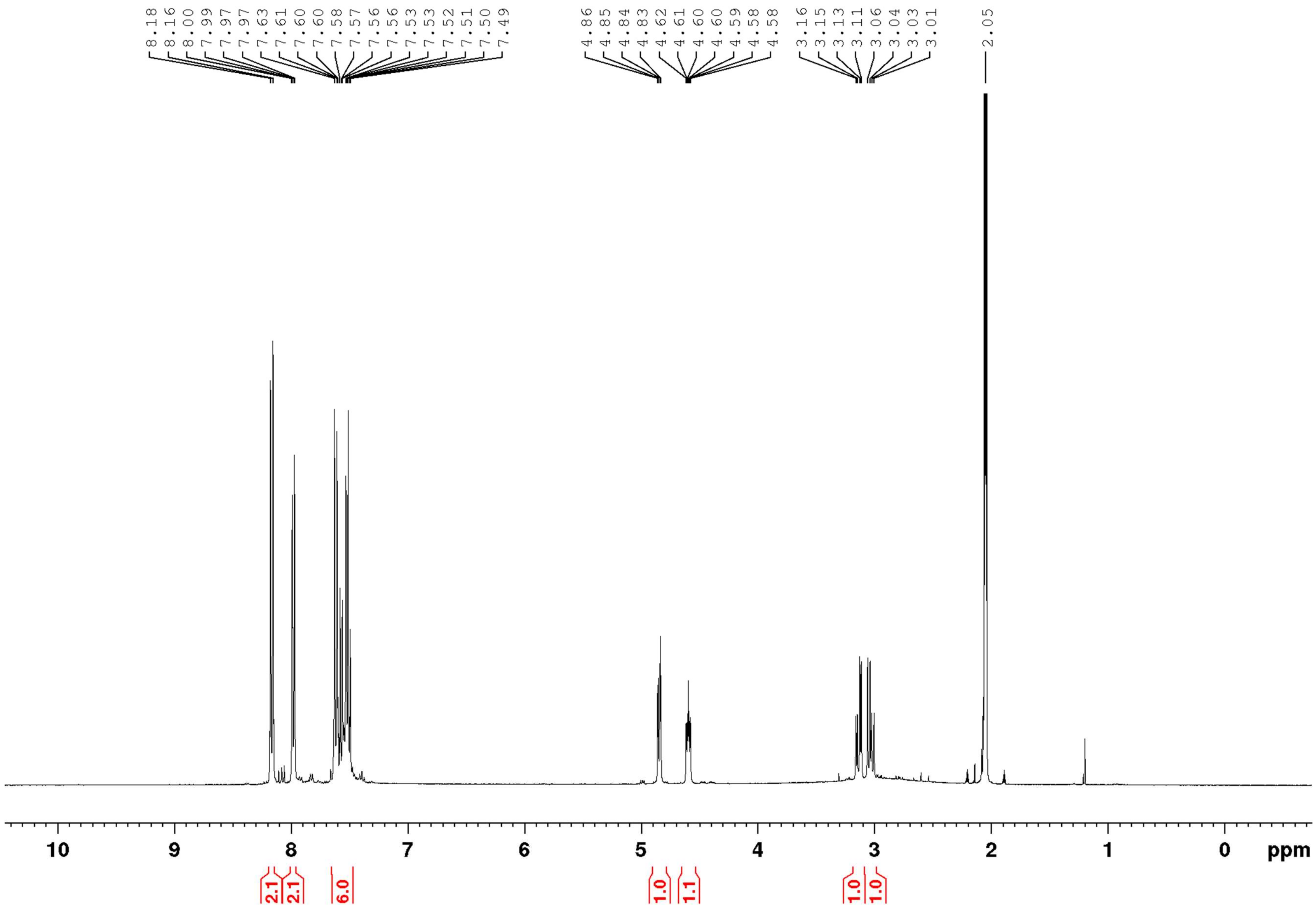

Figure S50: ^{1}H NMR for **7** in acetone-d_6 at 298 K.

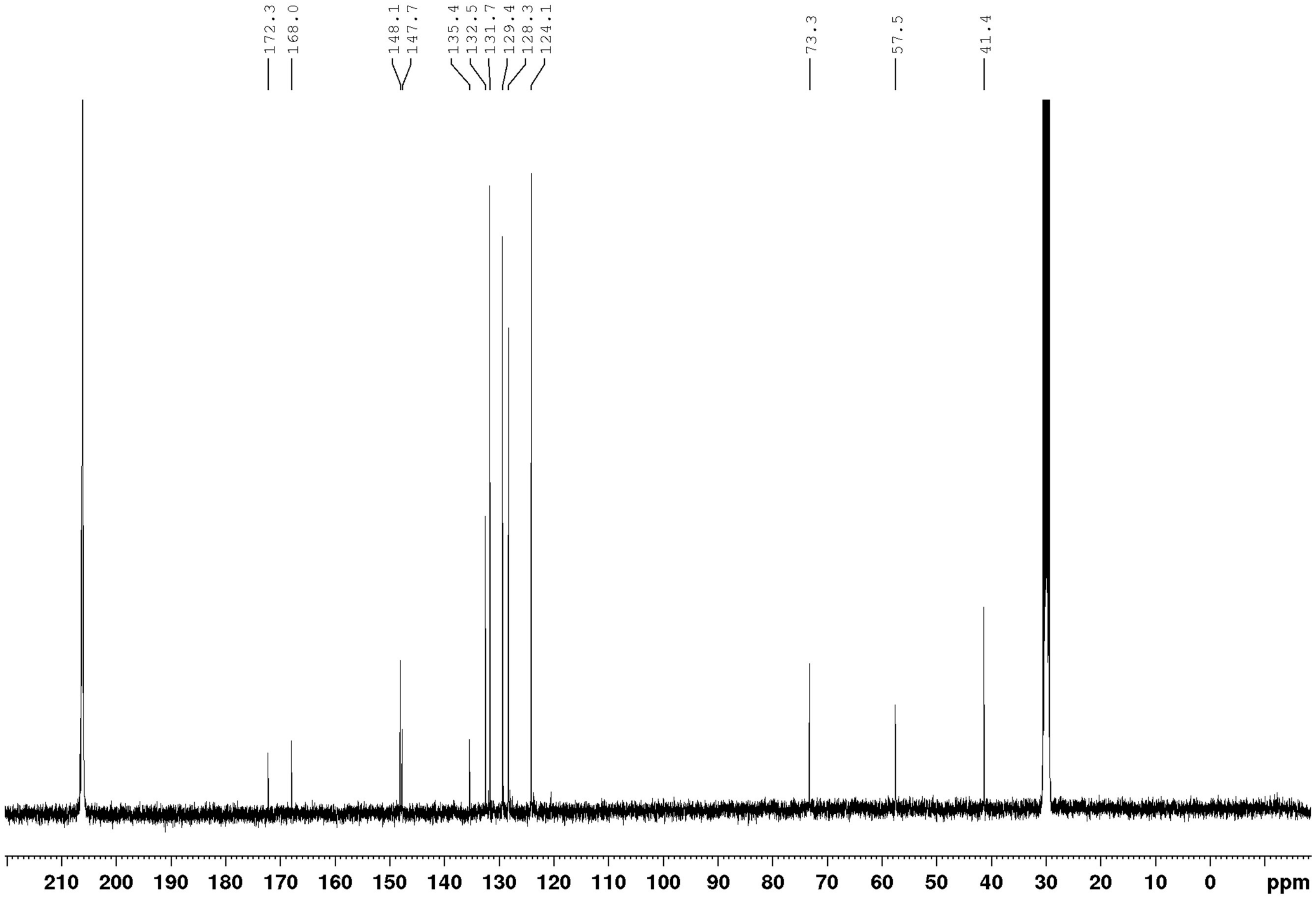

Figure S51: ^{13}C NMR for **7** in acetone-d_6 at 298 K.

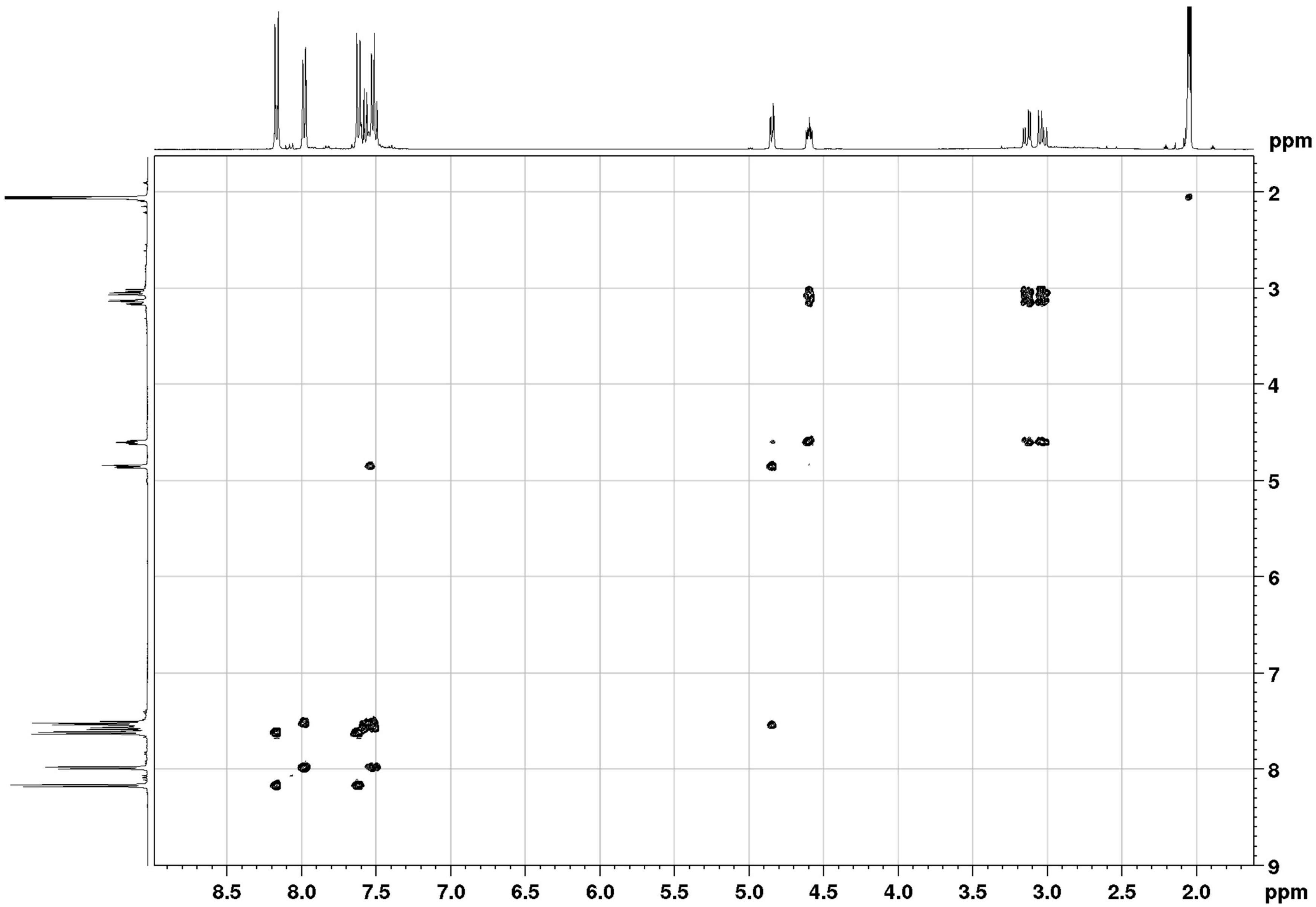

Figure S52: COSY NMR for **7** in acetone-d_6 at 298 K.

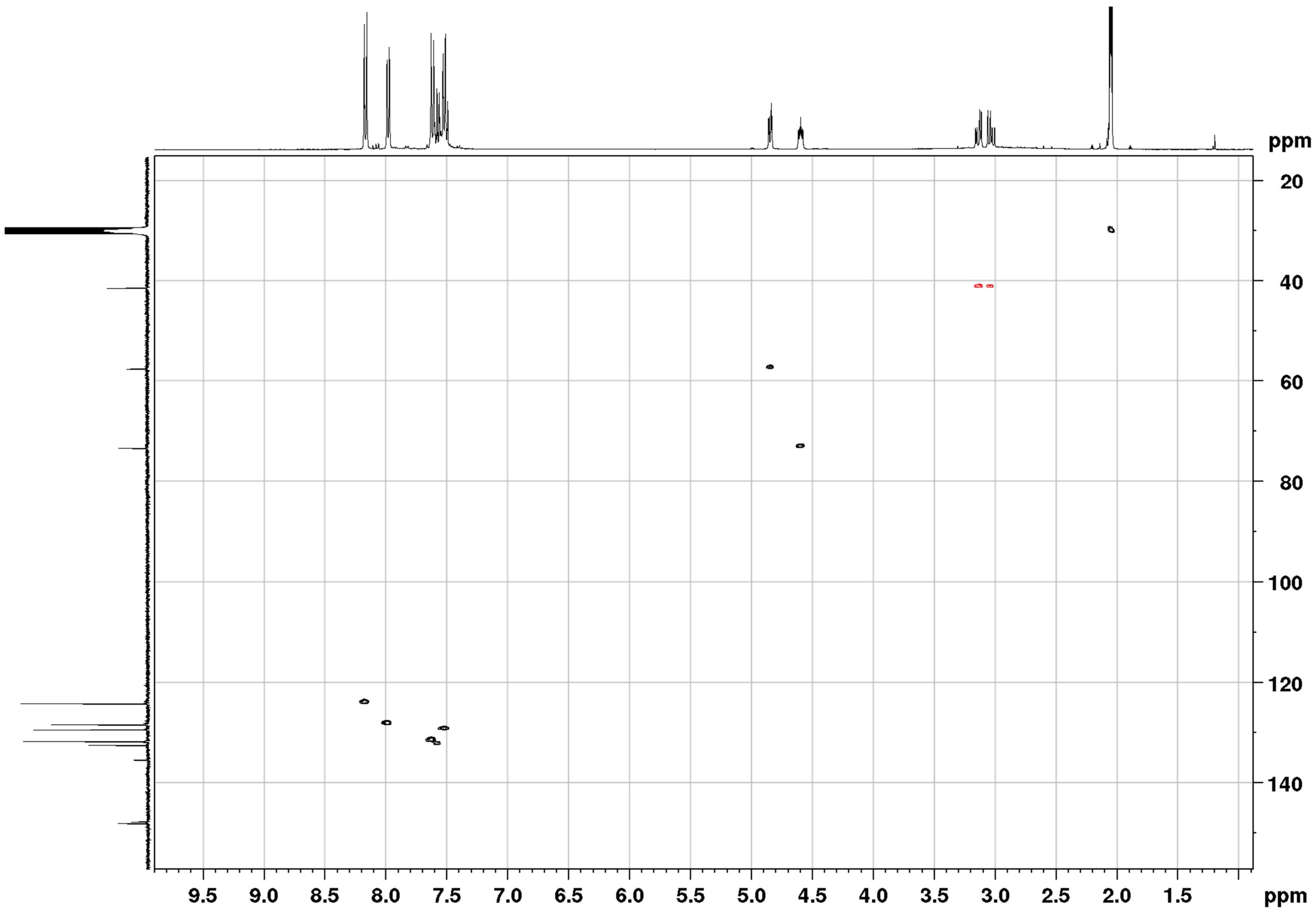

Figure S53: HSQC-edited NMR for **7** in acetone-d_6 at 298 K.

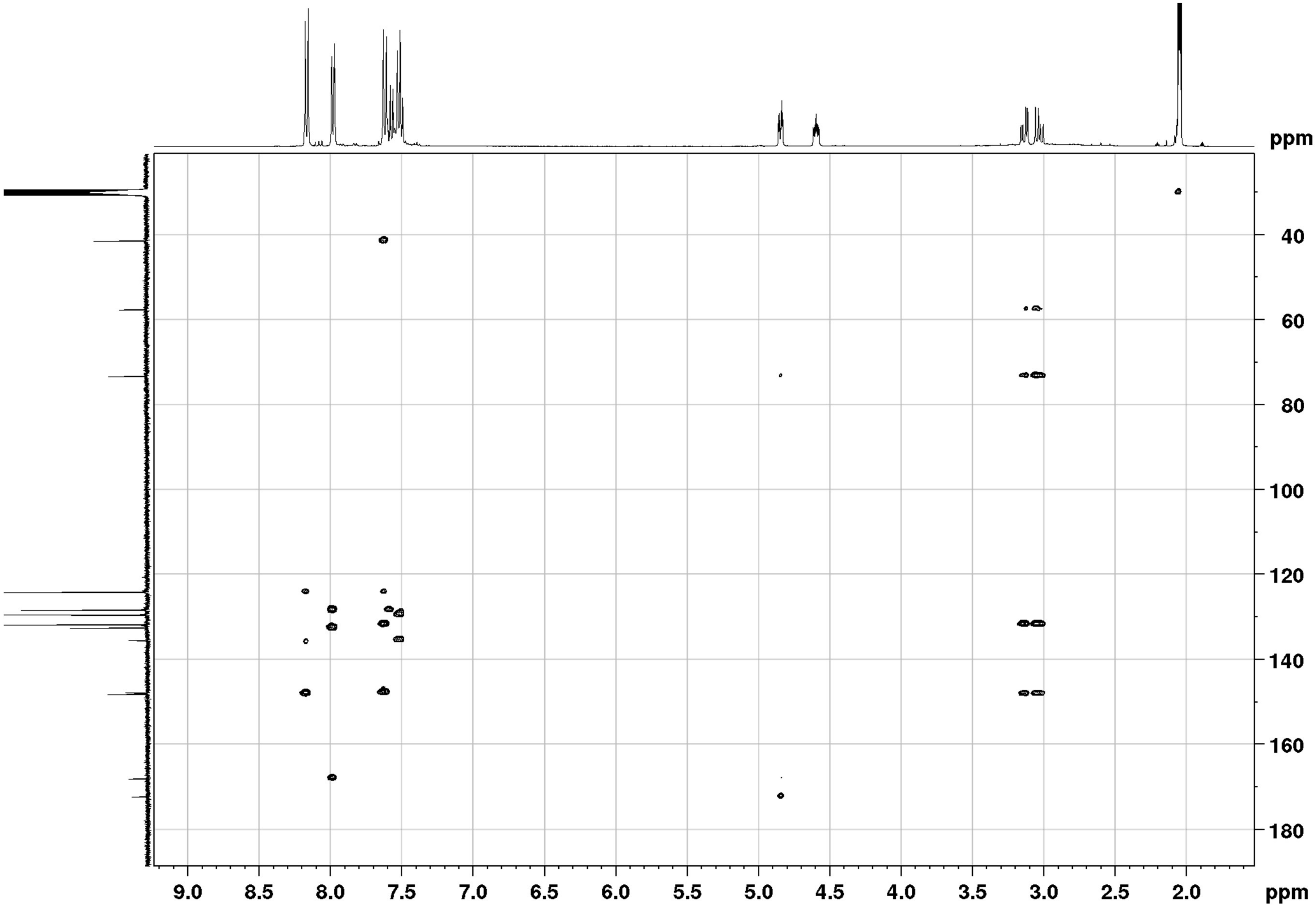

Figure S54: HMBC NMR for **7** in acetone-d_6 at 298 K.

References

1. B. Schwyn and J. B. Neilands, Universal chemical assay for the detection and determination of siderophores, *Anal. Biochem.*, 1987, **160**, 47–56.
2. K. N. Raymond, B. E. Allred and A. K. Sia, Coordination chemistry of microbial iron transport, *Acc. Chem. Res.*, 2015, **48**, 2496–2505.
3. K. R. Reid, M. E. Meyerhoff and J. T. Mitchell-Koch, Salicylate detection by complexation with iron(III) and optical absorbance spectroscopy., *J. Chem. Educ.*, 2008, **85**, 1658.
4. J. S. Renny, L. L. Tomasevich, E. H. Tallmadge and D. B. Collum, Method of continuous variations: applications of Job plots to the study of molecular associations in organometallic chemistry, *Angew. Chemie Int. Ed.*, 2013, **52**, 11998–12013.
5. H. M. D. Bandara, D. P. Kennedy, E. Akin, C. D. Incarvito and S. C. Burdette, Photoinduced release of Zn^{2+} with ZinCleav-1: a nitrobenzyl-based caged complex, *Inorg. Chem.*, 2009, **48**, 8445–8455.
6. T. A. Scott, S. F. D. Batey, P. Wiencek, G. Chandra, S. Alt, C. S. Francklyn and B. Wilkinson, Immunity-guided identification of threonyl-tRNA synthetase as the molecular target of obafluorin, a β-lactone antibiotic, *ACS Chem. Biol.*, 2019, **14**, 2663–2671.
7. N. Babij, E. O. McCusker, G. T. Whiteker, B. Canturk, N. Choy, L. C. Creemer, C. V. De Amicis, N. M. Hewlett, P. L. Johnson, J. A. Knobelsdorf, F. Li, B. A. Lorsbach, B. M. Nugent, S. J. Ryan, M. R. Smith, and Q. Yang, NMR Chemical Shifts of Trace Impurities: Industrially Preferred Solvents Used in Process and Green Chemistry, *Org. Process Res. Dev.* 2016, **20**, 661–667.
